# P-glycoprotein 1 as a shared target for resensitizing drug-resistant tumor cells and preventing fibronectin-driven metastasis

**DOI:** 10.7150/thno.131375

**Published:** 2026-06-04

**Authors:** Li-Tzu Huang, Li-Hsin Cheng, Chin-Ho Kuo, Chin-Yun Cheng, Shane-Rong Sheu, Cheng-Han Yang, Pei-Chu Shih, Yi-Syuan Li, Lin Tseng, Wei-Ting Hsueh, Lyh-Jyh Hao, Hung-Chi Cheng

**Affiliations:** 1 The Institute of Basic Medical Sciences, College of Medicine, National Cheng Kung University, 1 University Road, Tainan 70101, Taiwan.; 2 Division of Hematology-Oncology, Department of Internal Medicine, Ditmanson Medical Foundation Chia-Yi Christian Hospital, Chiayi 600, Taiwan.; 3 The Institute of Biotechnology Research Center, Far East University, Tainan 74448, Taiwan.; 4 Department of Biochemistry and Molecular Biology, College of Medicine, National Cheng Kung University, 1 University Road, Tainan 70101, Taiwan.; 5 Department of Oncology, National Cheng Kung University Hospital, College of Medicine, National Cheng Kung University, Tainan 70456, Taiwan.; 6 Department of Endocrinology and Metabolism, Kaohsiung Veteran General Hospital, Tainan Branch, Tainan 71051, Taiwan.; 7 Department of Optometry, Chung Hwa University of Medical Technology, Tainan 71703, Taiwan.

**Keywords:** chemoresistance, P-glycoprotein 1, pericellular fibronectin assembly, metastasis, non-cytotoxic cancer therapy

## Abstract

**Rationale:**

The exacerbation of chemoresistance and metastasis by synthetic cytotoxic reagents hinders effective cancer therapy, as these events often coincide and lead to poor clinical outcomes, yet are rarely targeted through a shared molecular mechanism. To address this, we established a mechanism-informed natural compound discovery strategy to identify a non-cytotoxic candidate with dual functionality, namely re-sensitizing drug-resistant tumor cells and preventing metastasis.

**Methods:**

Western blot, RT-qPCR, and flow cytometry were used for evaluating protein and mRNA expression, as well as cell apoptosis, while GC/MS and HPLC analyses for identifying active phytochemicals from extracts of traditional Chinese medicines. Therapeutic potential was validated in multiple mouse cancer models, including K-ras^LSL-G12D/+^; p53^fl/fl^ mice. Clinical relevance was investigated via meta-analysis of associated gene signatures.

**Results:**

Mulberroside A (Mul A) from *Cortex Mori Radices* was identified as an ideal compound that inhibits P-glycoprotein 1 (Pgp1) in adherent tumor cells and pericellular fibronectin (periFN) assembly on suspended tumor cells (STCs), which drive drug resistance and metastasis, respectively. Using a paclitaxel (PTX)-resistant Lewis lung carcinoma cell line, we demonstrated that ERK-dependent Pgp1 functions as a shared upstream regulator of both chemoresistance and metastatic competence. Accordingly, Mul A inhibited *Pgp1* mRNA and protein levels in an ERK-dependent manner, thereby differentially restoring PTX sensitivity both *in vitro* and *in vivo*, without intrinsic cytotoxicity, and significantly inhibiting lung metastasis by reducing the Pgp1–XIAP–periFN axis in STCs. Oral administration of Mul A achieved these dual anti-cancer effects in both experimental and spontaneous mouse models. Importantly, meta-analysis of clinical datasets further linked co-elevated *FN* and* Pgp1* expression with poor prognosis and relapse in early-stage cancer patients, underscoring the translational relevance of targeting this shared pathway.

**Conclusions:**

These findings identify Mul A as a promising non-cytotoxic therapeutic candidate and elucidate the shared upstream molecular mechanism linking distinct downstream chemoresistance and metastasis.

## Introduction

Cancer is a chronic yet highly lethal disease worldwide, mainly due to the distant dissemination of circulating tumor cells (CTCs) derived from organs harboring long-established primary tumors 1, 2. The prevailing cancer therapies, either chemo/radiotherapies or targeted therapies, aim to trigger cell death pathways in cancer cells 3. Although short-term effects of these therapies are somewhat favourable for some cancer patients, long-term efficacy turned out to be disappointing due to the acquisition of drug resistance, often leading to distant metastasis 3-5. Alternatively, tumors may inherently acquire drug resistance during the course of metastatic cancer progression 6. These clinical dilemmas underscore an urgent need for strategies that simultaneously overcome drug resistance and inhibit metastasis 7.

Patients with innate or acquired drug resistance are often treated with second-line monotherapies; unfortunately, these approaches frequently induce cross-resistance or secondary mutations in recurrent tumors, exacerbating malignancy 8. Combination chemotherapy regimens have been developed as alternatives, but their synergistic or additive cytotoxicity, coupled with off-target interactions, often leads to intolerable side effects and still leaves cancer metastasis unsolved 9. Despite the emergence of next-generation agents designed to exert dual functions, the toxicity of synthetic compounds remains a major obstacle 10. Given these limitations, edible natural products, particularly phytochemicals, have sparked interest as safer, multi-targeted anticancer agents 11. An ideal therapeutic strategy would therefore involve identifying a single phytochemical capable of simultaneously targeting both drug resistance and cancer metastasis, while minimizing undesired side effects 6.

Conceivably, since the continuous emergence of drug resistance during cytocidal therapies appears inevitable and may compromise their long-term efficacy in improving cancer patient survival 8, 9, re-sensitizing resistant tumor cells to original drugs with non-cytotoxic natural phytochemicals may serve as a key to better cancer treatments without introducing additional side effects. For instance, vitamin D has been shown to restore cisplatin sensitivity in oral cancer 12, and phytochemicals from *Marsdenia tenacissima* can reverse doxorubicin resistance across various cancer types 13. Multidrug resistance in cancer cells is often driven by membrane-bound efflux pumps, particularly ATP-binding cassette (ABC) transporters such as P-glycoprotein 1 (Pgp1/MDR1), which are overexpressed in various cancers and effectively expel drugs from the plasma membrane 14. Pgp1/MDR1 is therefore an ideal target for re-sensitizing chemo-resistant adherent tumor cells (ATCs). Indeed, several herbal medicines and traditional Chinese medicinal (TCM) compounds have shown potential as non-toxic Pgp1 reversal agents 14. However, few studies have addressed whether such agents can also target CTCs and suppress metastasis.

CTCs adhere to endothelial receptors within the vasculature, thereby initiating metastatic colonization 1. Fibronectin (FN), a multifunctional extracellular glycoprotein, plays a critical role in metastatic processes and exists in two major dimeric species: plasma fibronectin (pFN) and cellular fibronectin (cFN). Although pFN is abundant in the circulation, accumulating evidence indicates that its pro-metastatic activity depends on incorporation into a polymerized pericellular fibronectin (periFN) matrix around CTCs rather than the soluble form itself. PeriFN assembly on surfaces of CTCs is primarily driven by tumor-derived cFN, on which circulating pFN binds through FN-FN self-association to form a stable pericellular matrix 15-17. Recent studies show that periFN assembled on CTCs promotes lung metastasis by engaging the endothelial receptor dipeptidyl peptidase IV (DP4) 15, 16, 18, and disrupting the FN-DP4 interaction markedly impairs lung colonization 17-19, highlighting periFN as a promising anti-metastatic target. Consistently, we have previously demonstrated that soluble pFN neither competes with suspended tumor cell (STC) adhesion to DP4 *in vitro* nor prevents CTC–endothelial interactions *in vivo* despite its high circulating levels 15. Clinically, elevated tumor-associated FN is strongly correlated with metastasis and poor prognosis 20, 21. In light of the fact that cancer cells become highly malignant and metastatic after developing drug resistance 3, 6, cancer cells that are resistant to cytocidal anti-cancer drugs indeed aberrantly express cFN 22, implicating the potential to identify a single phytochemical that dually targets Pgp1-mediated drug resistance and periFN-mediated metastasis through a shared molecular pathway 6. Although our previous studies identified phytochemicals such as pterostilbene and α-mangostin that can dose-dependently inhibit periFN assembly on suspended tumor cells (STCs) and CTC lung colonization 16, 23, it remains unknown whether such compounds or other periFN-suppressing phytochemicals can also differentially re-sensitize drug-resistant tumor cells to chemotherapy.

Here, we demonstrated for the first time that screening several traditional herbal medicines with potential anti-cancer activities can have dual-purpose effects in reversing drug resistance and preventing cancer metastasis. Among them, we identified Mulberroside A (Mul A), the key active component of *Cortex Mori Radices* (CMR) extract, which confers dual anti-cancer activity by targeting the shared upstream ERK/Pgp1 axis, reversing ERK/Pgp1-mediated paclitaxel resistance (TR) in tumor cells and suppressing periFN assembly on STCs. These actions lead to drug re-sensitization and prevention of lung metastasis, respectively. Consistent with expectations, Pgp1 was commonly suppressed by Mul A through the inhibition of ERK, which mediated the resensitization of TR tumor cells to paclitaxel (PTX) and decreased periFN-promoted cancer metastasis by reducing XIAP expression. Collectively, these novel findings may address the urgent need to simultaneously overcome drug resistance and prevent tumor metastasis, thereby alleviating current therapeutic challenges, and highlight Pgp1 as a shared upstream target that can concurrently reverse drug resistance and inhibit FN-driven metastasis.

## Materials and Methods

### Cell lines and reagents

All cell lines used in this study were obtained from commercial sources or collaborators, as detailed in the Supplementary Information. Cells were maintained at 37 °C in a 5% CO_2_ incubator and tested negative for mycoplasma contamination. Culture media for all cell lines, except LLC cells, were supplemented with 10% fetal bovine serum (FBS) (Gibco BRL, USA), L-glutamine (2 mM), and sodium pyruvate (1 mM). For LLC cells, DMEM containing L-glutamine (2 mM) was used, but without sodium pyruvate. Unless otherwise specified, all pharmaceutical compounds and polyclonal antibodies (pAbs) against FN were purchased from Sigma-Aldrich (St. Louis, MO, USA) (see Supplementary Materials and Methods for details).

### Establishment of adaptive PTX-resistance cell lines

LLC P cells were seeded and cultured for two days before being exposed to various concentrations of PTX at a dose-stepwise increment for two days. The persistent but fragile tumor cells were allowed to regrow upon the removal of PTX. Once the cells developed resistance to the initial PTX dose after 3~5 treatment cycles, the tumor cells were subsequently exposed to higher PTX concentrations following the same stepwise adaptation approach. Finally, LLC cells that were incrementally adapted and resistant to the PTX treatment, ranging from 15 to 25 ng/mL, were named LLC TR25 (LLC TR). CNS-1 P cells that were incrementally adapted to the PTX treatment from 15 to 50 ng/mL were named as CNS-1 TR50 (unpublished results). The LLC TR and CNS-1 TR50 cell lines were constantly maintained in culture media supplemented with 25 ng/mL and 50 ng/mL PTX, respectively, to preserve their resistant phenotype.

### Plasmid transfection by electroporation

Plasmid transfection was performed using the Neon^TM^ Transfection System (MPK5000; Invitrogen, Carlsbad, CA, USA) according to the manufacturer’s instructions, as previously described 24.

### Pgp1 silencing in tumor cells

LLC TR cells were infected with lentiviruses carrying either shScr or shPgp1 (shScr: TRCN00000231750; shPgp1: TRCN00000337844, designated as B2; TRCN00000337846, designated as C2; National RNAi Core Facility) and subjected to stable selection with puromycin (12.5 μg/mL) 16.

### Preparation of three types of TCM extracts

Ultrasonic extraction, supercritical CO_2_ Extraction**,** and traditional boiling water stewing techniques were employed to prepare extracts of Cortex Mori, *Antrodia cinnamomea* (*A. cin.*), and Guilu Erxian Jiao (GEJ), respectively (see Supplementary Materials and Methods for details).

### Phytochemical analysis and identification by GC-MS and HPLC

Gas chromatographic (GC) analyses were performed using a SHIMADZU QP-2020 GC-MS system (Kyoto, Japan). High-performance liquid chromatography (HPLC) analyses were conducted with a Hitachi L-2130 Quaternary Pump, L-2200 Autosampler, and L-2455 PDA Detector (see Supplementary Materials and Methods for details).

### Immunoblotting

Total LLC cell lysates were prepared and subjected to SDS-PAGE electrophoresis, electro-transferring, and immunoblotting/Western blotting (IB/WB) 16. Chemiluminescent signals on PVDF membranes were developed using CyECL reagents according to the manufacturer’s instructions. Internal loading controls were visualized by Coomassie Blue staining on the same blotted membranes.

### Immunofluorescence staining

PeriFN assembly 23 and ERK signaling (see Supplementary Materials and Methods for details) were visualized and quantified using anti-FN pAb, anti-pERK1/2, or anti-ERK1/2 monoclonal antibodies (mAbs).

### Pgp1 activity

Cells were cultured for 24 h and treated with verapamil (40 μM) or Mul A (50 μM) for one hour, followed by incubation with Calcein-AM-red (0.05 μM) for 20 min at 37 °C. Fluorescent images were acquired using an Olympus IX71 fluorescence microscope (Shinjuku, Tokyo, Japan). The fluorescence intensity of individual cells in images was calculated using Image J software.

### Annexin V/PI apoptotic assays

Treated LLC and LLC TR cells, either in adherent or suspended statuses, were subjected to apoptosis detection using Annexin V-FAM and PI according to the manufacturer’s instructions. Apoptotic cell populations were quantified by flow cytometry (FACS Calibur, BD Biosciences, CA, USA). The percentages of annexin V^+^PI^-^ (early apoptosis) and annexin V^+^PI^+^ (late apoptosis) cells were calculated.

### Cell viability

LLC and LLC TR tumor cells (2 × 10^4^) were seeded in 24-well plates for 24 h prior to nuclear staining in live cells with cell-permeable Hoechst 33258 dye for 10 mins (5 μg/mL). Fluorescence microscopy was performed every 24 h using UV excitation at 330 nm. Cell viability was calculated using Image J software as: 100% x (1-PTX^Hoechst+^/ Veh^Hoechst+^). Alternatively, for dead cell detection, the cell-impermeable dye PI was added at each of the aforedescribed time points, and fluorescence images were acquired. Dead cell populations were quantified through FACS analysis using the formula: fold-change (FC) = (Hoechst^+^PI^+^ PTX-treated cells/Hoechst^+^PI^+^ untreated cells)-1.

### Animals and *in vivo* metastasis assays

All animal experiments were conducted in accordance with the Guide for the Care and Use of Laboratory Animals at National Cheng Kung University (NCKU) and approved by the Internal Animal Care and Use Committee (IACUC) of NCKU Laboratory Animal Center [protocol code: 106093 (from 1 August 2017 to 31 July 2020) and 109065 (from 1 August 2020 to 31 July 2023)]. Four-week-old male C57BL/6 mice were acquired from and housed in the Animal Center of the NCKU Medical College. Spontaneous and experimental metastasis assays were performed to evaluate the dual anti-cancer functionalities of phytochemicals in tumor-bearing mice. Treatments included intratumoral, intravenous, intraperitoneal, or oral administration of CMR Ext A, Mul A, and/or PTX, depending on the experimental design and grouping (see Supplementary Materials and Methods for details). In accordance with IACUC guidelines, the maximal tumor size permitted was 20 mm in diameter, and this limit was not exceeded in any experimental group. Mice were monitored daily, and animals showing signs of distress or reaching the tumor size limit were humanely euthanized.

### RT-qPCR analysis of Pgp1 mRNA expression

Total RNA from LLC cells was extracted using TRIzol reagent (Invitrogen) according to the manufacturer’s protocol. Reverse transcription was performed using the RevertAid First Strand cDNA Synthesis Kit (Invitrogen). Quantitative PCR was carried out using SYBR Green chemistry on a StepOnePlus 96-well real-time PCR system (Thermo Fisher Scientific). Detailed procedures are provided in the Supplementary Materials and Methods.

### Clinical Data acquisition and bioinformatics analyses

Gene expression profiles from various cancer patients were acquired from publicly available datasets and analyzed using online bioinformatics tools (see Supplementary Materials and Methods for details) to evaluate the prognostic significance of *FN*, *Pgp1*, and *XIAP* gene expressions.

### Statistical analyses

Statistical analyses were performed using GraphPad Prism 8 (GraphPad Software, San Diego, CA, USA) (see Supplementary Materials and Methods for details).

## Results

### PTX resistance requires Pgp1, while suspended-state periFN assembly drives lung metastasis in LLC TR cells

**Figure 1:**
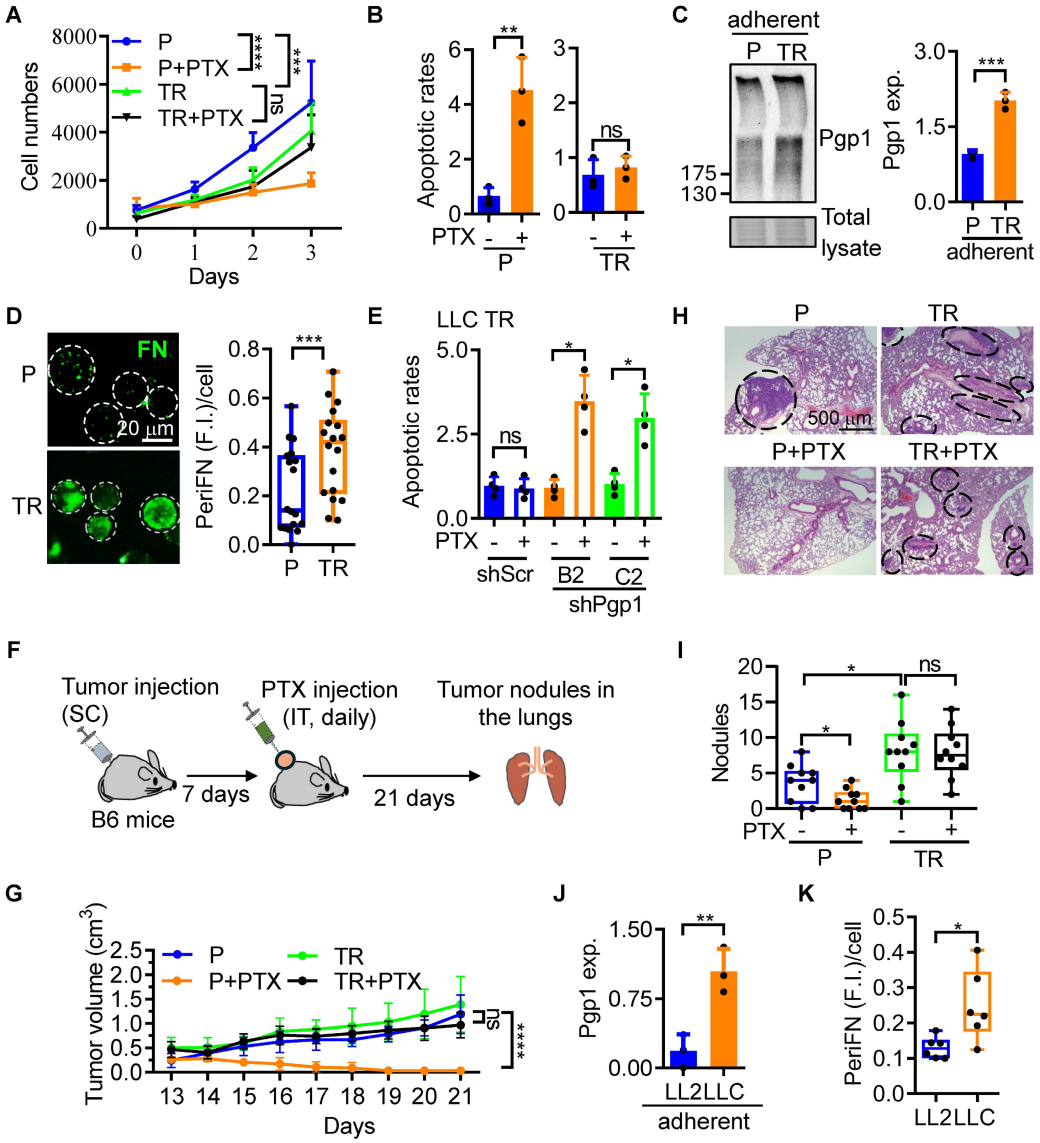
Pgp1 expression is required for the cancerous PTX-resistance that enhances pro-metastatic periFN assembly on STCs. (A) Proliferation rates of LLC P and TR cells treated with Veh or PTX (25 ng/mL). (B) Normalized apoptotic rates of the cells in (A) following 2-day treatment. (C) Immunoblotting (IB) images for Pgp1 expression in LLC P and TR ATCs (left panel) and corresponding quantification (right panel). (D) Representative immunofluorescence (IF) images (left panels; scale bar, 20 μm) and image-based quantification (right panel) of periFN assembly on LLC P or TR STCs. (E) Apoptotic rates of LLC shScr and shPgp1 cells treated with Veh or PTX (25 ng/mL) for 48 h. (F) Experimental schematic for validation of PTX resistance in LLC TR tumor cells in C57BL/6 (B6) mice (related to G-I; n=5 mice). (G) Tumor volumes of B6 mice subcutaneously (SC) bearing LLC P or TR cells upon intratumoral (IT) injections of Veh or PTX (20 mg/kg) from day 7 to day 21 after tumor injection. (H) H&E staining of lung sections showing metastatic tumor nodules (indicated by black circles/ovals) from mice in (F) sacrificed on day 21. Scale bar, 500 μm. (I) Quantification of the nodule numbers in (F). (J) Pgp1 expression in LL2 and LLC ATCs. (K) Image-based quantification of periFN assembly on LL2 and LLC STCs (as shown in Figure S2B). Note: all experiments were repeated at least three times. Error bars show the mean ± SD, *: p < 0.05; **: p < 0.01; ***: p < 0.001; ****: p < 0.0001; and ns: not significant.

To explore drug-resensitizing effects of various TCMs, we first established a PTX-resistant mouse Lewis lung carcinoma (LLC) cell line, termed LLC TR, by progressively exposing parental LLC (LLC P) cells to increasing concentrations of PTX (Figure S1A). While most LLC P cells rounded and died after a two-day treatment with 15 ng/mL PTX, a small population remained adherent (Figure S1A; two left panels). Upon removal of PTX, these surviving cells were allowed to recover and proliferate for 7 days, then re-exposed to 25 ng/mL PTX. After three cycles of exposure-removal of PTX (Figure S1A; three left panels), we generated LLC TR cells (Figure S1A; right panel). We next tested the PTX sensitivities of LLC TR cells and found that LLC TR cells were completely resistant to 25 ng/mL of PTX as represented by the cell proliferation rates (Figure 1A and S1B), apoptotic rates (Figure 1B and S1C), and cell plasma membrane integrity (Figure S1D-G). As expected 22, both Pgp1 expression in LLC TR ATCs (Figure 1C) and periFN assembly on LLC TR STCs (Figure 1D and S1H) were significantly increased. We continued to silence Pgp1 expression in LLC TR cells (Figure S1I). The knockdown restored sensitivity to PTX (Figure 1E), indicating that Pgp1 is necessary for PTX resistance in LLC TR cells. Because LLC TR cells proliferated more slowly than LLC P cells (Figure 1A), we optimized the inoculation conditions to achieve comparable *in vivo* tumor growth. Subcutaneous injection of 1×10⁵ LLC P or 4×10⁵ LLC TR cells into syngeneic mice resulted in similar tumor volumes (Figure S1J-L). Using these numbers, we confirmed that LLC TR tumors were completely resistant to PTX *in vivo*, unlike LLC P tumors (Figure 1F and G). We next assessed lung metastasis. The number of lung tumor nodules was lowest in the PTX-treated LLC P group (P+PTX), likely due to effective primary tumor control (Figure 1H; left lower panel; Figure S1M-O). In contrast, LLC TR cells formed significantly more lung nodules than LLC P cells, regardless of PTX treatment, although their metastatic nodules were smaller (Figure 1I and S1P). To determine whether metastatic potential correlates with innate PTX resistance, we compared highly metastatic LLC P cells to low-metastatic LL2 cells. Pgp1 expression (Figure 1J and S2A) and periFN assembly (Figure 1K; S2B, C) were both elevated in LLC P cells. Congruently, LL2 cells were highly sensitive to 10 ng/mL PTX, which had little effect on LLC cells (Figure S2D-G). These findings suggest that Pgp1 also contributes to innate drug resistance in highly metastatic ATCs and enhanced periFN in STCs. Altogether, Pgp1 overexpression plays a central role in both acquired and innate PTX resistance, and its associated enhancement of periFN assembly under suspension conditions promotes lung metastasis in LLC TR cells.

### TCM drug screening identifies CMR extract A as a dual inhibitor of PTX resistance and cancer metastasis

**Figure 2:**
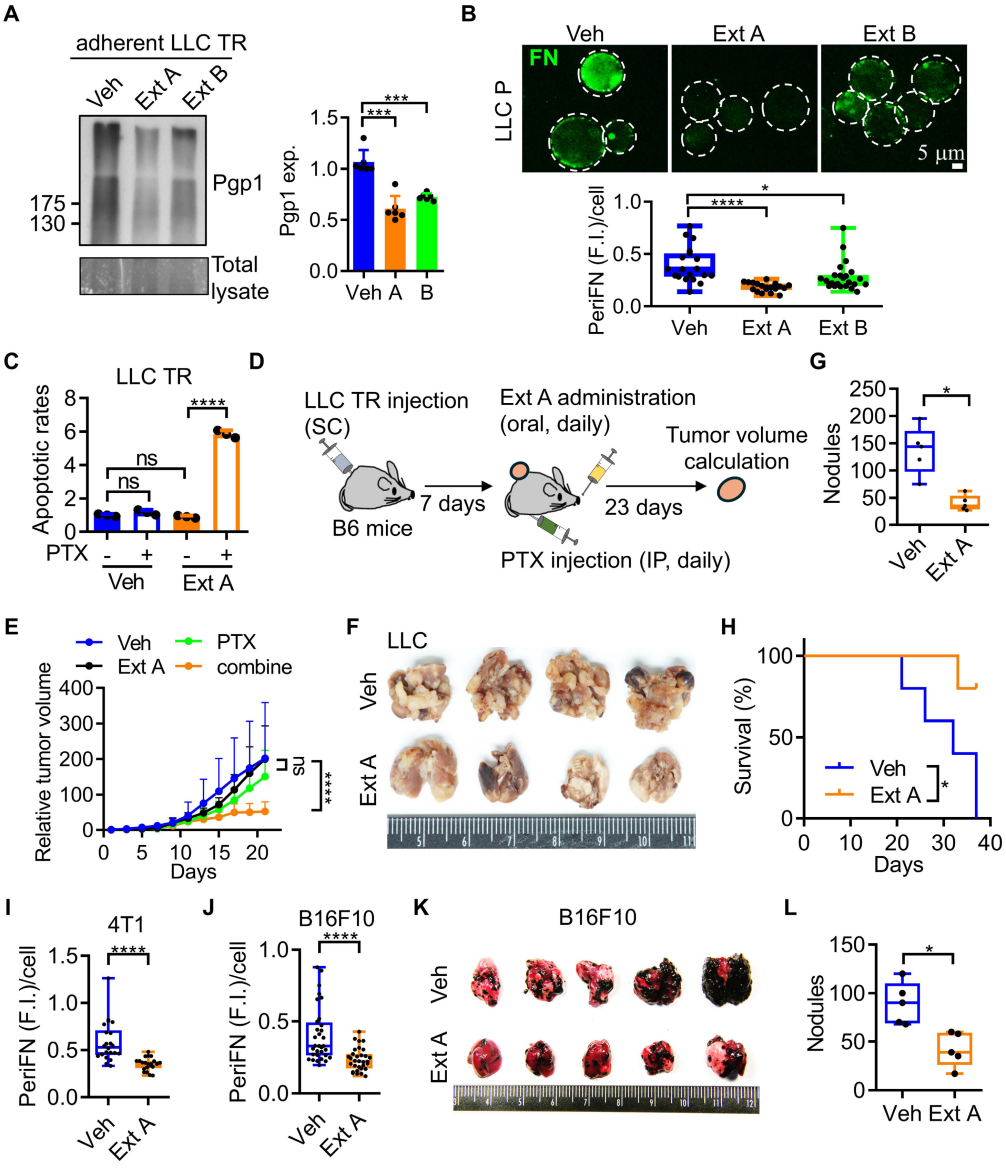
CMR Ext A suppresses Pgp1 and periFN levels, respectively reducing PTX resistance and lung colonization. (A) IB images (left panels) and corresponding quantification (right panel) of Pgp1 expression in LLC TR ATCs treated with Veh, CMR Ext A (750 µg/mL), or Ext B (750 µg/mL). (B) Representative IF images (upper panels; scale bar, 5 μm) and image-based quantification (lower panel) of periFN assembly on LLC P STCs treated with Veh, Ext A (100 µg/mL), or Ext B (100 µg/mL). (C) Normalized apoptotic rates of LLC TR cells after a 48 h-treatment with PTX (25 ng/mL) and/or Veh, or Ext A (750 µg/mL). (D) Experimental schematic for evaluation of the resensitizing effect of Ext A in B6 mice (related to E; n=5 mice). (E) Relative tumor volumes in B6 mice subcutaneously bearing LLC TR cells followed by IP injection of Veh or PTX (20 mg/kg) and/or oral gavage of Veh or Ext A (5 mg/kg) from day 7 to day 23 after tumor implantation. (F-H) Images of mouse lungs (F), quantification of lung tumor nodules (G), and survival curves (H) for B6 mice intravenously injected with LLC P cells pretreated with Veh or Ext A (100 µg/mL) (n=5 mice). (I, J) Image-based quantification of periFN assembly on suspended 4T1 mammary carcinoma cells (I) and B16F10 melanoma cells (J) treated with Veh or Ext A (as shown in Figure S3K and L). (K, L) Images of lungs (K) and quantification of lung tumor nodules (L) from B6 mice intravenously injected with Veh- or Ext A-pretreated B16F10 cells (n=5 mice). All experiments were repeated at least three times. Error bars show the mean ± SD, *: p < 0.05; **: p < 0.01; ***: p < 0.001; ****: p < 0.0001; and ns: not significant.

Since Pgp1 was required for the PTX resistance in LLC cells (Figure 1E) and blocking periFN assembly on STCs can prevent distant metastasis 16, 23, we screened four commonly used anti-cancer TCMs 23, 25-28 for their dual inhibitory functionalities against Pgp1 expression and periFN assembly. Although we have previously found that pterostilbene strongly inhibits periFN assembly on LLC STCs 16, it was excluded from the screening due to its pro-apoptotic effect on LLC ATCs, which conflicted with our goal of identifying compounds that restore PTX sensitivity without directly inducing cell death. Among the four TCMs tested, *Antrodia cinnamomea* (*A. cin*), mangosteen pericarp extracts (MPE), α-mangostin (α-MG, a major component of MPE), and Guilu Erxian Jiao (GEJ), only CMR suppressed Pgp1 expression in LLC TR cells (Figure 2A; S3A and B).

We next assessed their inhibitory effects on periFN assembly on the surfaces of LLC STCs. Except for *A. cin*, all other tested TCMs, including CMR, inhibited periFN assembly (Figure 2B; S3C-E), suggesting that TR-associated periFN regulation may be similarly modulated in both LLC TR and LLC P cells. Of the two CMR extracts tested, extract A (Ext A) demonstrated markedly stronger inhibition of both Pgp1 expression (Figure 2A) and periFN assembly (Figure 2B).

Based on these results, we then selected CMR Ext A as the most promising dual-action candidate. Ext A alone did not exhibit cytotoxicity in LLC TR ATCs but significantly induced cell death when combined with PTX (Figure 2C), indicating excellent resensitization activity. This resensitizing effect was further validated *in vivo* (Figure 2D), where LLC TR tumors treated with the combination of Ext A and PTX showed significantly reduced growth compared to single treatments (Figure 2E; S3F, G). Predictably, Ext A alone significantly prevented lung metastasis (Figure 2F, G; S3H-J) and prolonged survival (Figure 2H) in mice intravenously injected with LLC cells. We also tested Ext A on two highly metastatic murine cancer models, mammary carcinoma 4T1 and melanoma B16F10, and observed significant suppression of periFN assembly on STCs from both lines (Figure 2I, J; S3K, L). In B16F10-bearing mice, Ext A treatment also notably reduced lung metastases (Figure 2K, L; S3H, M, N), suggesting that Ext A contains active phytochemicals capable of concurrently resensitizing PTX-resistant tumor cells and inhibiting metastatic progression.

### Compound analysis identifies Mul A as the principal compound responsible for the dual functionality of CMR Ext A

**Figure 3:**
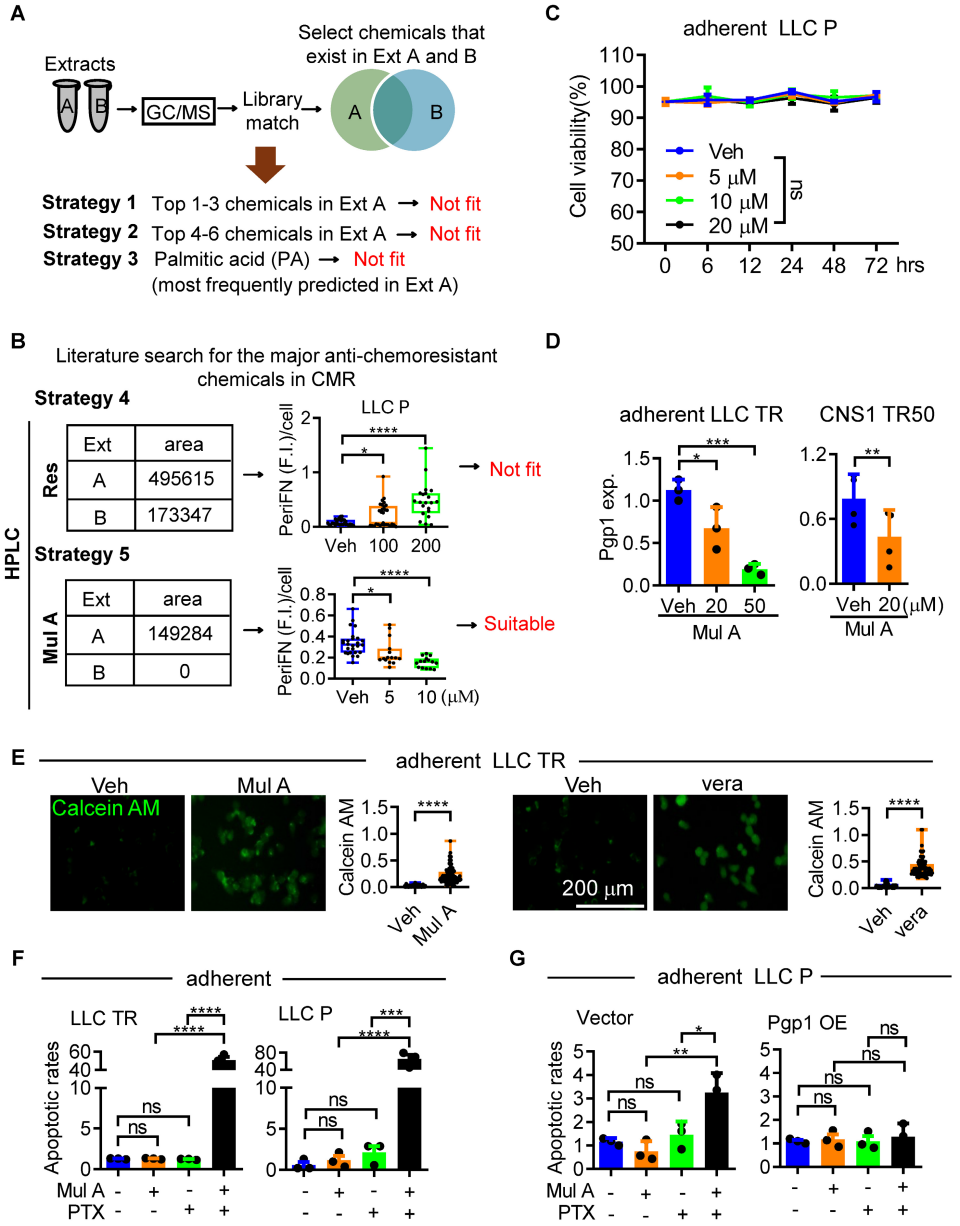
Mulberroside A is identified in CMR Ext A responsible for the dual anti-cancer effects. (A) Workflow and results of GC/MS analyses on CMR Ext A and B for identifying a suitable candidate phytochemical with the dual effects. (B) Workflow and results of HPLC analyses on resveratrol (Res) and mulberroside A (Mul A) for evaluating their potential to mediate the dual effects. (C) Viability of LLC P ATCs after a 72-h treatment with Veh or varying concentrations of Mul A. (D) Quantification of Pgp1 expression in adherent LLC TR (left panel) and CNS1 TR50 (right panel) cells treated with Mul A for 48 h, as shown in the immunoblot (Figure S7F). (E) Representative images (left panels) and quantifications (right panels) of intracellular calcein-AM retention in LLC TR cells treated with Veh or Mul A (20 µM) (left panels) to assess Pgp1 activity. The Pgp1 inhibitor verapamil (vera, 40 µM) served as a positive control (right panels). Scale bar, 200 μm. (F) Apoptotic rates of LLC TR (left panel) or LLC P (right panel) cells in the absence or presence of PTX (25 or 10 ng/mL for LLC TR or P cells, respectively) and/or Mul A (20 μM) for 48 h. (G) Apoptotic rates of Vector (V; left panel) or Pgp1 OE (right panel) LLC TR cells following treatment with PTX (25 ng/mL), Mul A (20 μM), or their combination for 48 h. Note: all experiments were repeated at least three times. Error bars show the mean ± SD, *: p < 0.05; **: p < 0.01; ***: p < 0.001; ****: p < 0.0001; and ns: not significant.

Identifying the active component in CMR Ext A, while excluding unrelated phytochemicals that may cause undesired effects, is crucial for developing CMR-based therapeutic regimens. Initially, we conducted gas chromatography-mass spectrometry (GC-MS) to analyze the chemical profiles of Ext A and Ext B, aiming to identify compounds that were more abundant in Ext A, given its superior inhibitory effects on Pgp1 expression, PTX resistance, periFN assembly, and metastasis (Figure 2 and S3). Using GC-MS reference libraries and databases, we selected the top six most common and abundant lipid species in Ext A to evaluate their potential roles as active compounds (Figure S4A-C; Table S1, 2). However, none were found at higher levels in Ext A than in Ext B, except for oxacycloheptadec-7-en-2-one, which unfortunately failed to suppress Pgp1 expression and periFN assembly (Figure 3A; S4D, E). We alternatively tested palmitic acid as a next-level candidate, which was consistently predicted to be a dominant compound in several GC-MS peaks of Ext A and was less abundant in Ext B (Figure 3A; Table S1, 2). Furfural was chosen as a negative control, as its distribution did not match the expected pattern. We unfortunately found that both palmitic acid and furfural enhanced, rather than inhibited, periFN assembly in LLC STCs (Figure 3A; S5A-C). These findings suggested that key active compounds may be undetectable by GC-MS. We then shifted to an alternative, literature-based approach by screening compounds in CMR extracts reported to enhance PTX sensitivity in chemoresistant cancer cells and tested their abilities to suppress periFN assembly on STCs. We first evaluated resveratrol (Res) as a candidate, as it is one of the major antioxidants in CMR and has been reported to potently sensitize PTX-resistant tumor cells to PTX by targeting Pgp1 29. High Performance Liquid Chromatography (HPLC) confirmed its presence in both extracts, with higher levels in Ext A (Figure S6A). Res unexpectedly increased periFN levels in LLC STCs (Figure 3B and S6C). We then tested Mulberroside A (Mul A), another Pgp1-targeting compound abundantly present in CMR 29. Again, HPLC confirmed its higher concentration in Ext A (Figure S6B). Notably, Mul A significantly and dose-dependently suppressed periFN assembly on LLC STCs without inducing cell death (Figure 3B, C; S6D, E and 7A-E). These results identified Mul A as a strong candidate responsible for the dual functionality observed in Ext A.

### Mul A resensitizes PTX-resistance of LLC cells and solid tumors to PTX via suppressing Pgp1 expression

**Figure 4:**
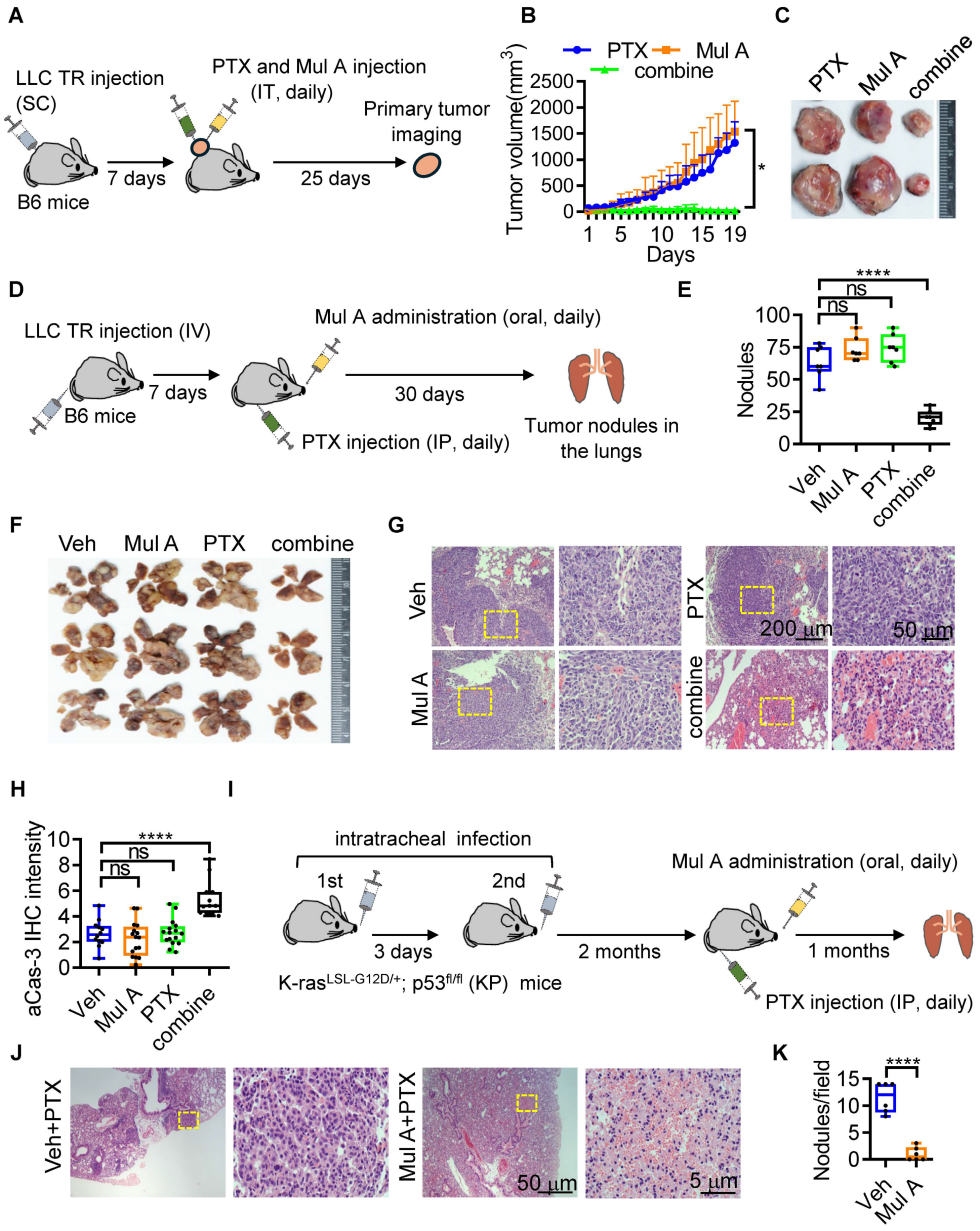
Mul A resensitizes PTX-resistant lung tumors to PTX. (A) Experimental schematic for evaluation of the resensitizing effect of Mul A in primary tumors of B6 mice (related to B, C; n=5 mice). (B) Tumor volumes in B6 mice inoculated with LLC TR cells (SC) and treated daily with PTX (20 mg/kg), Mul A (1.36 mg/kg), or both from day 7 to day 25 (IT). (C) Representative subcutaneous tumors collected at day 30 from mice in (A). (D) Experimental schematic showing the resensitizing effect of Mul A in distant tumors of B6 mice (related to E-H; n=7). (E) Quantification of tumor nodules in the lungs collected at day 30 from mice. (F) Representative mouse lungs showing tumor nodules as quantified in (E). (G) H&E-stained lung sections at low (left panels; scale bar, 200 μm) and high (right panels; scale bar, 50 μm) magnification, depicting tumor nodules from (D). Yellow dashed boxes in the left panels indicate regions enlarged in the high-magnification right panels. (H) Quantification of IHC staining for active caspase-3 (aCas-3) in tumor cells from the lungs is shown in (F). (I) Experimental schematic for evaluation of the resensitizing effect of Mul A in autochthonous tumors of K-ras^LSL-G12D/+^; p53^fl/fl^ (KP) mice (related to J and K; n=5). (J) H&E histopathology of lung tumors in KP mice treated daily for one month with IP PTX (5 mg/kg) and oral gavage of Veh or Mul A (1.36 mg/kg), beginning after the induction and formation of spontaneous lung tumors. Yellow dashed boxes in the left panels indicate regions enlarged in the high-magnification right panels. Scale bars, 50 μm (low magnification) and 5 μm (high magnification). (K) Quantification of tumor nodules per field. Note: all experiments were repeated at least three times. Error bars show the mean ± SD, *: p < 0.05; **: p < 0.01; ***: p < 0.001; ****: p < 0.0001; ns: not significant.

We reconfirmed that Mul A downregulated Pgp1 expression in a dose-dependent manner in LLC TR cells, as well as in another similarly trained PTX-resistant glioblastoma cell line, CNS1-TR50 (unpublished results) (Figure 3D and S7F), leading to reduced Calcein AM-efflux activity (Figure 3E). Although the control Pgp1 inhibitor verapamil (vera) also strongly suppressed both Pgp1 expression and the Calcein AM-efflux activity, it significantly induced cell apoptosis, rendering it unsuitable as a drug candidate (Figure S7G). We next evaluated the resensitization activity of Mul A and found that only the combination of Mul A and PTX, rather than either compound alone, sensitized LLC ATCs to PTX (Figure 3F and S7H). The re-sensitizing effect of Mul A was fully reversed by Pgp1 overexpression (Figure 3G), indicating that Pgp1 downregulation is critical for the Mul A-mediated effect. We then examined the *in vivo* re-sensitizing effect of Mul A. Intratumoral administration of PTX combined with Mul A, but not either agent alone, significantly reduced the sizes of LLC TR tumors (Figure 4A-C; S8A, B). Similarly, daily combinatory treatment with orally administered Mul A and intraperitoneally injected PTX significantly reduced the number of pre-established lung tumor nodules derived from intravenously injected LLC TR or LLC P cells, likely due to restored PTX sensitivity (Figure 4D-F and S8C-G). This therapeutic effect was associated with prolonged survival in treated mice (Figure S8H). These results suggested that Mul A alone can be orally administered and may serve as a dietary nutraceutical to re-sensitize chemoresistant tumors to the original chemotherapeutics. Histologically, LLC tumor nodules in the lungs of mice receiving the combination treatment of Mul A and PTX were markedly reduced (Figure 4G and S8I) and were characterized by apparent cell apoptosis or inflammatory/immune cell infiltration (Figure 4H and S8J). To further test the re-sensitizing effect of Mul A in an autochthonous lung tumor model that more closely mimics human non-small cell lung carcinoma (NSCLC), we employed the K-ras^LSL-G12D/+^; p53^fl/fl^ (KP) mouse model, which is widely used in chemoresistance studies 30. It has been reported that autochthonous lung tumors in KP mice are relatively resistant to intraperitoneal (IP) PTX treatment at 16 mg/kg every other day, compared to K-ras^LSL-G12D/+^ (K) control mice 30. In our study, we observed a similar result, where daily IP administration of PTX at 5 mg/kg alone did not prevent the formation of autochthonous tumor lesions in KP mice. However, co-treatment with orally administered Mul A, which alone had no effect on LLC TR tumor growth in the lungs (Figure 4D-G), significantly induced tumor cell death and suppressed tumor growth (Figure 4I-K; S8K-M). These results further support the ability of Mul A to re-sensitize lung cancer cells exhibiting intrinsic chemoresistance (Figure S2D-G). Taken together, Mul A, an active component of CMR Ext A, not only resensitized PTX-resistant tumors to PTX in both primary and distant sites but also inhibited periFN assembly on CTCs, thereby reducing metastatic dissemination. These findings provided compelling evidence for the dual potential of Mul A to overcome drug resistance and suppress metastasis (Figure S9, 10).

### Mul A prevents lung metastasis of blood-borne LLC cells via impeding the Pgp1-XIAP-periFN signaling axis

**Figure 5:**
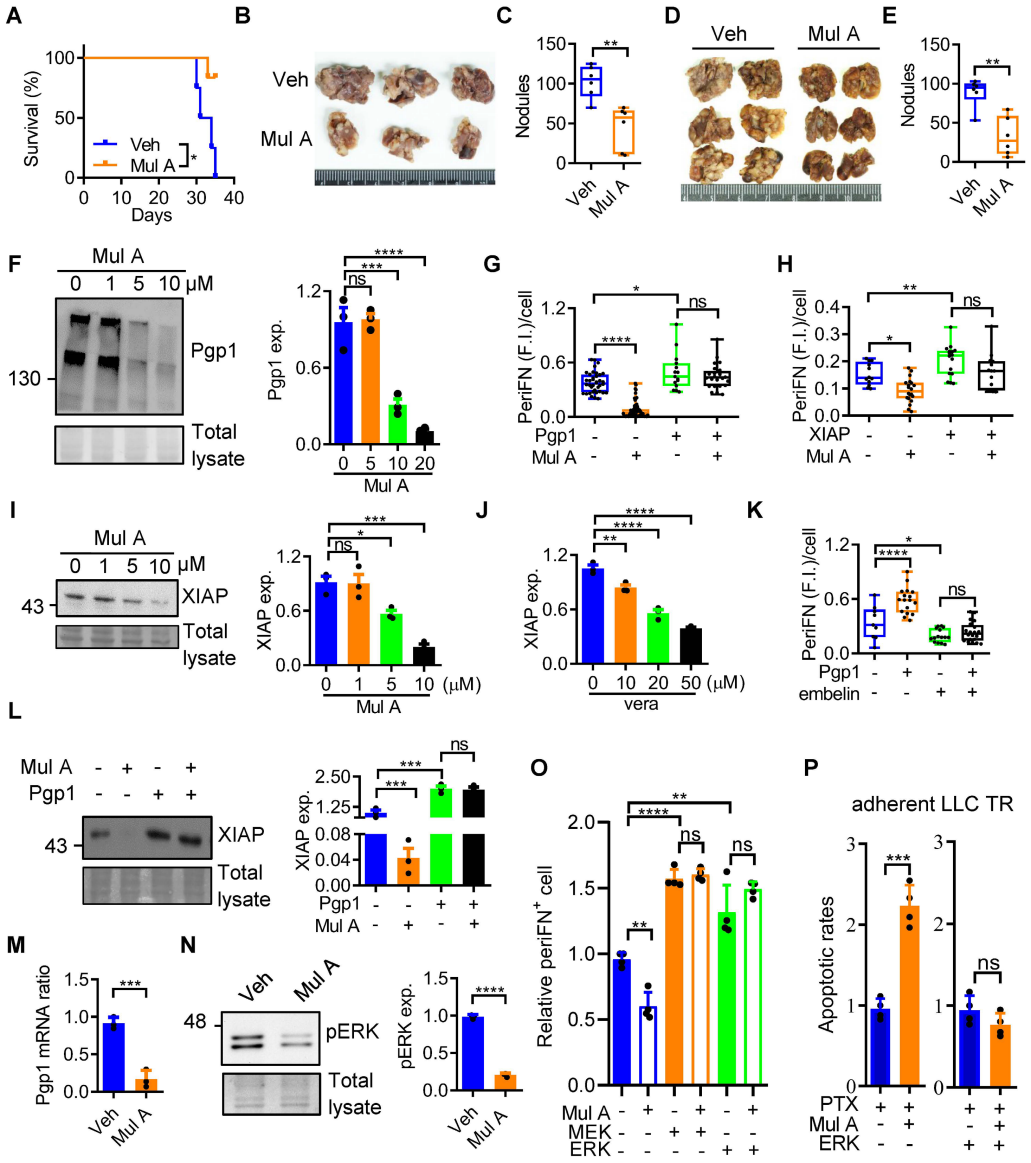
Mul A suppresses ERK/Pgp1/XIAP/periFN signaling to prevent metastasis in STCs and drug resistance in ATCs. (A) Survival rates of B6 mice injected with LLC P cells (IV) pretreated with Veh or Mul A (20 μM). (B, C) Representative images (B) and quantification (C) of lung tumor nodules in mice from (A) (n=6 mice). (D, E) Representative images (D) and quantification of tumor nodules in the lungs (E) taken from B6 mice sacrificed at day 30 post-injection, receiving oral gavage of Mul A (1.36 mg/kg) daily for 3 days (2 h before and 2 days after intravenous inoculation of LLC P cells) (n=6 mice). (F) IB images (left panels) and quantification (right panel) of Pgp1 expression in LLC P STCs treated with various concentrations of Mul A. (G, H) Image-based quantification of periFN assembly on LLC P STCs transfected with V, Pgp1 (G), or XIAP (H), and treated with Veh or Mul A (10 μM) for 2 h (as shown in Figure S11P and 12G). (I) IB images (left panels) and quantification (right panel) of XIAP expression in LLC P STCs treated with various concentrations of Mul A. (J) Quantification of XIAP expression in LLC P STCs treated with various concentrations of verapamil (vera). (K) Image-based quantification of periFN assembly on LLC P STCs transfected with V or Pgp1 and treated with Veh or embelin (15 μM) for 2 h (as shown in Figure S12I). (L) IB images (left panels) and quantification (right panel) of XIAP expression in LLC P STCs transfected with V or Pgp1 and treated with Veh or Mul A (10 μM). (M, N) Quantification of RT-qPCR products for *Pgp1* mRNA (M); representative IB images (N; left panels) and corresponding quantification (N; right panel) of pERK levels in LLC P STCs treated with Veh or Mul A (20 μM) for 2 h. (O) FACS-based quantification of periFN assembly on LLC P STCs transfected with V, ERK or MEK with or without Mul A (50 μM) treatment for 2 h. (P) Apoptotic rates of V (left panel) or ERK OE (right panel) LLC TR ATCs treated with PTX (25 ng/mL), Mul A (20 μM), or their combination for 48 h. Note: all experiments were repeated at least three times. Error bars show the mean ± SD, *: p < 0.05; **: p < 0.01; ***: p < 0.001; ****: p < 0.0001; ns: not significant.

Thanks to the suppressive effect of Mul A on cancerous periFN assembly, we further evaluated whether Mul A suppresses lung metastasis in C57BL/6 mice. To this end, LLC cells pretreated with either Mul A or vehicle were intravenously injected (Figure S11A). Expectedly, mice receiving Mul A-treated LLC cells exhibited significantly prolonged survival (Figure 5A) and fewer lung tumor nodules compared with those receiving vehicle-treated cells (Figure 5B, C; S11B, C). Given the re-sensitizing effect of orally administered Mul A on pre-established tumor nodules in mouse lungs (Figure 4D-G), we asked whether a short-term oral administration could suppress early metastatic colonization. Since intravenously injected tumor cells remain in circulation for approximately two days 19, we administered Mul A orally during this window (Figure S11D). Remarkably, oral Mul A significantly reduced lung tumor nodules in both LLC P- and TR-bearing mice (Figure 5D, E; S11E-J). Next, we investigated the molecular mechanism underlying the Mul A-suppressed periFN assembly on LLC STCs. As the markedly enhanced periFN assembly on LLC TR STCs (Figure 1D), which are resistant to PTX in their adherent state via a Pgp1-dependent mechanism, could be suppressed by Mul A treatment (Figure 3G), we hypothesized that the Mul A-mediated inhibition of periFN assembly in STCs is regulated by Pgp1 activity. To test this, we first confirmed that Mul A reduced Pgp1 expression in LLC P and TR STCs, both of which exhibit high levels of periFN assembly (Figure 5F and S11K). Moreover, both Pgp1 expression and periFN assembly were diminished by either vera or shPgp1 treatment (Figure S11L-N), further confirming Pgp1’s regulatory role in periFN assembly.

Ultimately, our hypothesis was verified by Pgp1 overexpression in LLC STCs, which effectively restored periFN assembly suppressed by Mul A (Figure 5G, S11O and P). Drug-resistant tumor cells often overexpress X-linked inhibitor of apoptosis (XIAP) to evade apoptosis 31. Although XIAP was indeed upregulated in LLC TR ATCs (Figure S12A), treatment with embelin, an XIAP inhibitor, did not contribute to the resensitization of LLC TR cells to PTX (Figure S12B), suggesting XIAP may not directly regulate drug resistance. Next, we asked whether XIAP, potentially acting downstream of Pgp1, is involved in PTX resistance-promoted periFN assembly on LLC P and TR STCs. The positive correlation between the XIAP expression and periFN levels in various metastatic and non-metastatic cancer cell counterparts suggested a potential role for XIAP in triggering periFN assembly on STCs (Figure S12C, D). As expected, embelin dose-dependently suppressed periFN levels in both LLC P and TR STCs (Figure S12E, F). We further demonstrated that the Mul A-suppressed periFN assembly was rescued by overexpressing XIAP (Figure 5H and S12G) that could be downregulated by Mul A or vera treatment (Figure 5I, J and S12H). Notably, embelin also reversed the enhanced periFN assembly caused by Pgp1 overexpression (Figure 5K and S12I). We continued to show that overexpressing Pgp1 restored XIAP expression otherwise inhibited by Mul A treatment (Figure 5L). Altogether, these results suggest that Mul A suppresses periFN assembly on LLC STCs by targeting the Pgp1-XIAP axis. Collectively, pretreatment of tumor cells with Mul A prior to tail vein injection and oral administration of Mul A markedly suppressed lung metastasis by inhibiting periFN assembly on STCs. These findings supported the therapeutic potential of Mul A as a preventive strategy against metastatic progression (Figure S13).

### ERK inactivation mediates the dual functions of Mul A via downregulation of *Pgp1* mRNA levels

**Figure 6:**
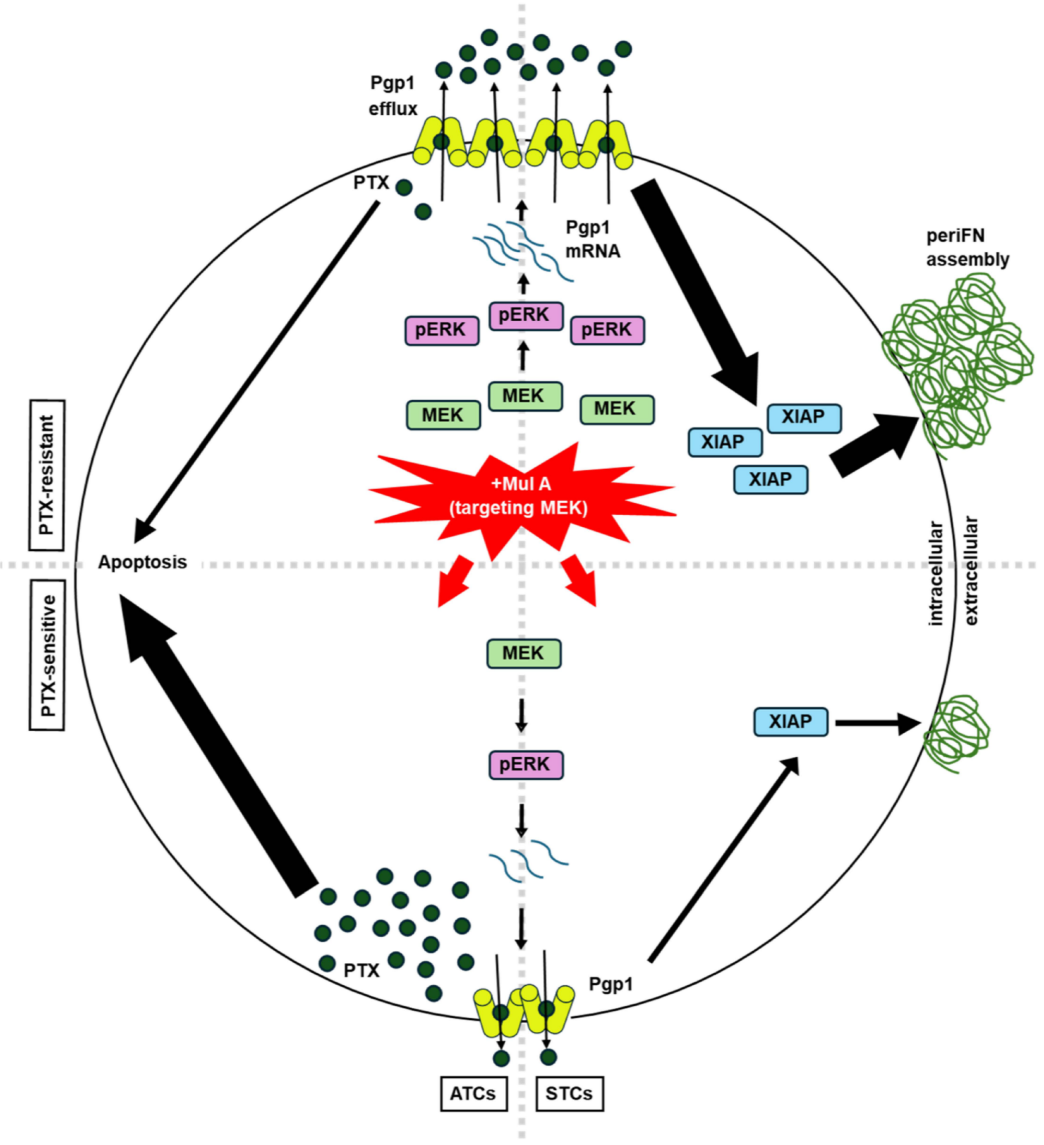
Integrated mechanisms of the dual anti-cancer effects of Mul A. Through an ERK/Pgp1-dependent mechanism, Mul A inhibited XIAP-mediated periFN assembly on STCs (right panel; Figure 5H-O and Figure S11-14), while also resensitizing PTX-resistant ATCs to PTX (left panel; Figure 3F, G and 5P).

To determine whether Mul A-mediated reduction in Pgp1 protein levels was accompanied by changes in mRNA abundance, we first performed RT-qPCR analysis and found that the *Pgp1* mRNA levels were significantly decreased following Mul A treatment (Figure 5M and S14A). Since the reported role of ERK signaling in maintaining Pgp1 expression 32, 33 and in promoting periFN assembly on STCs 17, we next examined the effect of Mul A on ERK activity. In LLC STCs, Mul A treatment decreased both pERK and total ERK protein levels (Figure 5N; S14B-H). Proteasomal, rather than caspase-dependent, degradation contributed to the reduced ERK protein levels, consequently leading to the decreased ERK phosphorylation (Figure S14I, J). Furthermore, Mul A significantly lowered the nuclear-to-cytoplasmic ratio of pERK (Figure S14K, L), indicating a potential decrease in nuclear ERK-mediated signaling responsible for the reduction of *Pgp1* mRNA abundance. In ATCs, we observed an STC-like phenomenon characterized by a reduction in both *Pgp1* mRNA and pERK levels upon Mul A treatment, while total ERK protein levels remained unchanged (Figure S14M-Q). Next, we found that treatment with U0126, a selective ERK inhibitor, reduced *Pgp1* mRNA and protein levels in both STCs and ATCs, and resensitized ATCs to PTX, indicating that ERK activity contributes to *Pgp1* mRNA abundance and PTX resistance (Figure S14R-V). Although U0126 can resensitize PTX-resistant cells to PTX at non-cytotoxic concentrations, being a synthetic compound and exhibiting intrinsic pro-apoptotic activity at higher doses ultimately constrain its translational potential as a therapeutic agent (Figure S14W). Moreover, overexpression of ERK restored the Mul A-suppressed *Pgp1* mRNA levels, as well as downstream periFN assembly and PTX resistance (Figure 5O, P; S14X-Z). Altogether, our results suggested that Mul A is a promising non-cytotoxic therapeutic agent that exerts dual anti-cancer effects by suppressing ERK-dependent Pgp1 expression. Furthermore, through the study of Mul A, we identified the shared upstream molecular mechanism distinctly leading to drug resistance and metastasis (Figure 6).

### Orally administered Mul A differentially resensitizes LLC TR cell-derived tumors to PTX and prevents tumor metastasis in a spontaneous metastasis mouse model

**Figure 7:**
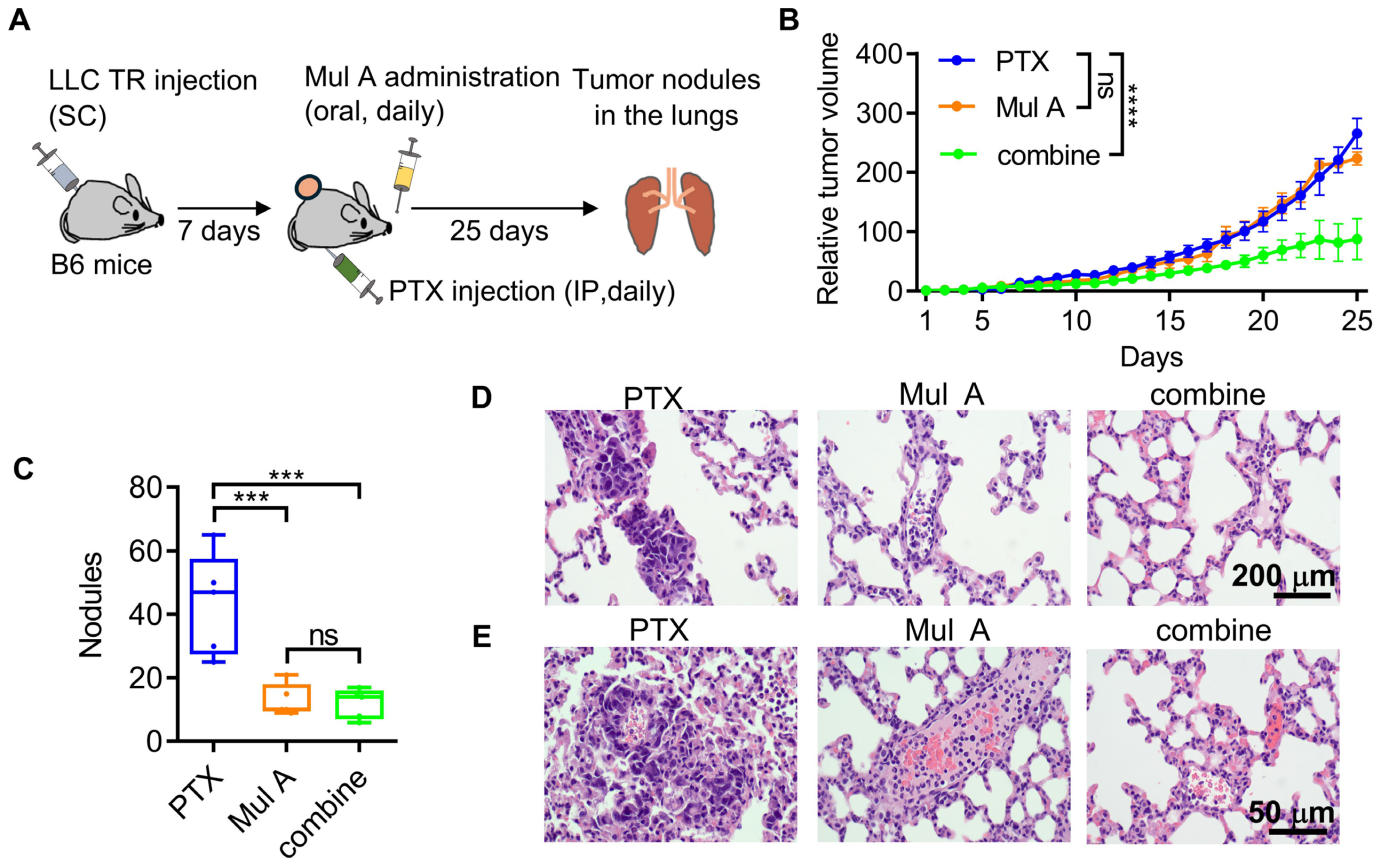
Mul A prophylactically prevents lung metastasis in a subcutaneous tumor model. (A-C) Experimental schematic for evaluation of the dual anti-cancer effects of Mul A in a spontaneous B6 mouse metastasis model (A; related to B-E; n=5 mice). Relative tumor volumes (B) and quantification of tumor nodules in the lungs (C) of B6 mice inoculated with LLC TR cells (SC) and treated daily with oral Mul A and/or IP PTX for 25 days. (D, E) H&E-stained lung sections at low (D; scale bar, 200 μm) and high (E; scale bar, 50 μm) magnification, depicting tumor nodules from (C). Note: all experiments were repeated at least three times. Error bars show the mean ± SD, *: p < 0.05; **: p < 0.01; ***: p < 0.001; ****: p < 0.0001; ns: not significant.

Having unambiguously demonstrated that Mul A, a single CMR-derived phytochemical, exerted the dual anti-cancer effects in a Pgp1-dependent manner (Figure 3-6), we continued to evaluate whether this non-cytotoxic natural compound could be administered orally to simultaneously achieve these effects in a spontaneous metastasis mouse model. B6 mice were subcutaneously inoculated with LLC TR cells and then orally administered Mul A daily, along with IP injections of PTX (Figure 7A). We found that only the combination of Mul A and PTX, but not either agent alone, strongly reduced primary tumor sizes (Figure 7B and S15A). In contrast, lung tumor nodule counts were significantly reduced in mice treated with either Mul A alone or the Mul A/PTX combination (Figure 7C and S15B). Body weights were maintained in mice treated with Mul A alone or in combination with PTX, whereas PTX alone caused weight loss (Figure S15C). Histological analysis revealed well-formed tumor nodules in the lungs of PTX-treated mice, resulting from the colonization of circulating PTX-resistant LLC cells. (Figure 7D, E; left panel). In mice treated with Mul A alone, lung sections showed fewer nodules, with both tumor cells and immune cells visible in the pulmonary vasculature (Figure 7D, E; middle panel). In the Mul A/PTX combination group, even fewer nodules were observed, along with minimal cellular presence in the lung vasculature (Figure 7D, E; right panel). These results suggested that although circulating LLC TR cells were similarly released from subcutaneous tumors in both Mul A- and PTX-treated mice, lung colonization was blocked only in Mul A-treated mice, possibly as a result of reduced periFN assembly and increased immune-mediated clearance. Conceivably, the number of CTCs released from primary tumors was much decreased in mice receiving Mul A and PTX, as Mul A re-sensitized the tumors to PTX.

### The combination of *FN* and *Pgp1* expressions strengthens the prognostic accuracy in early-stage cancer patients and predicts relapse after anticancer therapies

**Figure 8:**
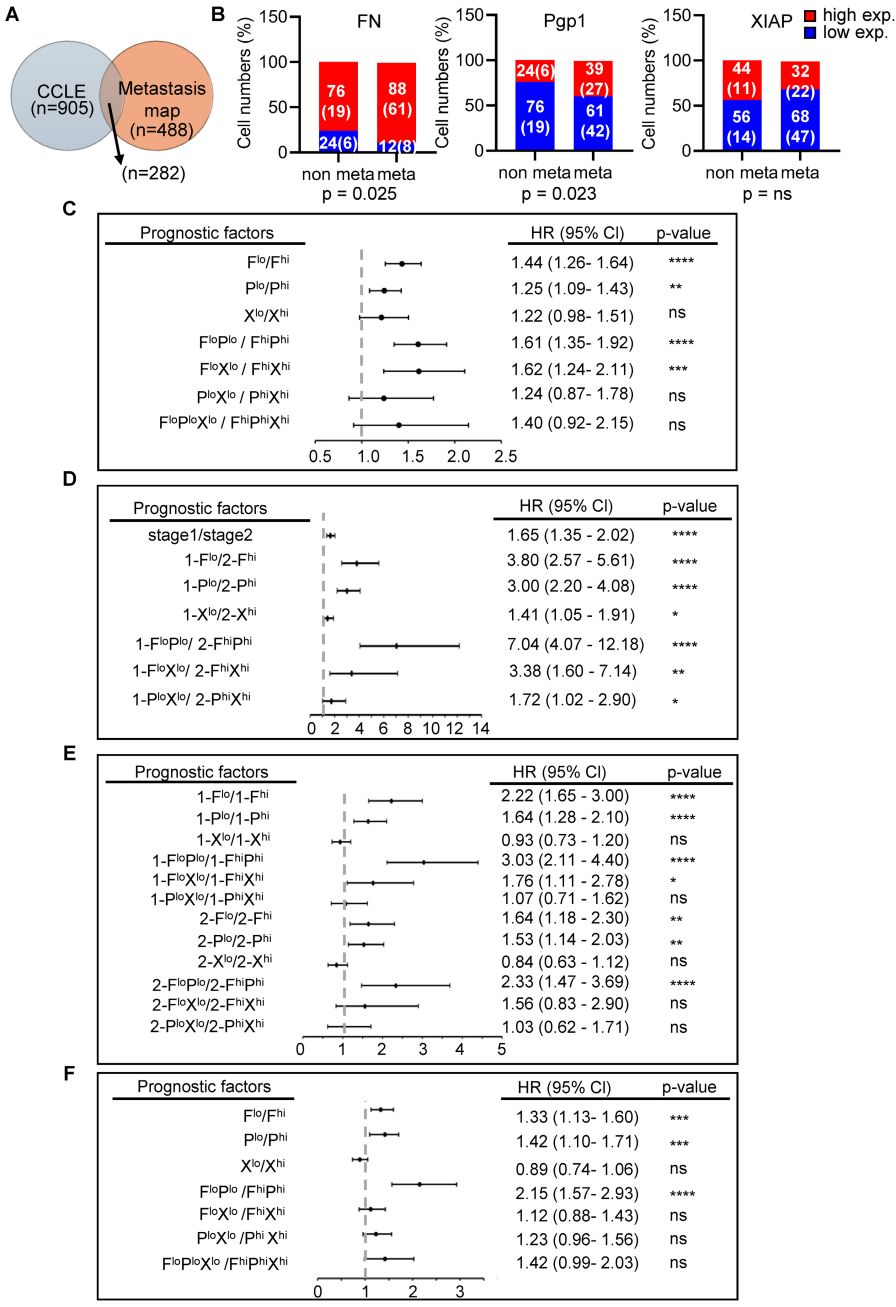
Prognostic values of* FN, Pgp1*, and *XIAP* in cancer patients. (A) Venn diagram showing the numbers of cancer cell types included in both the Cancer Cell Line Encyclopedia (CCLE) and the MetMap databases. (B) Correlations between the expression levels of *FN*, *Pgp1*, or *XIAP* and metastatic potentials of lung cancer cells identified in (A). Note: High and low mRNA expression levels were determined using ROC analysis. Cell lines with metastatic potential > –3 were classified as metastatic (meta) and those with ≤ –3 as non-metastatic (non). Numbers in each bar represent the percentage of cancer cell lines in the non or meta groups, with the number of cancer cell types indicated in parentheses. (C) Hazard ratios (HRs) analyzed individually or in combination, for overall survival (OS) in lung cancer patients based on *FN* (F), *Pgp1* (P), and* XIAP* (X) expression presented as a forest plot. (D) HRs for OS in stage 1 versus stage 2 lung cancer patients, based on F/P/X expression (individually or in combination), to evaluate whether combining gene expression(s) and clinical stage improves early prognostic value. (E) HRs for OS in stage 1 and stage 2 patients, based on F/P/X expression (individually or in combination), to assess whether these genes have greater prognostic value in early-stage (stage 1) disease. (F) HRs for relapse-free survival (RFS) in lung cancer patients based on F/P/X expression (individually or in combination), to evaluate associations with recurrence risk. *Note:* Lung cancer patient data were obtained from the KM-Plotter dataset.

After demonstrating that Mul A exerts dual anti-cancer effects across four cancer types and identifying FN, Pgp1, and XIAP as key regulators, we sought to evaluate their broader prognostic significance. To this end, we cross-referenced human cancer cell lines from the Cancer Cell Line Encyclopedia (CCLE) 34 with the metastasis map dataset 35 (Figure 8A), correlating gene expression levels of *FN*, *Pgp1*, or *XIAP* with metastatic potential. We determined to focus on lung and breast cancer cell lines due to the sufficient sample sizes in both datasets (Figure S16A). In both cancer types, elevated expression of these genes was associated with increased metastatic capability (Figure 8B and S16B). We then performed meta-analysis to evaluate the prognostic value of the three genes in clinical cohorts. High expression of *FN*, *Pgp1*, or *XIAP* each independently correlated with reduced overall survival in lung and breast cancer patients (Figure S16C-H) and could individually serve as a significant risk factor for patient mortality (Figure 8C). Moreover, the combined expression of *FN* and *Pgp1* (FN+Pgp1) outperformed either gene alone in predicting poor prognosis and mortality risk (Figure 8C; S16D, G). Importantly, the prognostic value of FN+Pgp1 for both cancer types was more pronounced in patients at clinical stage I than at stage II (Figure 8D, E; S17B, E), suggesting its relevance for early detection. Moreover, FN+Pgp1 more accurately predicted relapse-free survival following anti-cancer treatments in both lung and breast cancer patients (Figure 8F and S18). In breast cancer patients, combining* FN, Pgp1*, or *XIAP* expression with the triple-negative breast cancer (TNBC) subtype further enhanced the prognostic power of TNBC status alone (Figure S19). Altogether, these meta-analyses clinically validate the involvement of* FN*, *Pgp1*, and* XIAP* in mediating the dual anti-cancer effects of Mul A and support their value as prognostic biomarkers.

## Discussion

Whether chemoresistance is intrinsic or acquired, it often leads to tumor recurrence, spontaneous metastasis, and poor prognosis in cancer patients 6. Consistently, we found that LLC TR cells exhibited significantly elevated Pgp1 expression and enhanced periFN assembly in suspension, which contributed to increased lung metastasis (Figure 1). Similarly, highly metastatic LLC P cells were intrinsically more resistant to PTX and exhibited greater periFN assembly in suspension than the less metastatic LL2 cells, correlating with higher Pgp1 expression (Figure 1). Indeed, Pgp1 14 and cancerous FN, a hallmark of partial EMT, cancer stemness, metastatic CTCs, and endothelial adhesion, are both highly expressed in drug-resistant cancer cells, regardless of whether the resistance is innate or acquired 20, 22. These findings support the notion that elevated Pgp1 and FN expression, alongside increased metastatic potential, are shared features in both innate and acquired chemoresistant tumor cells, suggesting the existence of common regulatory pathways 6.

Although distant metastasis often occurs subsequently after the development of chemoresistance, the two malignant processes have traditionally been studied and targeted separately 6. Thus, we initially designed the strategy to identify a dual-functioning phytochemical based on the hypothesis that Pgp1-induced intrinsic or acquired chemoresistance and cancerous periFN-promoted distant metastasis are independently regulated but could be differentially targeted. Nevertheless, our findings revealed that Mul A, a single phytochemical compound, simultaneously suppressed both phenotypes by targeting their common upstream regulator, Pgp1. Increasing evidence from *in vitro* and *in vivo* studies suggests that these two cellular processes reciprocally reinforce one another during tumor evolution, particularly under autonomous and non-autonomous growth pressures, through mutual crosstalk and shared regulatory mechanisms 4, 6. For instance, in lung cancer, Pgp1 has been reported to promote cisplatin resistance and distant metastasis through the EZH2/Slug axis in a HOXB13-dependent manner 36. HOXB13-promoted cancer metastasis is mediated by the increased NF-κB signalling 37, a well-known inducer of cancerous *FN* transcription 38. Moreover, both EZH2 and Slug have been found to be involved in regulating FN expression 39. Intriguingly, EZH2 is also required for XIAP expression in chronic myeloid leukemia cells under suspension culture 40, thereby supporting the existence of a Pgp1/XIAP/periFN signaling axis driving metastasis, as demonstrated in this study.

The findings of this study suggest that XIAP/periFN-mediated cancer metastasis is driven by Pgp1 upregulation, either as a consequence of PTX resistance or through an independent mechanism. Notably, our results showed that while α-MG upregulated Pgp1, potentially enhancing drug resistance, it concurrently downregulated periFN assembly in LLC STCs (Figure S3), indicating that periFN-driven metastasis can occur independently of chemoresistance. In addition, Res, a chemo-sensitizing phytochemical 41, did not reduce periFN assembly on STCs (Figure 3). Furthermore, Mul A alone was sufficient to directly prevent periFN assembly and lung metastasis of STCs via suppressing the Pgp1-XIAP signalling axis without requiring prior PTX sensitization in adherent cells (Figure 5). Although both Pgp1 and XIAP are often overexpressed in chemoresistant cells 42, no existing study has demonstrated a direct role for XIAP in mediating Pgp1-driven chemoresistance. This is consistent with our clinical meta-analyses in lung and breast cancer patients, showing that while combining *FN* expression as a metastatic risk factor and* Pgp1* expression as a chemo-resistant risk factor provided a superior prognostic index, whereas replacing *Pgp1* with *XIAP* expression as a chemo-resistant risk factor diminished its predictive value (Figure 8). This supports the idea that XIAP is not directly involved in Pgp1-induced chemoresistance. Similar trends were observed in patients at clinical stage I (Figure S17), in TNBC cohorts (Figure S19), and in relapse-free survival analyses (Figure S18).

The proliferation of LLC TR cells in the presence of PTX at a concentration that otherwise induced significant cell death in LLC P cells (Figure 1) was markedly slower than that of LLC P cells during routine culture (Figure 1), reminiscent of the slow-growing characteristics of cancer stem cells (CSCs) 6. Echoing our findings, chemoresistant cells that aberrantly express several CSC markers often exhibit significantly reduced tumor xenograft formation due to their slow proliferation; however, these subcutaneously inoculated chemoresistant cells can spontaneously form distant metastasis 43. Similarly, slow-cycling human persistent melanoma cells resistant to the BRAF inhibitor PLX4720 have been observed to emerge within tumors and become encapsulated in an FN-rich extracellular matrix 44. The ECM-associated property of slow-cycling in chemoresistant cancer cells has been well documented as a feature of cancer stem-like cells 45, 46. In congruence with this notion, the FN organization induced by cancer cells has been reported to suppress cell cycle progression via Rho-associated coiled-coil-containing protein kinase 1 (ROCK1)-generated matrix tension 47. Indeed, we have also revealed in an *in vivo* animal model that downregulation of the FN matrices, induced through the RhoA/stress fiber signaling axis, ameliorates the slow-cycling property of tumor cells 24.

Given that CTCs derived from primary tumor tissues have high heterogeneity within individuals, CTCs endowed with mesenchymal traits and stemness features are critical drivers of distant metastasis 48, 49. In line with this notion, chemoresistant cancer cells in primary tissues can acquire a hybrid EMT phenotype, consequently generating highly metastatic CTCs 48. Importantly, FN is a well-recognized EMT marker closely associated with cancer metastasis 50. PeriFN assembly on the surfaces of CTCs has been reported as a prerequisite for distant metastasis 15, 16, 18. In agreement with this trend, abundant preclinical and clinical evidence demonstrates that FN expression is critically associated with both cancer resistance and metastasis 51. Taken together, these observations underscore the rationale for identifying a single compound capable of differentially targeting Pgp1 and FN, potentially serving as an Achilles’ heel of cancer to simultaneously combat chemoresistance and tumor dissemination.

The identification and analysis of phytochemicals contributing to the various functionalities of TCM extracts using GC-MS has long been recognized as a standard and effective methodology 52. However, the quantities and chemical properties of compounds identified in the two types of CMR extracts via GC-MS did not fulfill the criteria set forth for candidates with dual inhibitory activities (Figure 3). Mul A, which fulfilled these criteria, was absent among the peaks identified and analyzed by GC-MS. A plausible explanation is that its structural complexity and high boiling points render it insufficiently volatile to be desorbed and transported by the gas carrier into the GC detector 53. Alternatively, the stationary phase materials or eluent solvents may have been suboptimal for effectively separating the analytes from unwanted matrices or releasing them from the solid-phase materials 53. To overcome these limitations, combining GC-MS with HPLC analysis may provide a more comprehensive approach for extracting a broader range of phytochemicals 54.

The edible phytochemical Mul A (Oxyresveratrol-3-O-β-D-glucopyranosyl-4′-O-β-D-glucopyranoside) exerting the dual inhibitory effects in this study belongs to 1,2-diphenylethylene-based stilbenes, a small family of polyphenolic secondary glycosylated metabolites derived from oxyresveratrol (trans-3,5,2′,4′ -tetrahydroxy-stilbene) that are distributed among distantly related dietary plant species such as CMR and possess broad biological activities 55, 56. Our results, showing that Mul A restored the sensitivity of LLC TR cells to PTX by targeting Pgp1 and blocking PTX efflux (Figure 3), are in line with reports indicating that downregulation of Pgp1 by CMR extracts or Mul A compromises drug delivery through potential herb-drug interactions 57. Beyond Mul A, accumulating evidence supports the notion that plant-derived polyphenolic compounds, primarily flavonoids and stilbenes such as Res (trans-3,5,4′-trihydroxy-stilbene) and PS (trans-3,5-dimethoxy-4′-hydroxy-stilbene), function as potent inhibitors of ABC transporters, including Pgp1 58. However, we demonstrated that Mul A suppressed XIAP-mediated periFN assembly on STCs (Figure 5), whereas Res instead enhanced periFN assembly (Figure 3), likely due to its multifunctionality, which also targets a downstream molecule of Pgp1 59. Res and PS are unsuitable as chemoresensitizers due to their cytocidal properties 16, 59. The hydroxy group at the R4′ position of the second phenyl ring is shared by cytocidal Res and PS but replaced by an -O-β-D-glucopyranosyl group in non-toxic Mul A 55, implicating that the R4′ hydroxy group may be critical for target binding and induction of apoptosis. On the other hand, modifications of the hydroxy group at the R3 position on the first phenyl ring in periFN-promoting Res with either methyl group or β-D-glucopyranosyl group in periFN-inhibiting PS or Mul A, respectively 55, implicating that alteration of the R3, but not at the R5, hydroxy group, favors inhibition of periFN assembly. These possibilities warrant further investigations to facilitate the discovery of novel and improved anticancer agents. Thus, Mul A should be viewed as a non-cytotoxic adjuvant compound that critically reveals the biological significance of ERK/Pgp1 signaling, rather than merely as a conventional chemotherapeutic agent.

It is well established that mRNA abundance is governed by an interplay of transcriptional and post-transcriptional mechanisms 60-62. Specifically, HSF1 and AP-1, driven by ERK signaling, are well documented to promote *Pgp1* transcription 63-66, supporting a model in which *Pgp1* mRNA and protein levels were reduced by Mul A, possibly by blocking ERK-dependent transcriptional regulation (Figure S14X-Z). Alongside transcriptional control, *Pgp1* is also subject to post-transcriptional regulation 67, 68. For instance, ERK has been reported to modulate mRNA stability and decay via downstream effectors, including RNA-binding proteins and mRNA modification pathways 69-71. Therefore, the ERK-dependent reduction of *Pgp1* mRNA by Mul A may involve both transcriptional and post-transcriptional mechanisms, a possibility that warrants further investigation. Our findings show that ERK inhibition decreases *Pgp1* mRNA levels, accompanied by reduced protein levels (Figure S14R–U). As Pgp1 is functionally implicated in both drug resistance and periFN assembly, these findings provide a mechanistic rationale for how Mul A concurrently reverses chemoresistance and suppresses periFN assembly via ERK-dependent downregulation of Pgp1 (Figure 5O, P). Collectively, our study highlights the therapeutic potential of Mul A as a non-cytotoxic agent targeting a shared upstream pathway linking chemoresistance and metastatic competence.

## Conclusions

In this study, we systematically screened TCM for a phytochemical capable of dually inhibiting Pgp1-mediated drug resistance and periFN-driven metastasis through a shared molecular target. Using GC and HPLC-based chemical profiling, we identified Mul A, the active extract of CMR, as a dual-function candidate. Without inducing intrinsic cytotoxicity, Mul A resensitized PTX-resistant LLC cells to PTX* in vitro* by inhibiting Pgp1 and reduced* in vivo* tumor sizes. Nevertheless, Mul A alone was sufficient to prevent lung metastasis of LLC CTCs. Mechanistic studies uncovered that Mul A targets the ERK-dependent Pgp1 biosynthesis, serving as a shared upstream regulator that concomitantly attenuating chemoresistance and XIAP/periFN-mediated colonization. Importantly, oral administration of Mul A sufficiently achieved these dual effects, revealing its translational potential as a non-cytotoxic cancer therapeutic. In addition, meta-analyses of patient datasets showed that co-elevation of* FN* and *Pgp1* expression was strongly associated with increased risk of relapse and poorer overall survival, particularly in early-stage cancers, highlighting their prognostic values. Together, these findings suggest that Mul A shows promise as a novel non-cytotoxic therapeutic agent and elucidates the shared upstream mechanism linking distinct downstream chemoresistance and metastasis.

## Supplementary Material

Supplementary methods, figures and tables.

## Data Availability

All data supporting the findings of this study are included within the main text and Supplementary information.

## Abbreviations

CTCs: circulating tumor cells
ABC: ATP-binding cassette
Pgp1: P-glycoprotein 1
ATCs: adherent tumor cells
STCs: suspended tumor cells
TCM: traditional Chinese medicinal
FN: Fibronectin
periFN: pericellular FN
DP4: dipeptidyl peptidase IV
Mul A: Mulberroside A
CMR: *Cortex Mori Radices*
PTX: paclitaxel
TR: paclitaxel resistance
FBS: fetal bovine serum
pAbs: polyclonal antibodies
mAbs: monoclonal antibodies
*A. cin.*: Antrodia cinnamomea
GEJ: Guilu Erxian Jiao
MPE: mangosteen pericarp extracts
α-MG: α-mangostin
GC: Gas chromatographic
HPLC: High-performance liquid chromatography
IB/WB: immunoblotting/Western blotting
LLC: Lewis lung carcinoma
Ext A: extract A
vera: verapamil
NSCLC: non-small cell lung carcinoma
KP: K-ras^LSL-G12D/+^;p53^fl/fl^
IP: intraperitoneal
IT: intratumoral
XIAP: X-linked inhibitor of apoptosis
CCLE: Cancer Cell Line Encyclopedia
