# Supplementary material for: P-glycoprotein 1 as a shared target for resensitizing drug-resistant tumor cells and preventing fibronectin-driven metastasis
Source: Theranostics. 2026 Jun 4;16(13):7288–307. doi: 10.7150/thno.131375 (PMC13294981; doi:10.7150/thno.131375)
Supplement: Supplementary file 1 — Supplementary methods, figures and tables. [file thnov16p7288s1.pdf]

Supplementary Materials and Figures

Materials and Methods

Cell Lines

Lewis lung carcinoma (LL2) (ATCC: CRL-1642), MCF7 (ATCC: HTB-22), MDA-MB-231 (ATCC: HTB-26), and B16F10 (ATCC: CRL-6475) cell lines were obtained from the American Type Culture Collection, and CNS-1 (Sigma SCC487) was purchased from Sigma-Aldrich. All cell lines were cultured in Dulbecco's modified Eagle's medium (DMEM; Gibco, Grand Island, NY, USA). The LLC cell line was established from LL2 cells that were injected into C57BL/6 mice via the tail vein and subsequently isolated from pulmonary metastatic nodules. 4T1 and 67NR cell lines, both cultured in DMEM, were received as a kind gift from Dr. Fred R. Miller at Department of Immunology and Microbiology, Wayne State University School of Medicine (Michigan,USA). CL1-5 is a highly invasive subline selected by a Transwell invasion assay from CL1-0 cells [1] was obtained from Dr. Pan-Chyr Yang (Academia Sinica, Taipei, 106, Taiwan). Both CL1-0 and CL1-5 cells were maintained in RPMI-1640 medium. End-over-end (EoE) suspension culture was performed as previously described [2]. All tissue culture plasticware was either purchased from BD Falcon (Franklin Lakes, NJ, USA) or from Wuxi NEST Biotechnology Co., Ltd (Wuxi, Jiangsu, China).

Plasmids and reagents

The ERK and MEK plasmids were kindly provided by Dr. Pai-Sheng Chen, and the XIAP plasmid was obtained from Dr. Ping Lin at National Cheng Kung University College of Medicine (Tainan, Taiwan). The Pgp1 plasmid was generously provided by Dr. Kazumitsu Ueda at the Institute for Integrated Cell-Material Sciences (WPI-iCeMS), Kyoto University (Kyoto, Japan). 4′,6-diamidino-2-phenylindole (DAPI), Hoechst 33258 blue fluorescence dye, Propidium iodide (PI) and pcDNA™3.1 were purchased from Invitrogen (Waltham, MA, USA). Goat anti-rabbit (GαR)-Alexa 488, GαR-Alexa 594, rabbit non-immune IgG, and goat non-immune IgG were obtained from Jackson Immunoresearch Laboratories, Inc. (West Grove, PA, USA). Mulberroside A was purchased from International Laboratory USA (San Francisco, CA, US). Paclitaxol (PTX) was from Selleckchem (Houston, TX, USA). Verapamil and CyECL reagents were obtained from Cyrusbioscience (Taipei, Taiwan), and U0126 was purchased from MedChemExpress (NJ, USA). SYBR green chemistry reagents were from Bio-Genesis (Taipei, Taiwan). MG132 and Z-DEVD-FMK were from TargetMol (Boston, MA, USA). Embelin and monoclonal antibodies (mAbs) against Pgp1 (cat. ab170904) were purchased from Abcam (Cambridge, MA, USA). ERK (mAbs, cat. R22685) and pERK (mAbs, cat. R30257) were from Zenbio (Sichuan, China). Polyclonal antibodies (pAbs) against XIAP and Fibronectin were obtained from Proteintech (Rosemont, USA; cat. 10037-1-Ig) and Sigma-Aldrich (Burlington, MS, US; cat. F3648), respectively. Annexin V-FAM apoptosis detection reagent was purchased from LEADGENE (Tainan, Taiwan). The TAlink mouse/rabbit polymer detection system (TAHC04D) was from BioTnA (Kaohsiung, Taiwan). Oxacycloheptadec-7-en-2-one was purchased from Angene International Limited (Nanjing, China). Immobilon-P Poly vinylidene fluoride (PVDF) Membrane (IPVH00010) was from Sigma-Aldrich/Merck Millipore (Darmstadt, Germany). Microscope cover glasses (12 mm) were obtained from Glaswarenfavrik Karl Hecht GmbH and Co. (Sondheim, Germany).

Cortex Mori Radicis extracts preparation: ultrasonic extraction (USE)

Dried Cortex Mori Radicis (CMR), purchased from Hunan Province (China), was milled to pass through a 40-mesh sieve. The resulting powder was mixed with solvents at a ratio of 1:10 (w/w),

using 50% ethanol for extract A (Ext A) and water for extract B (Ext B). Ultrasonic extraction was performed at a working frequency of 40 kHz. For Ext A, homogenization was carried out at 12,000 rpm for 1 h at 50 °C; for Ext B, the process was conducted for 1 h at 60 °C. The extracts were further subjected to suction filtration, and the solvents were removed using a rotary evaporator, followed by lyophilization. For subsequent use, the extract powders were either stored at -20 °C or dissolved in 95% ethanol, and working solutions were subsequently prepared by dilution in DMSO.

Antrodia cinnamomea (*A. cin.*) preparation: supercritical CO_2 Extraction

Lyophilized Antrodia cinnamomea (*A. cin*) was milled and filtered through a 20-mesh sieve. To obtain the extracts, 100 g of *A. cin* powder was subjected to supercritical CO_2 extraction for 2 h at 45 °C, under a CO_2 pressure of 3,500 psi, and a flow rate of 10 mL/min, using ethanol as a cosolvent. The solvent was subsequently removed using a rotary evaporator, and dextrin was added as a binder to the fluid extract at a ratio of 1:5 (w/w). The products were lyophilized and stored at -20 °C or dissolved in Milli-Q water (MQ) for further use.

Guilu Erxian Jiao (GEJ) preparation

GEJ, traditionally prepared by simmering tortoise shell and deer antler syrup in slightly boiling water for 7 days at a 1:1 ratio, was purchased from a Traditional Chinese Medicine Pharmacy at Shanhua District, Tainan, Taiwan. To ensure the quality of the herbal product, GEJ were lyophilized, milled, and passed through a sieve to obtain GEJ powder. The powder was dissolved in water and mixed with dextrin at a ratio of 1:3 (w/w) before being lyophilized again and stored at -20 °C. Working solutions were prepared by dissolving the powder in MQ prior to use.

GC–MS analysis

Gas chromatographic analyses were performed using a SHIMADZU QP-2020 GC-MS (Kyoto, Japan). An Agilent 122-5532 DB-5 ms (0.25 mm × 30 m × 0.25 μm) column coated with (5%-Phenyl)-methylpolysiloxane was used to separate CMR extracts. Phytochemical compounds were injected in split mode with helium as carrier gas (pressure 37.1 kPa; column flow rate 0.8 mL/min). The GC oven temperature was programmed from 50 °C (no initial hold) to 270 °C at a rate of 6 °C/min and held at 270 °C for 23 min. The injector and GC/MS interface temperatures were set at 250 °C. The mass spectrometer was operated in electron-impact mode with an ionization voltage of 1.00 kV and a scan interval of 0.50 s. Compound identification was performed by comparing MS spectra with those in the NIST05.lib, NIST05s.lib, and FFNSC 1.2.lib spectral libraries.

HPLC analysis

The HPLC system consisted of a Hitachi L-2130 Quaternary Pump, Hitachi L-2200 Autosampler, and Hitachi L-2455 PDA Detector. For sample preparation, 5 mL acetonitrile and 3 mL n-hexane were added to 0.5 g of CMR extracts, then mixed by ultrasonication (Ultrasonic Delta DC600H, Tainan, Taiwan), followed by centrifugation (Eppendorf 5415 D, Germany) at 4000 rpm for 10 mins. The upper acetonitrile layer was discarded to remove oil residues, and 3 mL of *n*-hexane was added and extracted three times. The samples were filtered through a 0.45 μm pore size filter before separation on an ODS column (250mm x 4.6 mm, 5 μm particle size). A mixture of acetonitrile and 0.2% phosphoric acid (3:7, v/v, pH 2.5) was used as the mobile phase at a flow rate of 1 mL/min. The injection volume was 20 μL, and chromatograms were monitored at O.D. 303 nm. Quantification was performed using an external standard method. Res or Mul A standards were

prepared by dissolving 5 mg of each compound in 15 mL of methanol and diluting to 20, 10, or 5 μg/mL. The calibration curves were established by plotting peak areas against standard concentrations. The concentrations of the compounds were calculated from peak areas according to calibration curves by using the following formula: X = (As × c × V × 100) / (Ast × W × 1000 × F) [X: Weight of compound in each sample (g/100g); As: Area of the sample; Ast: Area of the standard; c: Concentration of the standard (mg/mL); W: Weight of the sample (g); V: Volume of the sample (mL); F: dilution ratio factor].

Spontaneous tumor metastasis models

C57BL/6 male mice (five per cage) were housed at 24 ± 2 °C with 50 ± 10% relative humidity under a 12 h light/12 h dark cycle. For the tumor injection in primary tissues, mice were subcutaneously inoculated with LLC P or LLC TR cells on day 0. Starting on day 7, daily intratumoral injections of PTX (20 mg/kg) and/or Mul A (1.36 mg/kg) were administered for 30 consecutive days until euthanasia. Subcutaneous tumor volumes were measured, and tumor nodules in the lungs were analyzed by photography, H&E staining, histological microscopy, and quantification. For lung tumor nodule size quantification, nodules were classified as large（> $0.3 mm^2$） or small (< $0.04 mm^2$) based on cross-sectional area (length × width).

Experimental tumor metastasis models

For experimental lung metastasis, 5×10^5 LLC or B16F10 cells were pretreated with a proper concentration of Veh, CMR Ext A, or Mul A before intravenous inoculation into male C57BL/6 mice (5 mice per group), as previously described [3]. After 30 days, mice were sacrificed by CO_2 euthanasia, and mouse lungs were collected for either fresh photography or formalin fixation, quantification, and H&E histological staining.

Evaluation of the dual therapeutic effects of orally administered Mul A on primary and metastatic tumor growth

To assess the reversal of growth in PTX-resistant tumor cells subcutaneously inoculated in primary tissues or intravenously injected and established in lung tissues, 4-8-week-old C57BL/6 male mice pre-bearing LLC-TR cells in subcutis or lung tissues were treated as follows. Starting on day 7 post-tumor inoculation, mice received daily PTX (20 mg/kg) via intratumoral or intraperitoneal injection and either Veh, CMR Ext A（5 mg/kg), or 1.36 mg/kg Mul A (1.36 mg/kg) administered intratumorally or orally once daily till the end of the experiments. Primary tumor volumes were measured, and tumor nodules in the lungs were photographed and quantified. To evaluate the preventive effects of Mul A alone on lung colonization by LLC P and LLC TR cells, mice were orally administered Veh or Mul A (1.36 mg/kg) 2 h before tail vein injection and on days 1 and 2 post-injection, for a total of three administrations. Lungs from sacrificed mice were photographed, and tumor nodules were counted and subjected to H&E staining for histopathological analysis.

Evaluation of PTX-resensitizing effects on spontaneous lung tumor growth in a transgenic mouse model

K-ras$^{LSL-G12D/+}$; p53$^{fl/fl}$ (KP) male mice were induced by intratracheal infection of Cre-adenovirus 0.2 μL for twice at three-day intervals [4]. Eight weeks post-induction, the mice were used to determine the PTX dosage (5 mg/kg) to which spontaneous lung cancer cells exhibited resistance. Next, lung cancer-bearing KP mice received daily oral administration of Veh or Mul A (1.36 mg/kg) combined with daily intraperitoneal injections of PTX (5 mg/kg). After 30 days of

treatment, mice were euthanized via CO_2 inhalation. Lungs were collected for formalin fixation and H&E histological staining (4 mice per group). For lung tumor nodule size quantification, nodules were classified as large (> 315 μm^2) or small (< 168 μm^2) based on cross-sectional area (length × width).

Mouse genotyping and real-time PCR

Genomic DNAs were extracted from mouse tail tissue (~1 cm) using Genomic DNA Mini Kit (Tissue) (Geneaid Biotech Ltd, New Taipei City, Taiwan) according to the manufacturer's instructions, followed by PCR amplification. The primer sequences are listed in the table:

	Name	Sequence	Product size
Kras	y116	5'-TCC GAA TTC AGT GAC TAC AGA TG-3'	y117/y116, 327bp (Lox)
	y117	5'-CTA GCC ACC ATG GCT TGA GT-3'	y116/y118, 450bp (WT)
	y118	5'-ATG TCT TTC CCC AGC ACA GT-3'	
p53	T008	5'-CAC AAA AAA CAG CTT AAA CCC AG-3'	T008/T009, 370bp (Lox)
	T009	5'-AGC GCG TAG GAG GCA GAG AC-3'	T008/T009, 289bp (WT)

Each PCR reaction (20 μL) contained 0.2 mM dNTP mixture, 1 μM of each of the primer sets, 1-unit Taq DNA polymerase, 20 mM Tris-HCl, and 50 mM KCl (pH 8.4). The PCR program consisted of an initial denaturation at 95°C for 5 min followed by 35 cycles of 95°C for 15 s, 61°C for 15 s, and 72°C for 15 s with a final extension step at 72°C for 10 min.

RT-qPCR analysis of Pgp1 mRNA expression

Total RNA was extracted from 1x10^6 LLC cells using TRIzol reagent (Invitrogen) according to the manufacturer's protocol. Reverse transcription was performed using 5 μg of RNA and the RevertAid First Strand cDNA Synthesis Kit (Invitrogen) with an oligo(dT) primer. A parallel reaction without reverse transcriptase served as a negative control. Quantitative PCR was performed using SYBR Green chemistry on a StepOnePlus 96-well real-time PCR system (Thermo Fisher Scientific) with Pgp1 or β-actin primers. The RT-qPCR cycling conditions were: 95°C for 5 min followed by 40 cycles of 95°C for 30 s, 58°C for 30 s, and 72°C for 30 s, and a final extension at 72°C for 10 min. PCR products were analyzed by electrophoresis on a 1.6% agarose gel.

Name		Sequence	Product size
Pgp1	Forward	5'-GCG AGT CCG ATA CAT GGT TT-3'	390
	Reverse	5'-AAC TTC TGC TCC CGA GTC AA-3'	
β-actin	Forward	5'-ACT GCC GCA TCC TCT TCC TC-3'	137
	Reverse	5'-TGC CAC AGG ATT CCA TAC CC-3'	

Immunofluorescence staining of intracellular pERK and ERK

LLC STCs treated with Veh or Mul A were fixed in 4% paraformaldehyde for 20 mins at room temperature (RT). Cells were then permeabilized with 0.2% Triton X-100 for 10 mins at RT, followed by blocking with preimmune goat-IgG antibody for 30 mins at 37°C. Immunostaining was performed using anti-pERK1/2 mAb or anti-ERK1/2 mAb to visualize and quantify pERK and total ERK expression, respectively. The nuclear-to-cytosolic fluorescence intensity ratio was quantified using a multi-photon confocal laser scanning microscope (MPE) equipped with a 60x oil immersion objective, and fluorescence intensities were analyzed with ImageJ software.

Evaluation of ERK protein expression in LLC STCs

LLC STCs were incubated EoE as previously described [2, 3]. Briefly, 1×10^6 cells were resuspended in 300 μL DMEM containing 0.5% BSA and treated with Mul A, MG132, or Z-DEVD-FMK, alone or in combination, for 30 min at 37°C. FBS was then added to a final concentration of 20%, and cells were incubated EoE for an additional 2 h at 37°C. Cells were washed with PBS, lysed in RIPA buffer for 1 h at 4°C and analyzed by IB.

Bioinformatics analysis of cancer patients' gene expression datasets

Unless otherwise specified, all datasets were obtained from cBioPortal [5-7]. mRNA expression data for human cancer cell lines were retrieved from the Cancer Cell Line Encyclopedia (CCLE) and classified as non-metastatic or metastatic based on the criteria defined in the MetMap 500 database [8]. Information on *FN*, *Pgp1*, and *XIAP* gene expression levels, along with the clinical records of lung cancer patients, was downloaded from the KM-Plotter database (n = 2,166 patients; Affymetrix IDs 210495_x_at for FN, 212960_at for Pgp1, and 206536_s_at for XIAP) [9]. Relapse-free survival (RFS) for lung cancer was retrieved from the TCGA pan-cancer radiotherapy dataset, while the METABRIC dataset was used to analyze breast cancer patients [10-12].

Statistical analyses

Data comparisons were performed using Student's t-test (for paired data), one-way ANOVA (for comparisons among two or more independent groups), or two-way ANOVA (to determine variance and test differences in the effects of independent variables on a dependent variable such as tumor cell proliferation). All experiments were conducted independently in biological triplicates, and results are shown as mean ± S.D. Statistical differences were represented by p values: $p < 0.05$ (*), $p < 0.01$ (**), $p < 0.001$ (***), $p < 0.0001$ (****), with lower p values representing higher levels of significance. Unless otherwise indicated, all experiments were repeated at least three times. Error bars represent mean ± SD.

Reference

1. Chu YW, Yang PC, Yang SC, Shyu YC, Hendrix MJ, Wu R, et al. Selection of invasive and metastatic subpopulations from a human lung adenocarcinoma cell line. Am J Respir Cell Mol Biol. 1997;17(3):353-60.

2. Wang YJ, Lin JF, Cheng LH, Chang WT, Kao YH, Chang MM, et al. Pterostilbene prevents AKT-ERK axis-mediated polymerization of surface fibronectin on suspended lung cancer cells independently of apoptosis and suppresses metastasis. J Hematol Oncol. 2017;10(1):72.

3. Huang LT, Kuo CH, Tseng L, Li YS, Cheng LH, Cheng CY, et al. Alpha-Mangostin Reduces Pericellular Fibronectin on Suspended Tumor Cells and Therapeutically, but Not Prophylactically, Suppresses Distant Metastasis. Life (Basel). 2022;12(9).

4. DuPage M, Dooley AL, Jacks T. Conditional mouse lung cancer models using adenoviral or lentiviral delivery of Cre recombinase. Nat Protoc. 2009;4(7):1064-72.

5. Cerami E, Gao J, Dogrusoz U, Gross BE, Sumer SO, Aksoy BA, et al. The cBio cancer genomics portal: an open platform for exploring multidimensional cancer genomics data. Cancer Discov. 2012;2(5):401-4.

6. Gao J, Aksoy BA, Dogrusoz U, Dresdner G, Gross B, Sumer SO, et al. Integrative analysis of complex cancer genomics and clinical profiles using the cBioPortal. Sci Signal. 2013;6(269):pl1.

7. de Bruijn I, Kundra R, Mastrogiacomo B, Tran TN, Sikina L, Mazor T, et al. Analysis and Visualization of Longitudinal Genomic and Clinical Data from the AACR Project GENIE Biopharma Collaborative in cBioPortal. Cancer Res. 2023;83(23):3861-7.

8. Jin X, Demere Z, Nair K, Ali A, Ferraro GB, Natoli T, et al. A metastasis map of human cancer cell lines. Nature. 2020;588(7837):331-6.

9. Győrffy B. Transcriptome-level discovery of survival-associated biomarkers and therapy targets in non-small-cell lung cancer. Br J Pharmacol. 2024;181(3):362-74.

10. Curtis C, Shah SP, Chin SF, Turashvili G, Rueda OM, Dunning MJ, et al. The genomic and transcriptomic architecture of 2,000 breast tumours reveals novel subgroups. Nature. 2012;486(7403):346-52.

11. Rueda OM, Sammut SJ, Seoane JA, Chin SF, Caswell-Jin JL, Callari M, et al. Dynamics of breast-cancer relapse reveal late-recurring ER-positive genomic subgroups. Nature. 2019;567(7748):399-404.

12. Pereira B, Chin SF, Rueda OM, Vollan HK, Provenzano E, Bardwell HA, et al. The somatic mutation profiles of 2,433 breast cancers refines their genomic and transcriptomic landscapes. Nat Commun. 2016;7:11479.

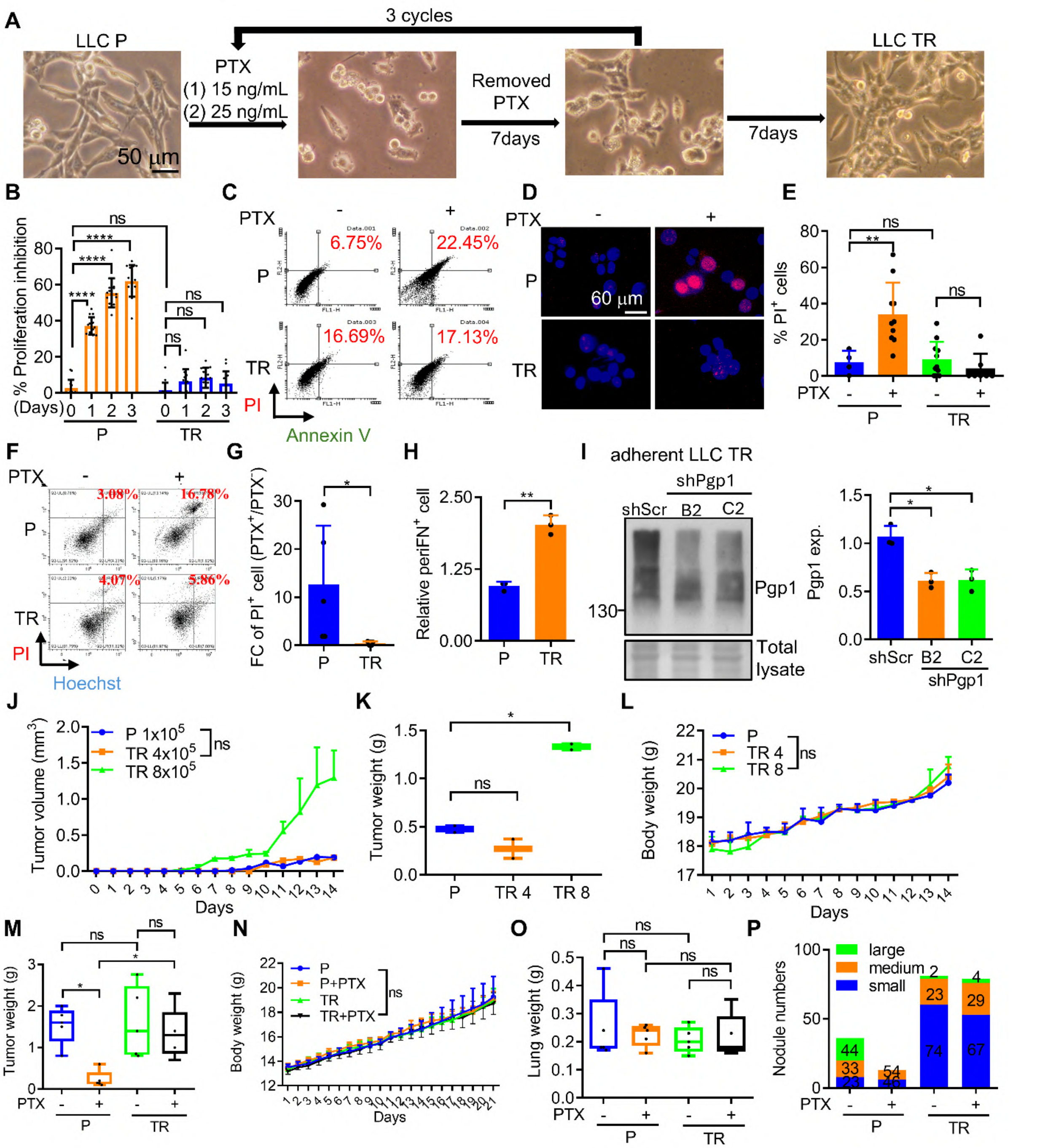

Figure. S1.

Adaptation to PTX treatment leads to chemoresistance, Pgp1 upregulation, and elevated periFN assembly in slow-growing tumor cells. (A) Schematic illustration for the adaptation of LLC P cells to PTX and the establishment of LLC TR cells. Scale bar, 50 μm. (B) The percentage of proliferation inhibition [calculated using the formula: 100% x (1-PTX$^{Hoechst+}$/ Veh$^{Hoechst+}$)] of cells treated with Veh or PTX (25 ng/mL), assessed by Hoechst staining. (C) Dot-plots from fluorescence-activated cell sorting (FACS) analysis corresponding to Figure 1B. Red numbers indicate the total percentages of annexin V$^+$PI$^-$ and annexin V$^+$PI$^+$ cells. (D, E) Representative

images of PI and Hoechst (D) and quantification (E) for dead LLC P and TR cells after a 48-h PTX (25 ng/mL) treatment. Scale bar, 60 μm. (F) Dot-plots from FACS analysis corresponding to Figure S1D. Red numbers indicate the percentage of $Hoechst^{+}PI^{+}$ cells. (G) Quantification of dead cells in (F), calculated using the formula: ($Hoechst^{+}PI^{+\ PTX\text{-}treated\ cells}$/$Hoechst^{+}PI^{+\ untreated\ cells}$)-1. (FC: fold-change) (H) Quantification of FACS analysis shown in Figure 1D. (I) Pgp1 expression levels in LLC TR shScr or shPgp1 (B2 or C2) cells. (J-L) Tumor volumes (J), tumor weights (K), and body weights (L) of C57BL/6 (B6) mice subcutaneously inoculated with various numbers of LLC P or TR cells. (M-O) Tumor weights (M) of subcutaneous xenografts, as well as body (N) and lung (O) weights of B6 mice, measured at the study endpoint (related to Figure 1F). (P) The sums and percentages of small (blue), medium (orange), and large (green) tumor nodules in each group in (Figure 1I). Note: all experiments were repeated at least three times. Error bars show the mean ± SD.

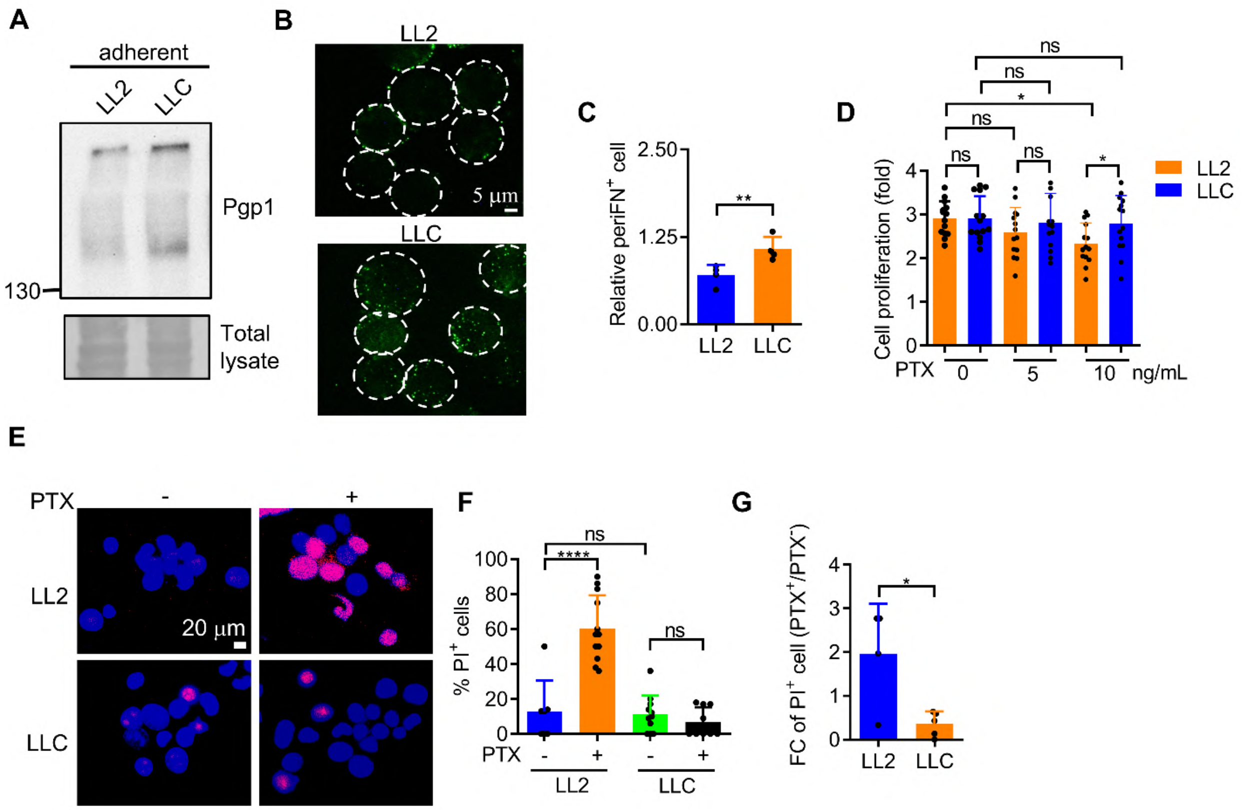

Figure. S2.

Enhanced Pgp1 expression and periFN assembly characterize tumor cells with innate PTX resistance. (A) Representative IB image corresponding to Figure 1J. (B, C) Representative immunofluorescence (IF) images (B) and quantification of FACS analysis (C) for the periFN assembly on suspended LL2 or LLC P cells shown in Figure 1K. Scale bar, 5 µm. (D) Cell proliferation rates of LL2 or LLC P cells treated with Veh or PTX (5 or 10 ng/mL) for 48 h, calculated using the formula: Hoechst^{+} cell number $^{\text{day 2}}$ / Hoechst^{+} cell number $^{\text{day 0}}$. (E-G) Representative fluorescence images of adherent LL2 or LLC cells stained with PI and Hoechst (E) and quantification of cell death (F, G) after a 48-h PTX (10 ng/mL) treatment. Cell death was calculated using the same formula as in Figure S1E for (F) and Figure S1G for (G). Scale bar, 20 µm. Note: all experiments were repeated at least three times. Error bars show the mean ± SD.

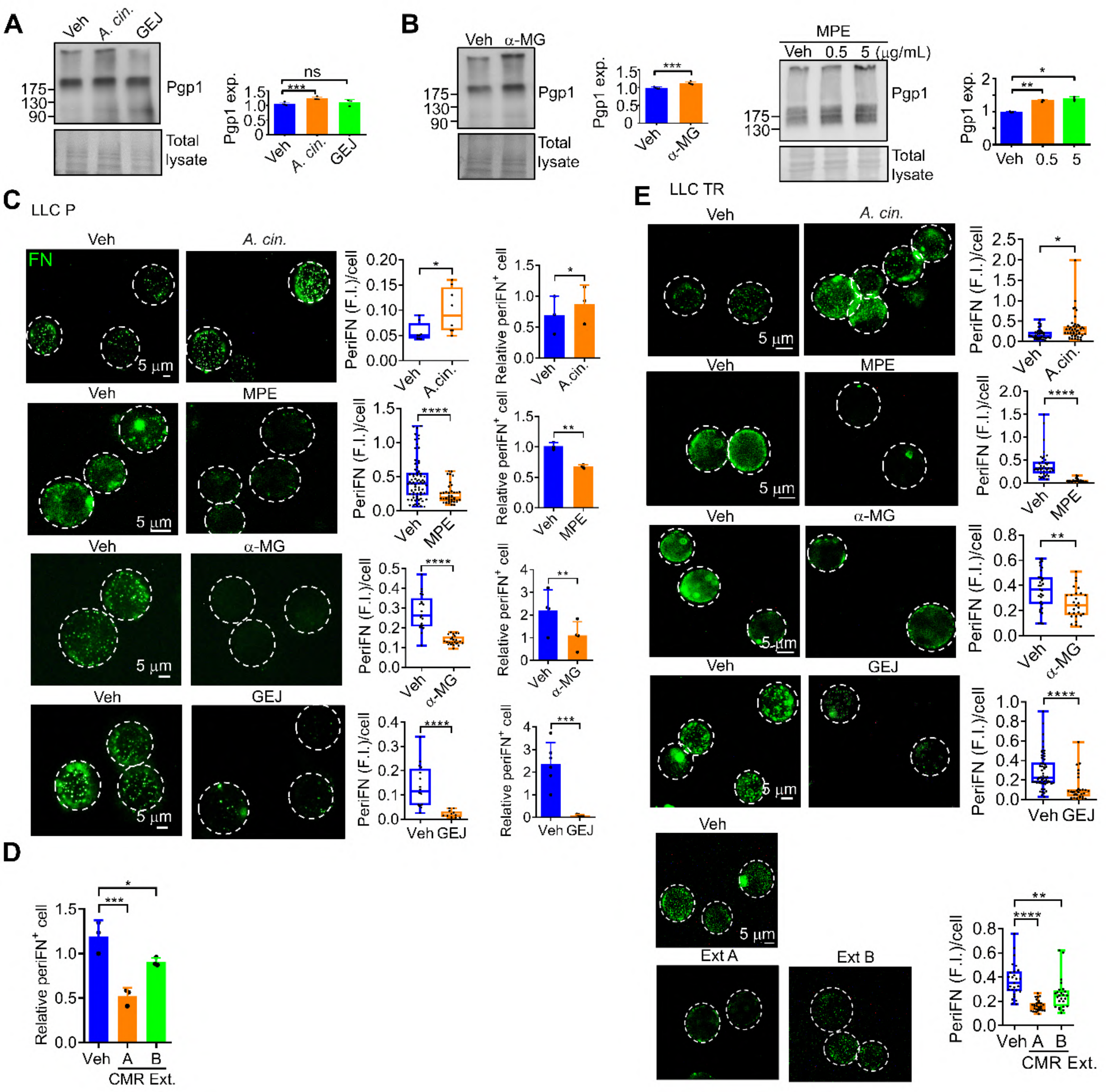

A
Veh
A. cin.
GEJ
175
130
90
Pgp1
Total lysate
Pgp1 exp.
ns
***
B
α-MG
MPE
Veh 0.5 5 (μg/mL)
C
LLC P
FN
5 μm
PeriFN (F.I.)/cell
Relative periFN+ cell
****
**
D
CMR Ext.
E
LLC TR
Ext A
Ext B

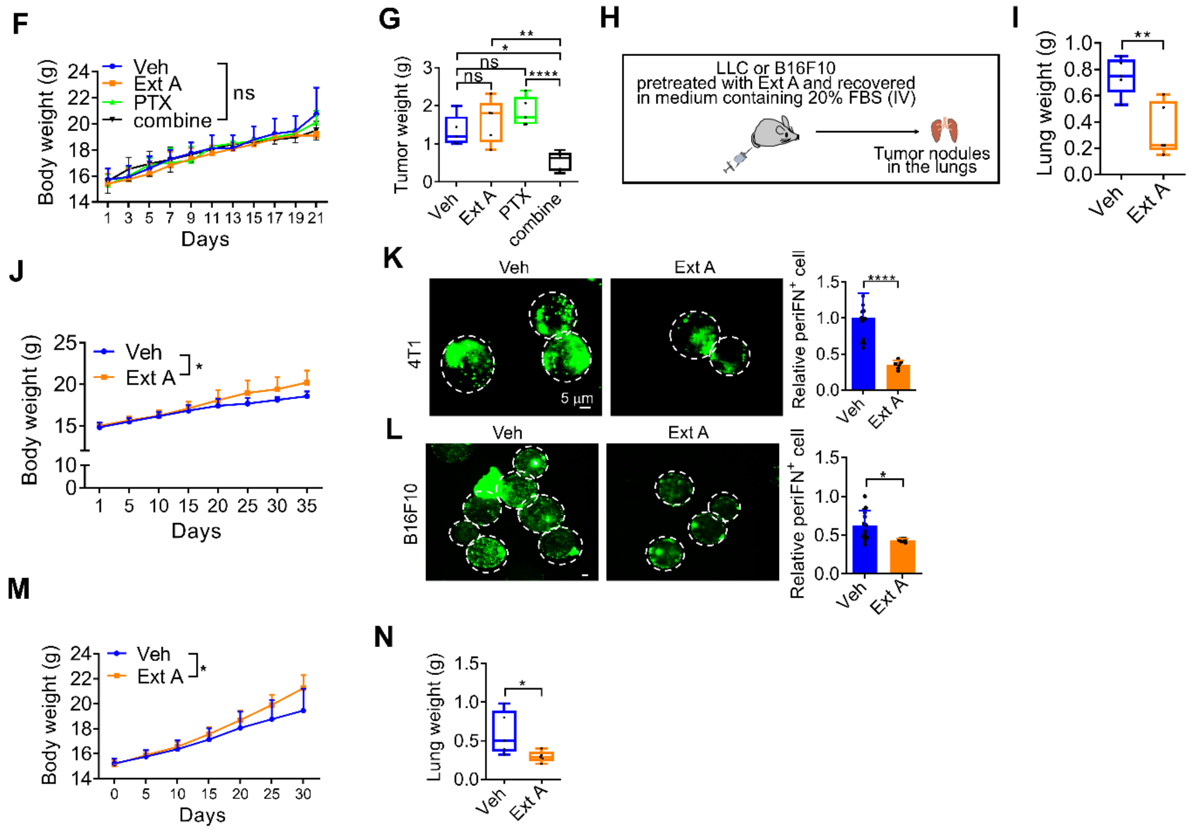

Figure. S3.

CMR uniquely inhibits both Pgp1 expression in ATCs and periFN assembly on STCs among all tested TCMs. (A, B) Representative IB images (left panels) and corresponding quantification (right panels) of Pgp1 expression in LLC TR ATCs treated with different TCMs. (C-E) Representative IF images (left panels), image quantification (middle panels) and FACS analysis-based quantification (right panels) of periFN assembly on LLC P STCs (C, D) and LLC TR STCs (E) treated with different TCMs. Scale bar, 5 μm. (F, G) Body weights (F) and tumor weights (G) of excised subcutaneous xenografts at the end of the study corresponding to Figure 2D. (H) Experimental schematic showing the evaluation of anti-metastatic effect of Ext A in B6 mice (related to Figure 2F-H, K and L), lung weights (I) and body weights (J) of excised subcutaneous xenografts at the end of the study. (K, L) Representative IF images (left panels) and FACS analysis-based quantification (right panels) corresponding to Figure 2I and 2J, respectively. Scale bar, 5 μm. (M, N) Body weight (M) and lung weights (N) of mice in Figure 1K. Concentrations used in this study: For ATCs: *Antrodia cinnamomea* (*A. cin.*), 1 mg/mL; Guilu Erxian Jiao (GEJ), 0.5 mg/mL; α-mangostin (α -MG), 12 μM; mangosteen pericarp extracts (MPE), 0, 0.5, and 5 μg/mL. For STCs: *A. cin.* and GEJ, 5 mg/mL; α-MG, 60 μM; MPE, 0.3 mg/mL. All concentrations were selected at non-cytotoxic doses, ensuring that they did not induce significant cell death. Note: all experiments were repeated at least three times. Error bars show the mean ± SD.

A

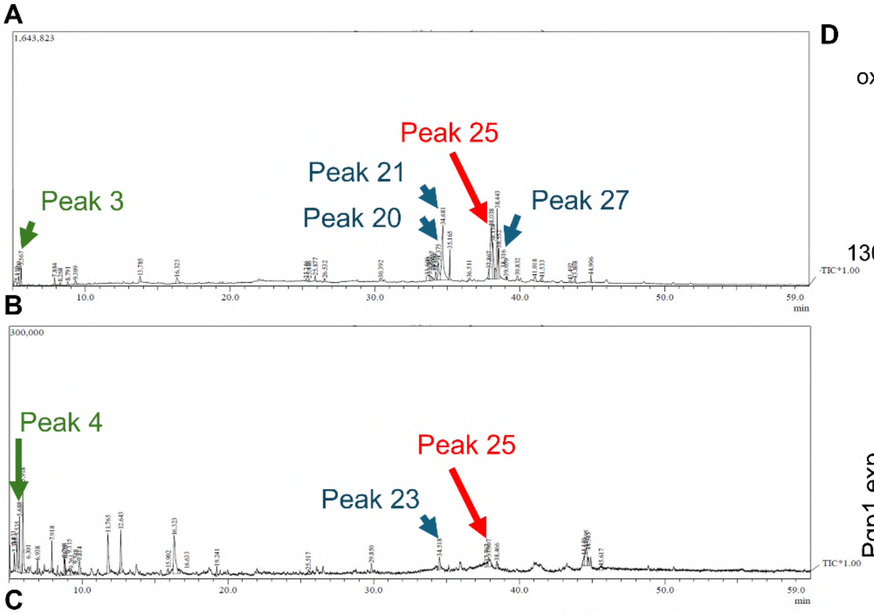

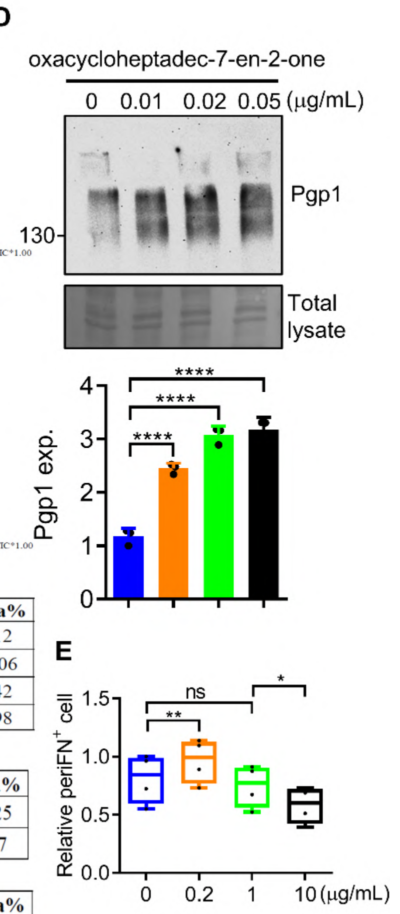

C

Formula	Identification
$C_{14}H_{28}O_2$	Tetradecanoic acid; Myristic acid
$C_{15}H_{30}O_2$	Pentadecanoic acid
$C_{16}H_{32}O_2$	Palmitic acid; Hexadecanoic acid

Ext	Peak	R.Time	Area%
A	20	34.375	5.12
A	21	34.681	18.06
A	27	38.316	2.42
B	23	34.518	2.98

Formula	Identification
$C_{16}H_{28}O_2$	Oxacycloheptadec-7-en-2-one
$C_{16}H_{28}O_2$	Oxacycloheptadec-8-en-2-one

Ext	Peak	R.Time	Area%
A	25	38.038	17.25
B	25	37.907	3.17

Formula	Identification
$C_7H_{16}O$	2-Hexanol, 2-methyl-

Ext	Peak	R.Time	Area%
A	3	5.567	1.78
B	4	5.688	5.68

Formula	Identification
$C_6H_6O_3$	Hydroxy methyl furfural
$C_7H_{10}S$	Thiophene, 2-propyl-

Ext	Peak	R.Time	Area%
A	9	16.323	1.73
B	18	16.323	12.71

Formula	Identification
$C_5H_4O_2$	Furfural; Furan-2-carboxaldehyde

Ext	Peak	R.Time	Area%
A	2	5.409	1.45
B	3	5.535	5.09

Formula	Identification
$C_8H_{22}O_3Si_2$	Disiloxane, 1,3-diethoxy-1,1,3,3-tetramethyl-

extract	Peak	R.Time	Area%
A	4	7.884	1.09
B	8	7.918	2.61

Figure. S4.

GC/MS-identified compounds enriched in CMR Ext A do not meet the inhibitory criteria. (A-C) GC/MS chromatograms of CMR Ext A (A) and Ext B (B). Arrows in (A, B) indicate peaks corresponding to the top three most abundant identified compounds (C), highlighting differences in their abundance between Ext A and B before comparing top fourth to sixth most abundant GC/MS-identified compounds. (D) Representative IB image (upper panels) and corresponding quantification (lower panel) of Pgp1 expression in LLC TR ATCs treated with oxacycloheptadec-7-en-2-one. (E) Quantification of FACS analysis for periFN assembly on LLC P STCs treated with

Oxacycloheptadec-7-en-2-one. Note: all experiments were repeated at least three times. Error bars show the mean ± SD.

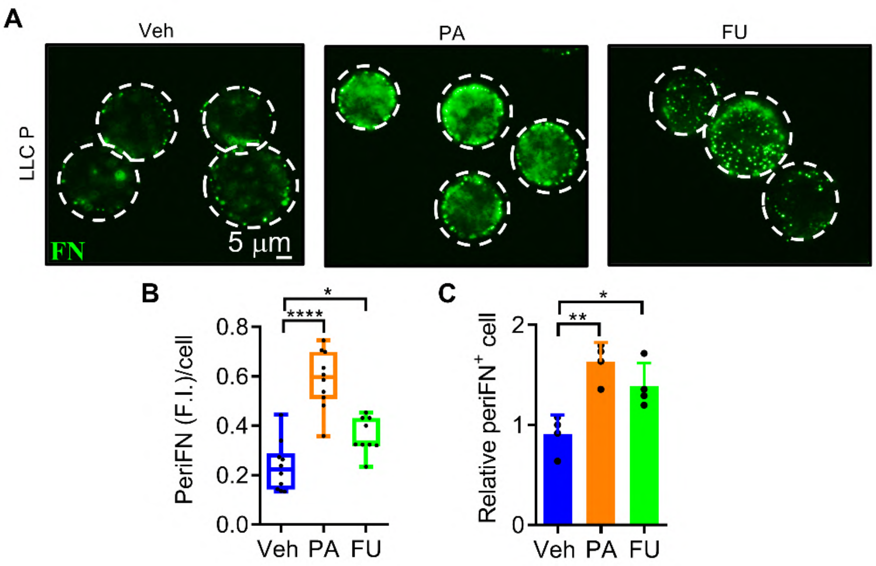

Figure. S5.

Palmitic acid and furfural, identified by GC/MS, are not responsible for the CMR Ext A-mediated inhibition of periFN assembly. (A-C) Representative IF images (upper panels) showing periFN assembly on LLC P STCs treated with 200 μM palmitic acid (PA) or furfural (FU). Scale bar, 5 μm. Quantifications of IF images (B) and FACS analysis (C) are shown in the lower left and lower right panels, respectively. Note: all experiments were repeated at least three times. Error bars show the mean ± SD.

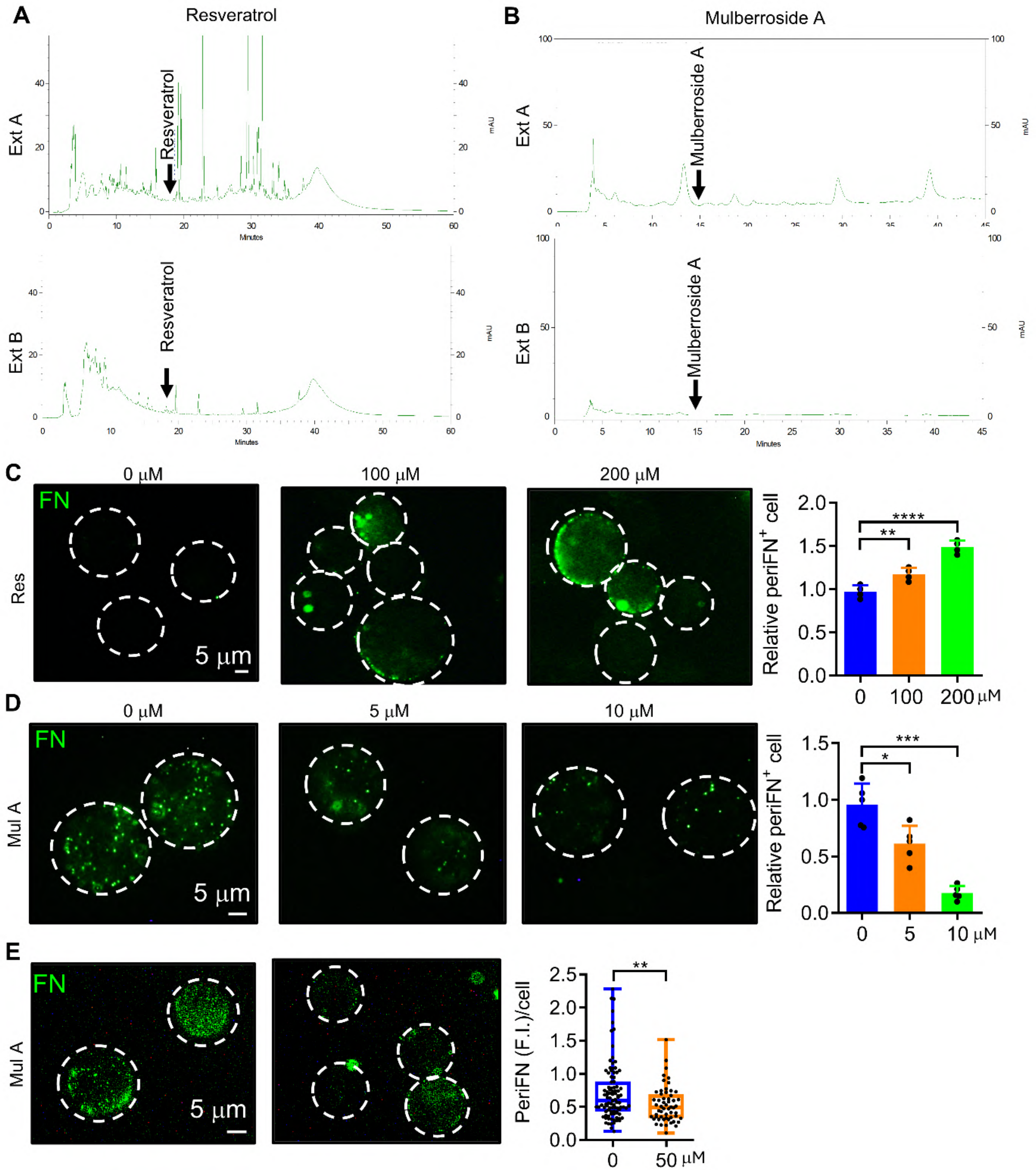

Figure. S6.

Mulberroside A (Mul A), but not Resveratrol (Res), is an active compound in CMR Ext A responsible for decreasing periFN assembly on LLC STCs cells. (A, B) HPLC chromatograms of Res (A) and Mul A (B) in CMR Ext A and Ext B. (C, D) Representative IF images (left panels) and corresponding FACS-based quantification (right panels) corresponding to Figure 3B, showing periFN assembly on LLC P STCs treated with Res (C) or Mul A(D), respectively. (E) Representative IF images (left panels) and corresponding quantification (right panel) of periFN assembly on LLC TR STCs treated with Mul A. Scale bar, 5 μm. Note: all experiments were repeated at least three times. Error bars show the mean ± SD.

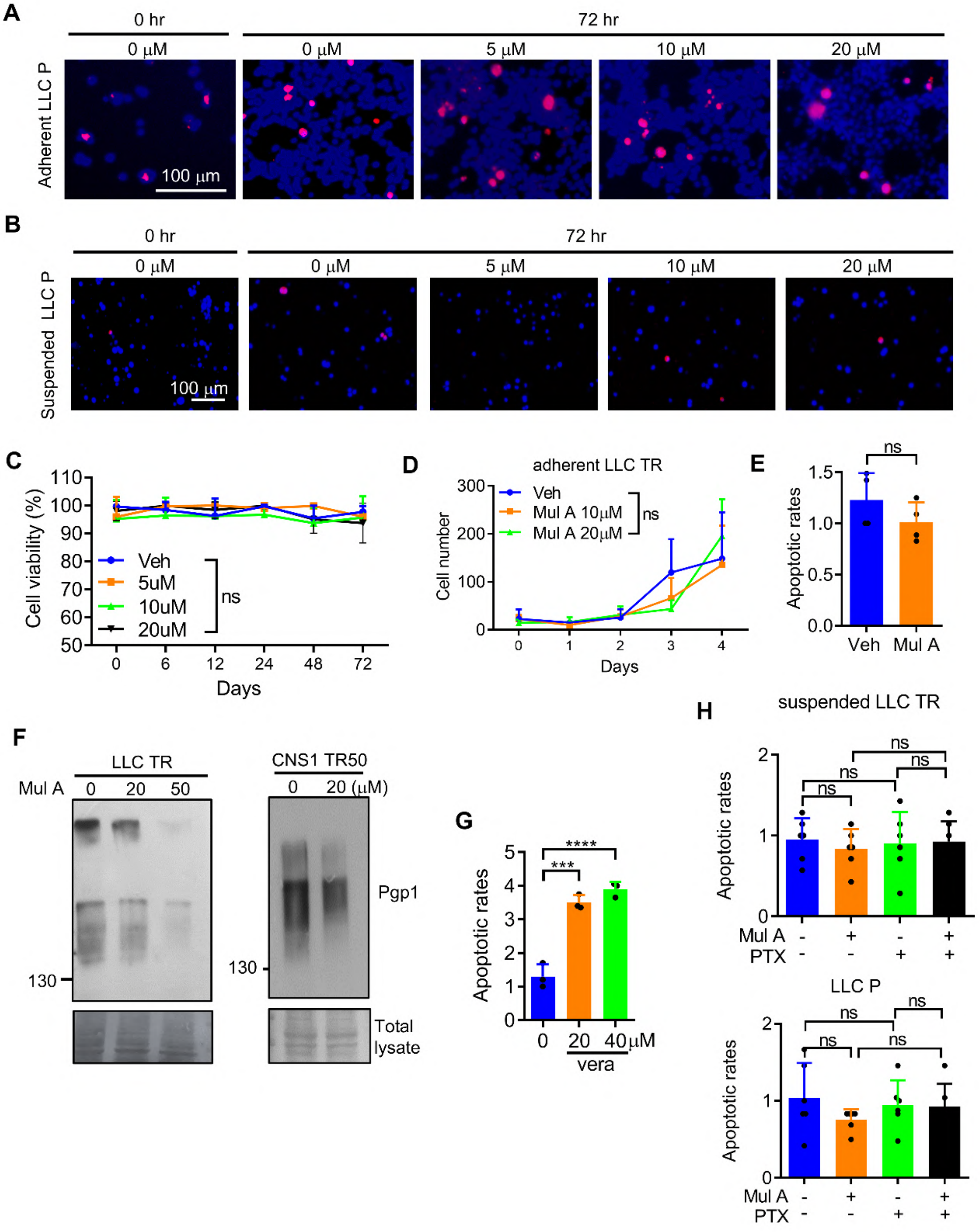

Figure. S7.

Mul A alone downregulates Pgp1 without inducing either apoptosis or restoring PTX sensitivity in LLC STCs. (A) Representative images corresponding to Figure 3C. Scale bar, 100 μm. (B) Representative images of LLC P STCs treated with Veh or increasing concentrations of Mul A for 24 h. Scale bar, 100 μm. (C) Quantification of cell viability in LLC P STCs after a 72-h treatment with Veh or various concentrations of Mul A. (D) Proliferation rates of LLC TR ATCs treated with Veh or varying concentrations of Mul A. (E) Apoptotic rates of LLC TR ATCs treated with Veh or Mul A (20 μM) for 48 h. (F) Representative IB images corresponding to Figure 3D.

(G) Apoptotic rates of LLC TR ATCs treated with various concentrations of vera. (H) Apoptotic rates of LLC TR (upper panel) and LLC P (lower panel) STCs treated with PTX (25 ng/mL) and/or Mul A (10 μM) for 48 h. Note: all experiments were repeated at least three times. Error bars show the mean ± SD.

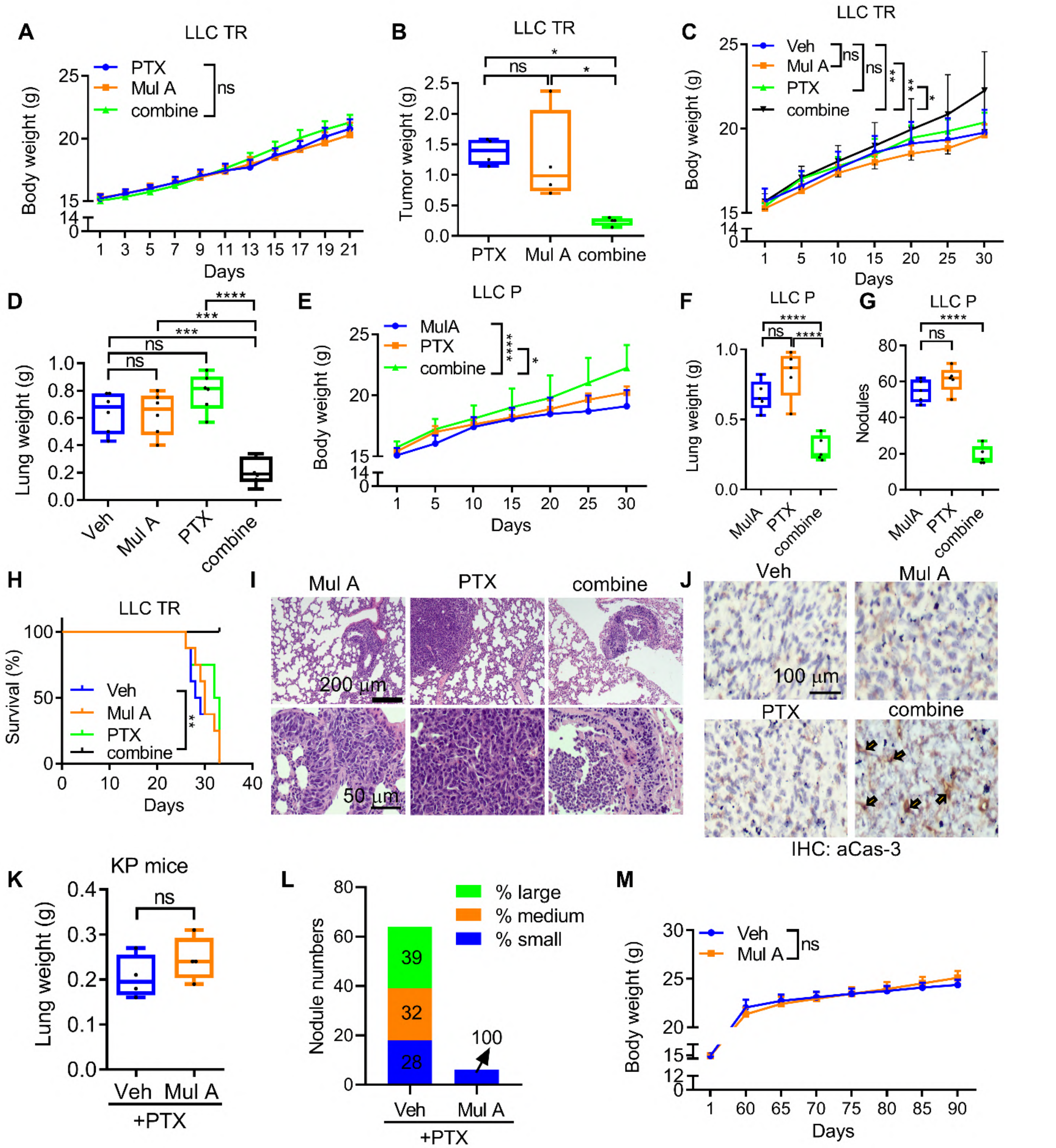

Figure. S8.

Mul A resensitizes pre-established lung tumor nodules derived from intravenously injected LLC P cells. (A, B) Body weights (A) and tumor weights (B) of B6 mice corresponding to Figure

4A. (C-G) Body weights (C for LLC TR cells; E for LLC P cells), lung weights (D for LLC TR cells; F for LLC P cells), and quantification of lung tumor nodules (G) related to Figure 4D. (H) Survival rates of B6 mice injected intravenously with LLC TR cells (related to Figure 4D). (I) H&E-stained histological images from B6 mice intravenously injected with LLC P cells (related to Figure 4D). Scale bar, upper panel, 200 μm; lower panel, 50 μm. (J) IHC staining of active caspase-3 (aCas-3) in tumor cells from the lungs shown in Figure 4H. Scale bar, 100 μm. (K-M) Lung weights (K), the sums and percentages of small (blue), medium (orange), and large (green) tumor nodules in each group (L), and body weights (M) of K-ras$^{LSL\text{-}G12D/+}$; p53$^{fl/fl}$ (KP) mice corresponding to Figure 4I. Note: all experiments were repeated at least three times. Error bars show the mean ± SD.

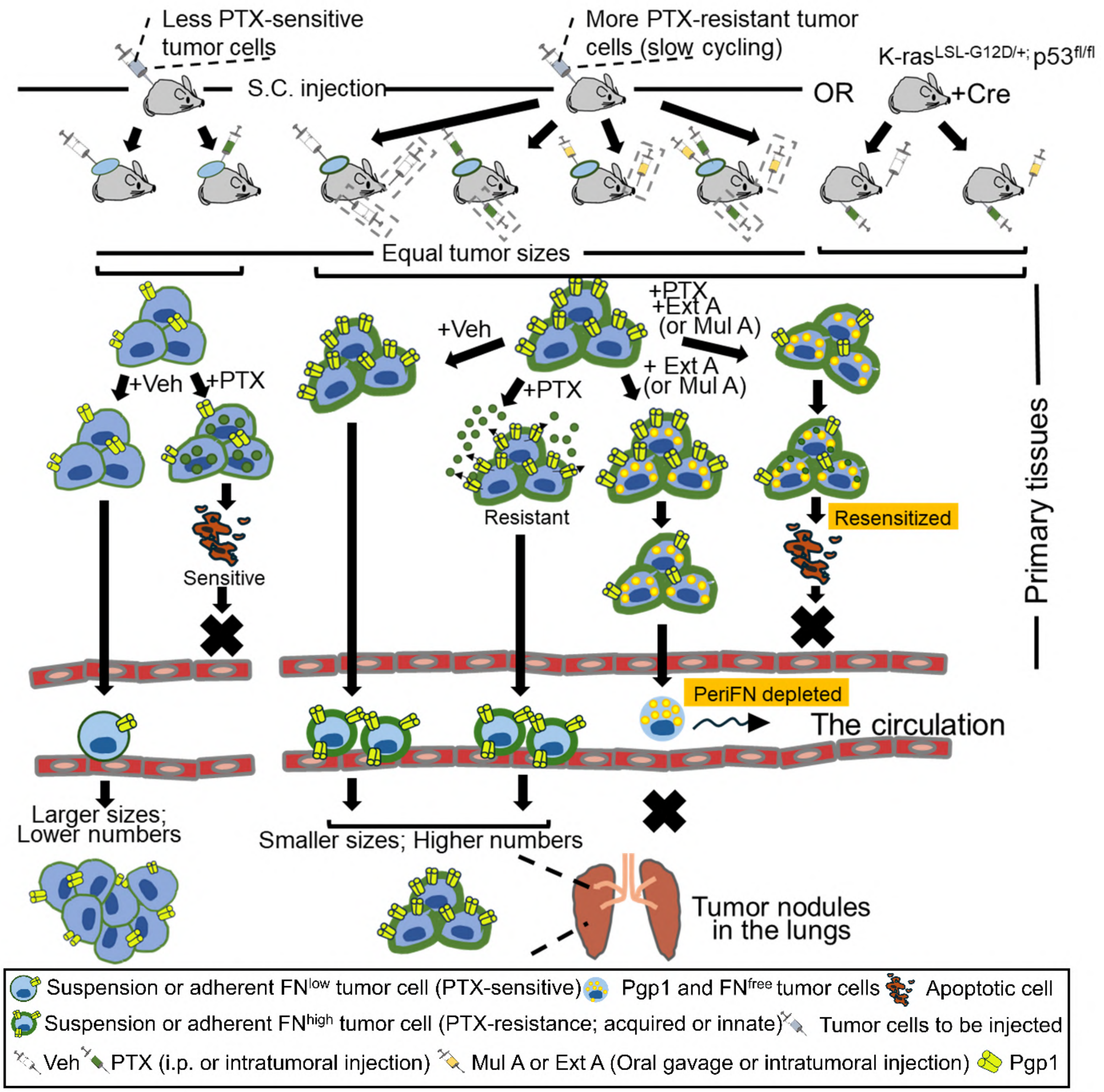

Figure. S9.

Schematic for the anti-metastatic effects of Mul A/Ext A in spontaneous tumor metastasis models. Less PTX-sensitive and more PTX-resistant tumor cells lead to *in vivo* equal subcutaneous tumor growths (Figure S1J), yet PTX treatment selectively reduced the volume of PTX-sensitive tumors (Figure 1F-I). CMR Ext A (oral gavage) and Mul A (IT injection or oral gavage) resensitized PTX-resistant subcutaneous tumors to PTX (administered either intratumorally or intraperitoneally), whereas Mul A alone sufficiently reduced lung-colonization by circulating PTX-sensitive or -resistant tumor cells (Figure 2D, E; Figure 4A-C; and Figure 4I-K).

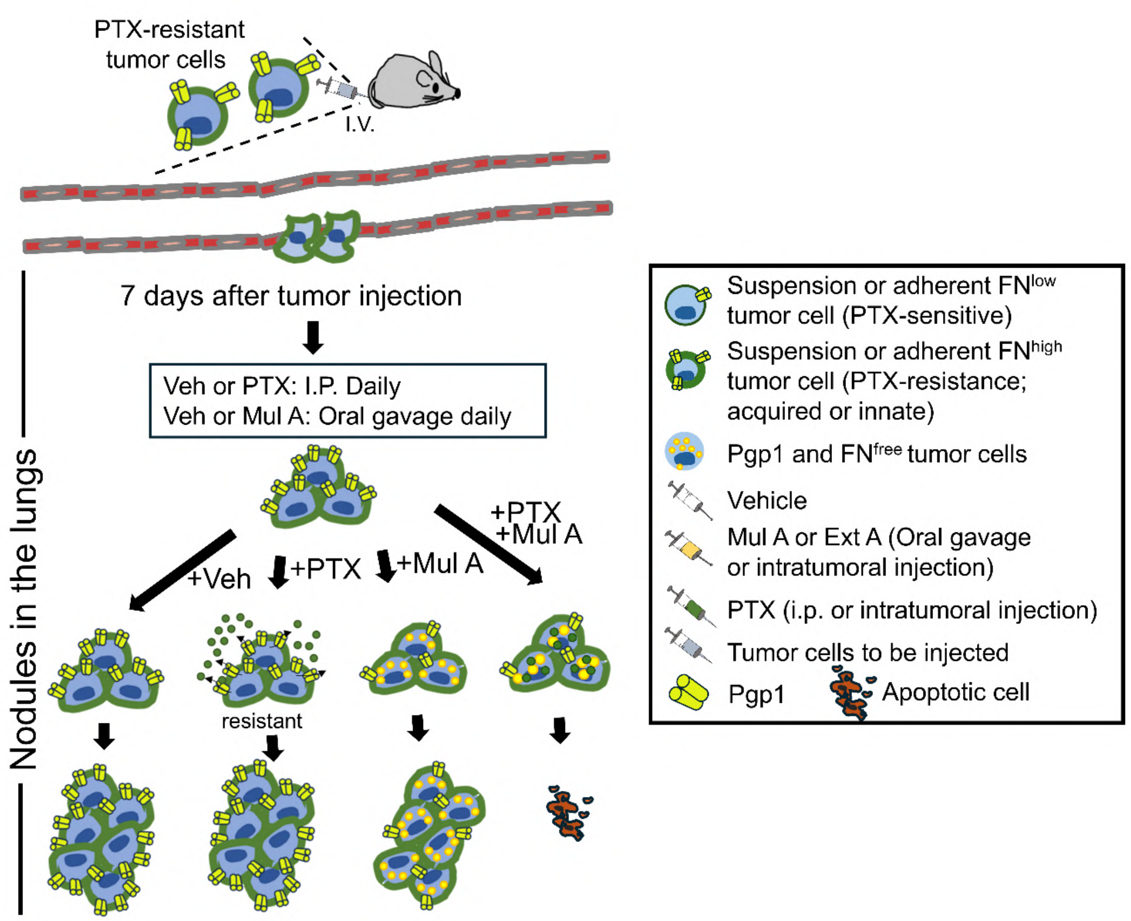

Figure. S10.

Schematic for the anti-metastatic effects of Mul A/Ext A on the pre-established tumors in the mouse lungs. Daily i.p. PTX combined with oral Mul A suppressed the outgrowth of pre-established nodules derived from i.v.-injected PTX-resistant tumor cells, whereas either agent alone had no effect (Figure 4D-G; Figure S8C-I).

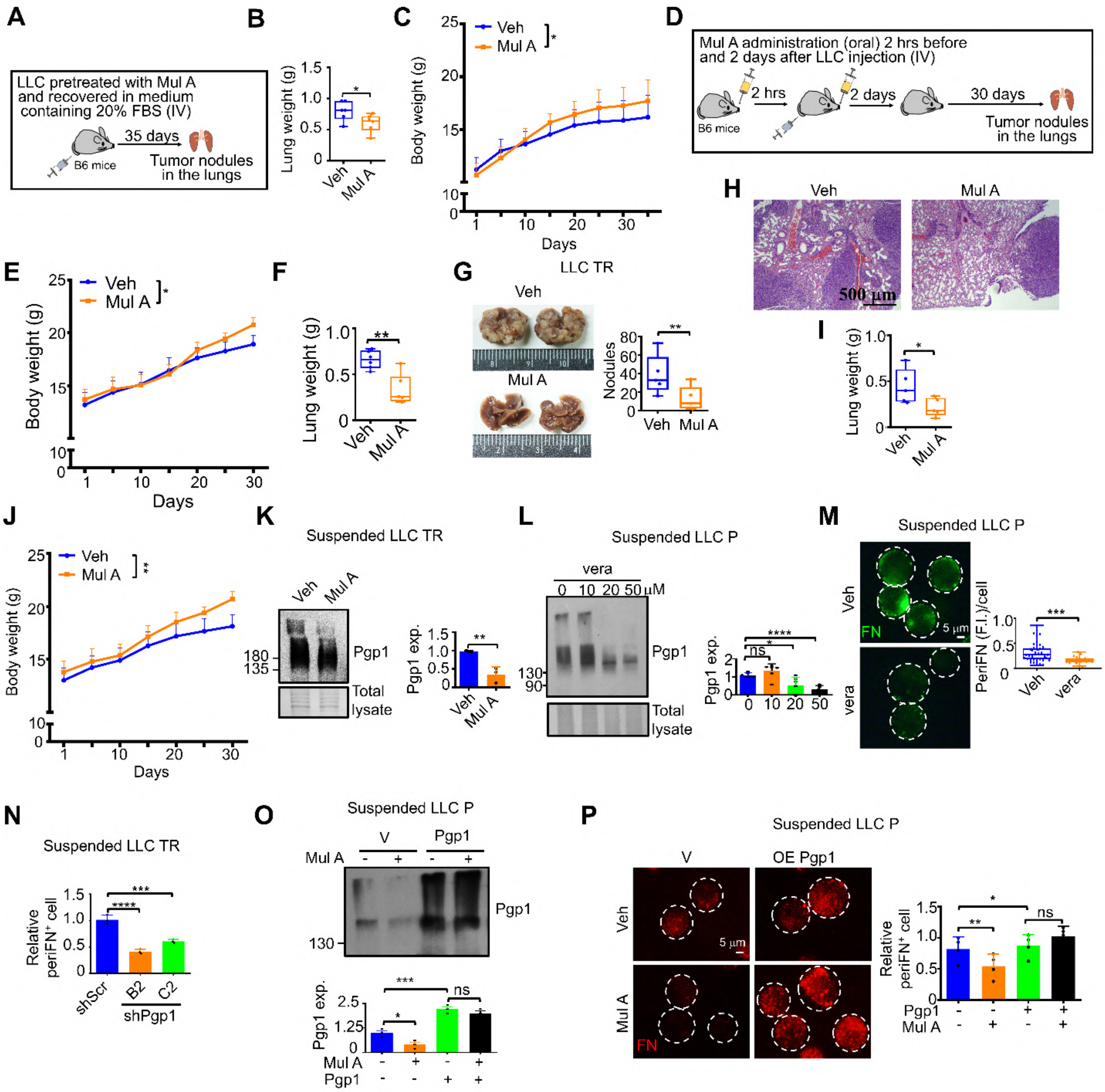

Figure. S11.

The anti-metastatic effect of Mul A is mediated through the inhibition of Pgp1-driven periFN assembly. (A-C) Experimental schematic for evaluation of the anti-metastatic effect of Mul A on LLC cells pretreated before injection into B6 mice (A), lung weights (B), and body weights (C) of B6 mice corresponding to (A) (related to Figure 5A-C). (D-F) Experimental schematic showing the anti-metastatic effect of Mul A in B6 mice (D), body weights (E) and lung weights (F) corresponding to (D; related to Figure 5D, E). (G) Representative images (left panel) and quantification (right panel) of lung tumor nodules from B6 mice administered Mul A (1.36 mg/kg, oral gavage) daily for 3 days (2 h before and 2 days after intravenous inoculation of LLC TR cells). (H-J) H&E-stained histopathological analysis of lung tissues (H; scale bar, 500 μm), lung weights (I), and body weights (J), corresponding to (G). (K) Representative IB images (left panel) and corresponding quantification (right panel) of Pgp1 expression in LLC TR STCs treated with Mul

A (100 μM) for 2 h. (L, M) Representative IB images of Pgp1 expression (L; left panel), IF images of periFN assembly (M; left panel; scale bar, 5 μm), along with corresponding quantifications (L and M; right panels), in LLC P STCs treated with various concentrations of vera (L) or 50 μM vera (M) for 2 h. (N) Quantification of periFN assembly based on FACS analysis in LLC TR STCs expressing shScr or shPgp1 (B2 or C2). (O) Representative IB images (upper panel) and quantification (lower panel) of Pgp1 expression in LLC P STCs transfected with either vector (V) or Pgp1 and treated with Veh or Mul A (10 μM) for 2 h. The loading control refers to the Coomassie staining of the same membrane used for the samples run on the same gel as in Figure 5L, which was probed with different antibodies. (P) Representative IF images (left panel; scale bar, 5 μm) and corresponding FACS analysis-based quantification of periFN assembly (right panel) on cells as treated in (O). Note: all experiments were repeated at least three times. Error bars show the mean ± SD.

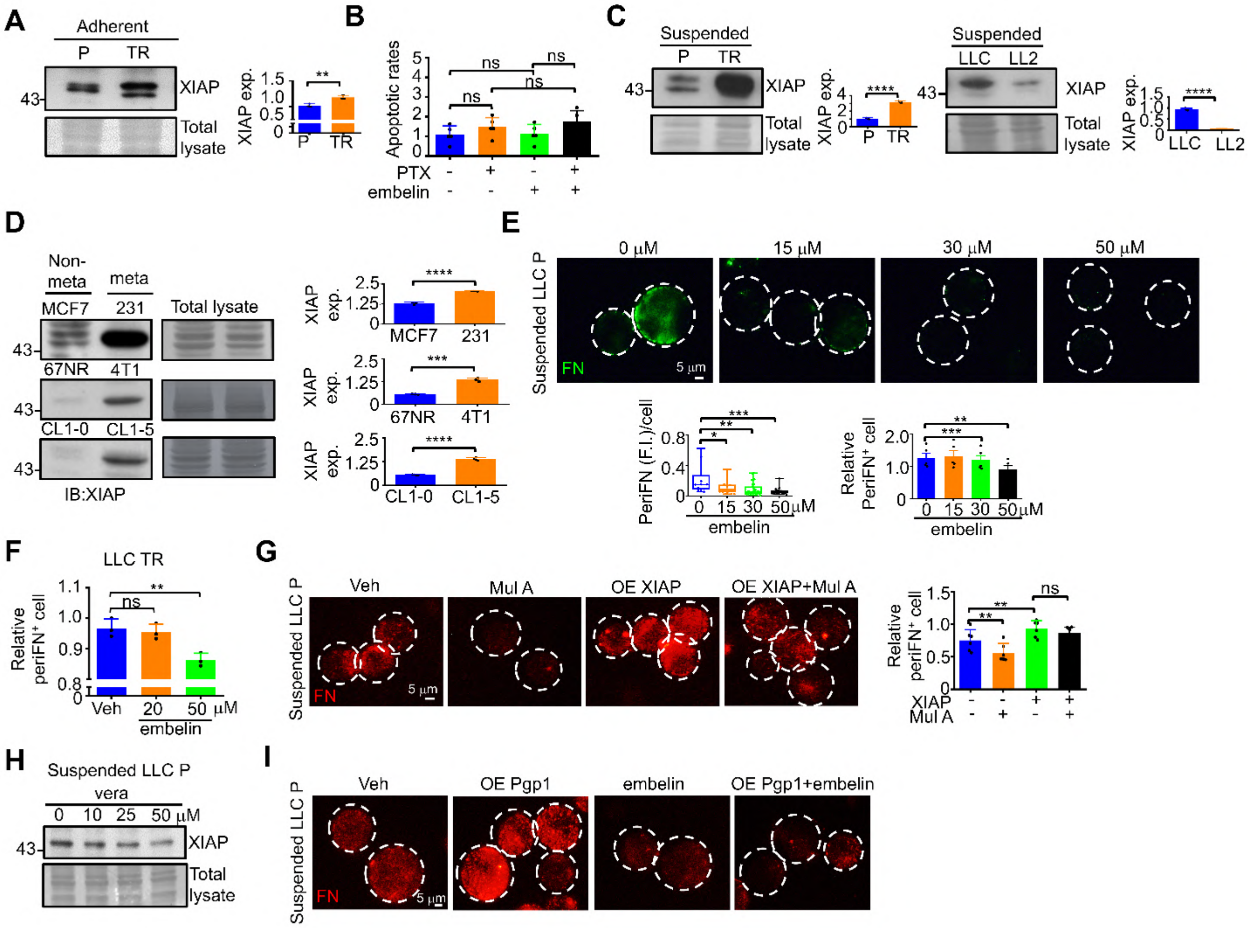

Figure. S12.

Mul A suppresses periFN assembly by inhibiting Pgp1-driven XIAP expression, which is upregulated in metastatic cancer cells. (A-C) Representative IB images (left panels) and corresponding quantification (right panels) of XIAP expression in LLC ATCs (A) and LLC STCs (C). Apoptotic rates of LLC TR ATCs treated with PTX (25 ng/mL) and/or embelin (15μM) for 48 h (B). (D) Representative IB images (left panels) and quantification (right panels) of XIAP expression in non-metastatic (non-meta, MCF7, 67NR, and CL1-0) and metastatic [meta, MDA-MB-231 (231), 4T1, and CL1-5] cell lines. (E) Representative IF images of LLC P STCs treated with various doses of embelin for 2 h (upper panels). Scale bar, 5 μm. Quantification was based on image analysis (lower left panel) and FACS analysis (lower right panel). (F) FACS-based quantification of periFN assembly on LLC TR STCs treated with embelin for 2 h. (G) Representative IF images (left panels; scale bar, 5 μm) and corresponding FACS-based quantification (right panel) of periFN assembly on LLC P STCs. (H) Representative IB images corresponding to Figure 5J. (I) Representative IF images corresponding to Figure 5K. Scale bar, 5 μm. Note: all experiments were repeated at least three times. Error bars show the mean ± SD.

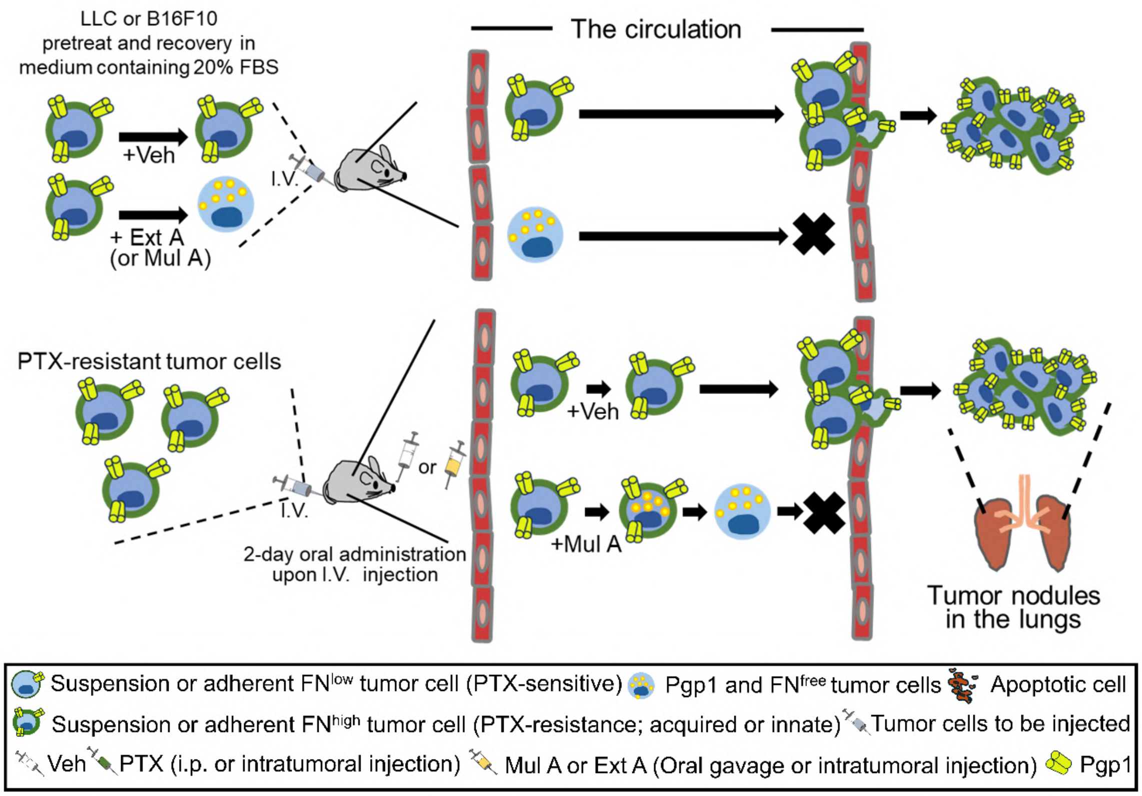

Figure. S13.

Schematic for the anti-metastatic effects of Mul A/Ext A in the circulation. Mul A pretreatment of metastatic tumor cells (Figure 2F-H, 2K, 2L and 5A-C) or two daily oral doses following intravenous tumor cell inoculation (Figure 5D-E; S11D-J) effectively prevented colonization of the lungs by blood-borne tumor cells.

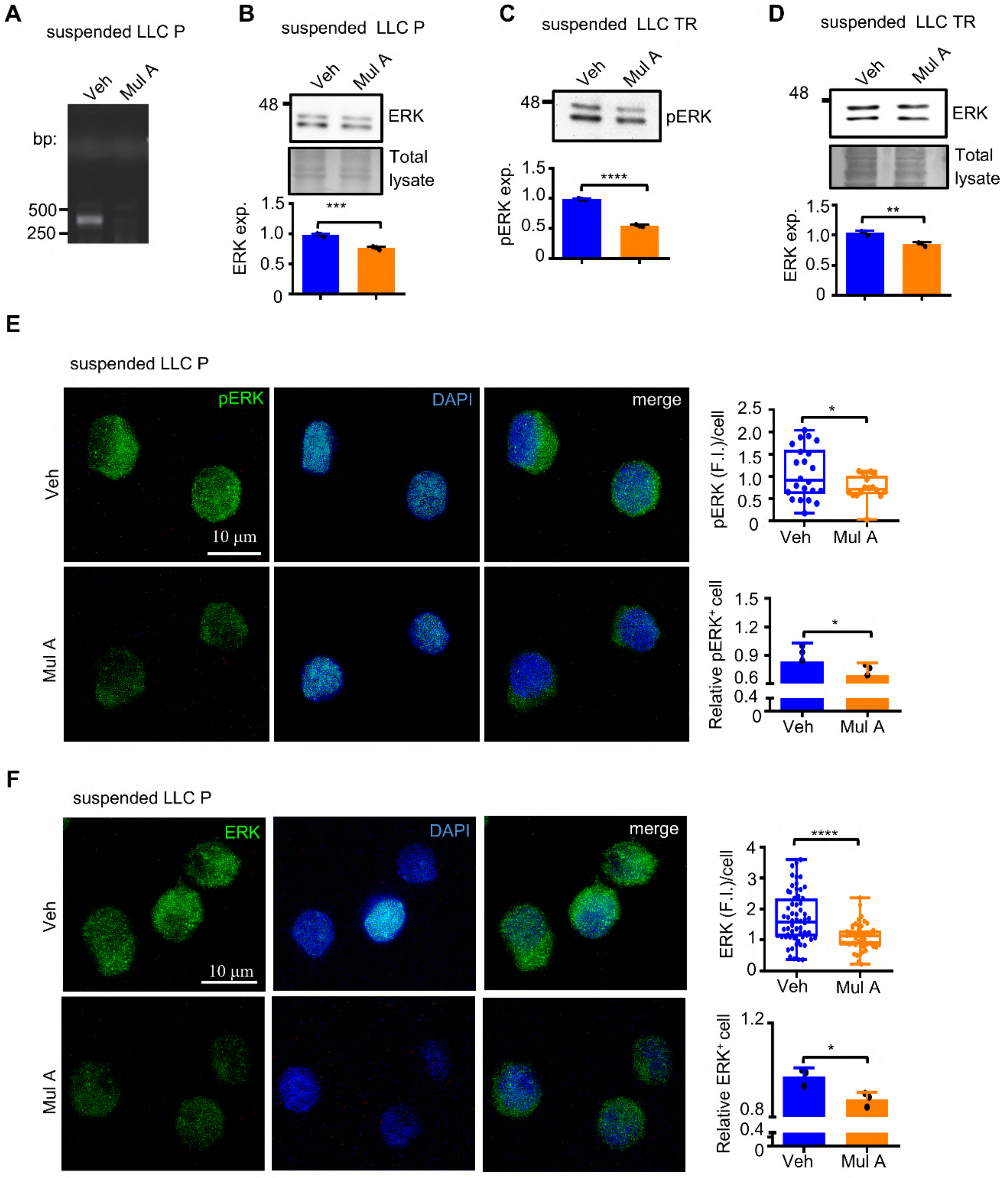
A
suspended LLC P
Veh
Mul A
bp:
500
250
B
suspended LLC P
Veh
Mul A
48
ERK
Total lysate
ERK exp.
1.5
1.0
0.5
0
***
C
suspended LLC TR
Veh
Mul A
48
pERK
pERK exp.
1.5
1.0
0.5
0
****
D
suspended LLC TR
Veh
Mul A
48
ERK
Total lysate
ERK exp.
1.5
1.0
0.5
0
**
E
suspended LLC P
pERK
DAPI
merge
Veh
Mul A
10 μm
pERK (F.I.)/cell
2.5
2.0
1.5
1.0
0.5
0
*
Veh
Mul A
Relative pERK⁺ cell
1.5
1.2
0.9
0.6
0.4
0
*
Veh
Mul A
F
suspended LLC P
ERK
DAPI
merge
Veh
Mul A
10 μm
ERK (F.I.)/cell
4
3
2
1
0
****
Veh
Mul A
Relative ERK⁺ cell
1.2
0.8
0.4
0
*
Veh
Mul A

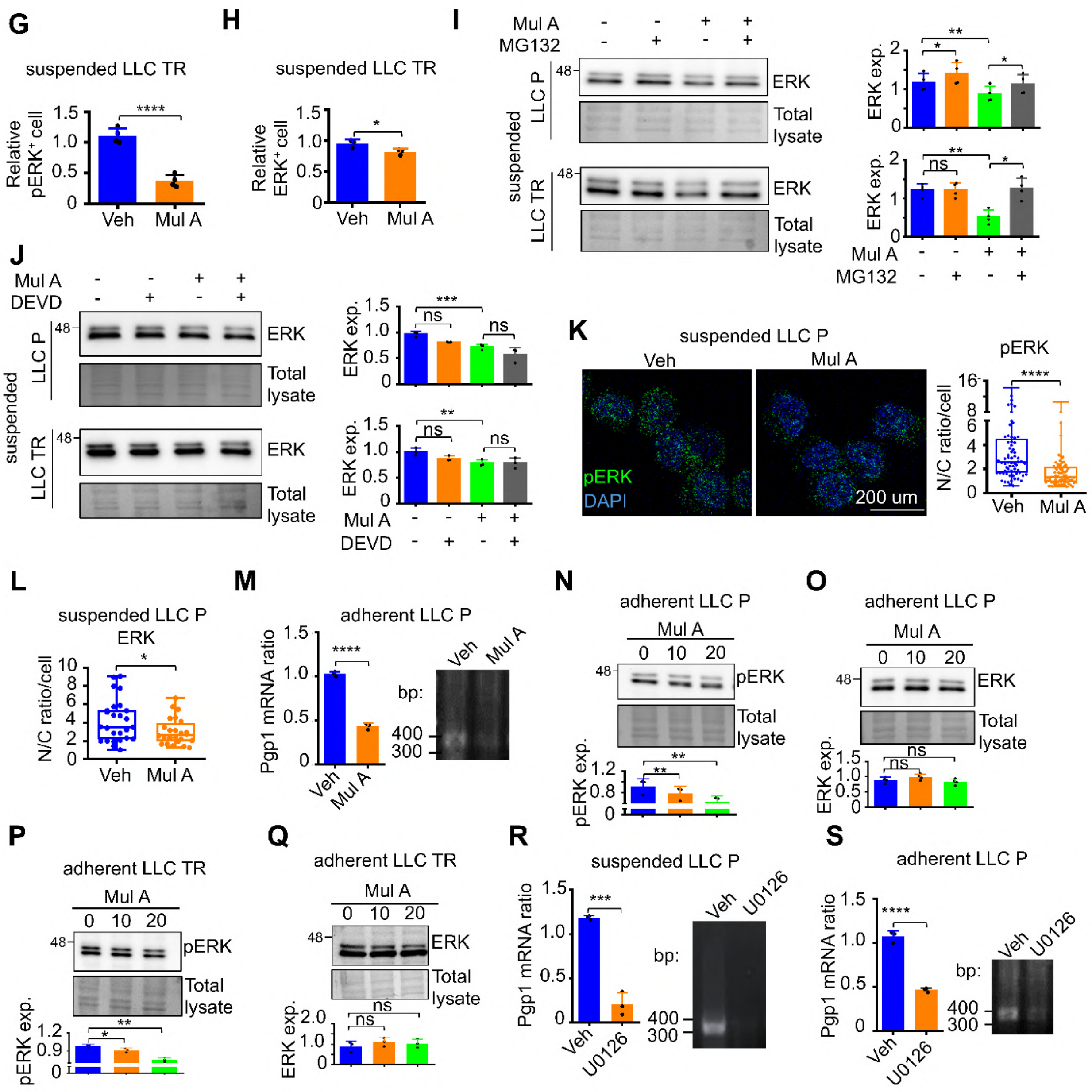
G
suspended LLC TR
Relative pERK+ cell
1.5
1.0
0.5
0
****
Veh
Mul A
H
suspended LLC TR
Relative ERK+ cell
*
Veh
Mul A
I
Mul A
MG132
suspended
LLC P
LLC TR
48
ERK
Total lysate
ERK exp.
**
*
ns
J
Mul A
DEVD
ERK
Total lysate
ERK exp.
***
**
ns
K
suspended LLC P
Veh
Mul A
pERK
DAPI
200 um
N/C ratio/cell
****
L
suspended LLC P
ERK
N/C ratio/cell
*
M
adherent LLC P
Pgp1 mRNA ratio
****
bp:
400
300
N
adherent LLC P
Mul A
0 10 20
pERK
Total lysate
pERK exp.
**
O
adherent LLC P
ERK
Total lysate
ERK exp.
ns
P
adherent LLC TR
pERK
Total lysate
pERK exp.
*
**
Q
adherent LLC TR
ERK
Total lysate
ERK exp.
ns
R
suspended LLC P
Pgp1 mRNA ratio
***
Veh
U0126
S
adherent LLC P
Pgp1 mRNA ratio
****
Veh
U0126

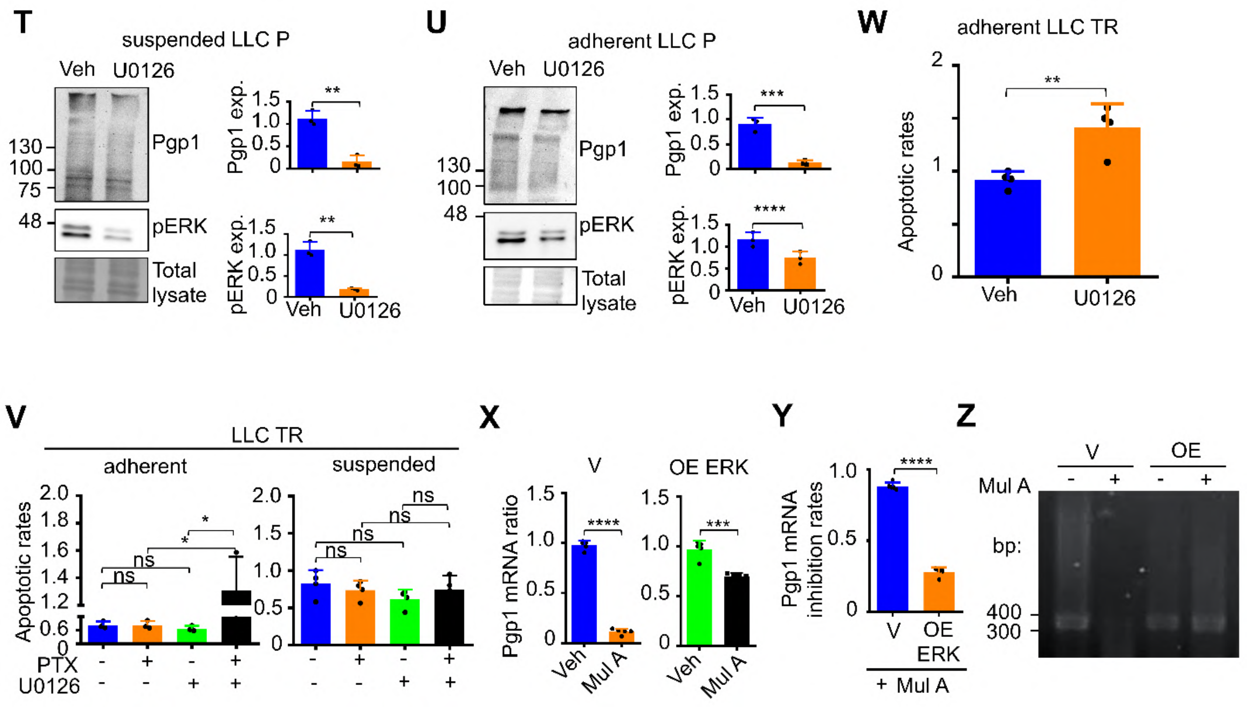

Figure. S14.

ERK-upregulated Pgp1 expression mediates the Mul A-reversible LLC PTX resistance. (A-B) Representative agarose gel image of RT-qPCR products for *Pgp1* mRNA (A; corresponding to Figure 5M) and IB image of ERK (B) in LLC P STCs treated with Mul A (20 μM) for 2 h. (C, D) Representative IB images of pERK (C) and ERK (D) in LLC TR STCs treated with Mul A (100 μM) for 2 h. The loading control in (C) refers to the Coomassie staining of the same membrane used for the samples run on the same gel as in Figure. S11K, which was probed with different antibodies. (E, F) Representative IF images (left panels; scale bar, 10 μm) and image-based quantifications (upper right panels) of pERK (E) and ERK (F) in LLC P STCs treated with Mul A (100 μM) for 2 h. The lower right panels show the corresponding FACS-based quantification of pERK and ERK, respectively. (G, H) FACS-based quantifications of pERK (G) and ERK (H) in LLC TR STCs treated with Mul A (100 μM) for 2 h. (I, J) Representative IB images (left panels) and corresponding quantification (right panels) of ERK expression in LLC P (upper panels) and TR (lower panels) STCs treated with Mul A (P: 20 μM; TR: 100 μM) alone or in combination with MG132 (I; 20 μM) or Z-DEVD-FMK (J; 20 μM) for 2 h. (K) Representative IF images (left panels; scale bar, 200 μm) and corresponding quantification (right panel) of the nuclear-to-cytoplasmic pERK intensity ratios from confocal microscopy images of LLC P STCs treated with Veh or MulA (100 μM) for 2 h. (L) Image-based quantification of the nuclear-to-cytoplasmic ERK intensity ratios in LLC P STCs treated with Veh or Mul A (100 μM) for 2 h. (M) Quantification (left panel) and representative agarose gel image (right panel) of RT-qPCR products for *Pgp1* mRNA in LLC ATCs treated with Mul A (20 μM) for 48 h. (N-Q) Representative IB images (upper panels) and quantification (lower panels) of pERK (N; LLC P, P; LLC TR) and ERK (O; LLC P, Q; LLC TR) expression in LLC ATCs treated with various Mul A for 48 h. (R, S) Quantification (left panel) and representative agarose gel image (right panel) of RT-qPCR products for *Pgp1* mRNA in LLC P STCs (R) or ATCs (S) treated with U0126 (2 μM) for 48 h. (T) Representative IB images (left panels) and corresponding quantification (right panels) of Pgp1 and pERK

expression in LLC P STCs (T) or ATCs (U) treated with U0126 (2 μM) for 48 hrs. (V) Apoptotic rates of LLC TR cells treated with PTX (25 ng/mL) and/or U0126 (2 μM) for 48 h (ATCs) or 24 h (STCs). (W) Apoptotic rates of LLC TR ATCs treated with Veh or U0126 (5 μM) for 48 h. (X) Quantification of RT-qPCR products for *Pgp1* mRNA in LLC STCs transfected with vector (V) or ERK (OE), and/or treated with Mul A (20μM) for 2h. (Y) Inhibition rates of *Pgp1* mRNA under the same conditions as in (X). (Z) Representative agarose gel image of RT-qPCR products for *Pgp1* mRNA corresponding to (X). Note: all experiments were repeated at least three times. Error bars show the mean ± SD.

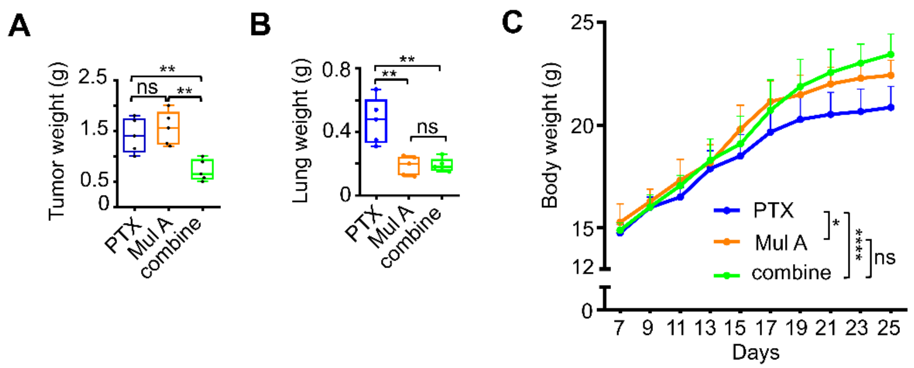

Figure. S15.
Oral Mul A, either alone or combined with i.p. PTX, prevents the body weight loss observed in PTX-treated tumor-bearing mice. (A-C) Tumor weights of excised subcutaneous xenografts (A), lung weights (B), and body weights (C) of B6 mice corresponding to Figure 7A. Note: all experiments were repeated at least three times. Error bars show the mean ± SD.

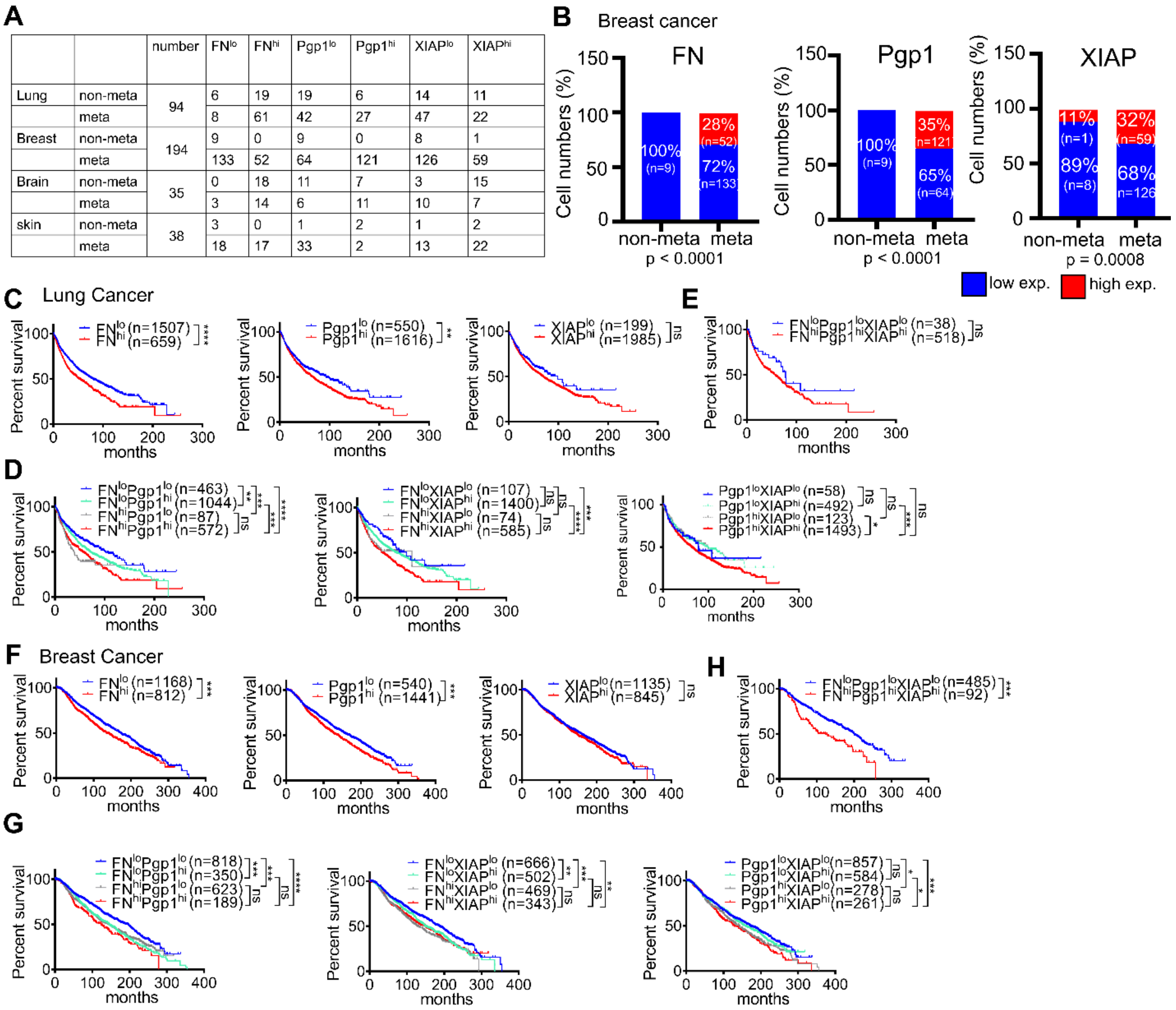

		number	FN^{lo}	FN^{hi}	$Pgp1^{lo}$	$Pgp1^{hi}$	$XIAP^{lo}$	$XIAP^{hi}$
Lung	non-meta	94	6	19	19	6	14	11
	meta		8	61	42	27	47	22
Breast	non-meta	194	9	0	9	0	8	1
	meta		133	52	64	121	126	59
Brain	non-meta	35	0	18	11	7	3	15
	meta		3	14	6	11	10	7
skin	non-meta	38	3	0	1	2	1	2
	meta		18	17	33	2	13	22

Figure. S16.

Combining expressions of *FN* and *Pgp1* enhances their prognostic value. (A) A table summarizing the number of cell lines across the four tested cancer types, included in both the Cancer Cell Line Encyclopedia (CCLE) and the Metastasis Map database, categorized by non-metastasis (non-meta) or metastasis (meta) status. The mRNA expression levels of *FN*, *Pgp1*, or *XIAP* were classified as high (hi) and low (lo) based on ROC analysis as described in Figure 8B. (B) Bar charts illustrate the percentages of breast cancer cell lines from (A) exhibiting high or low expression of *FN*, *Pgp1*, and *XIAP* in non-meta and meta cells. (C-H) Kaplan–Meier curves showing overall survival (OS) of lung cancer patients (C-E) and breast cancer patients (F-H) stratified by *FN*, *Pgp1*, and *XIAP* gene expression levels.

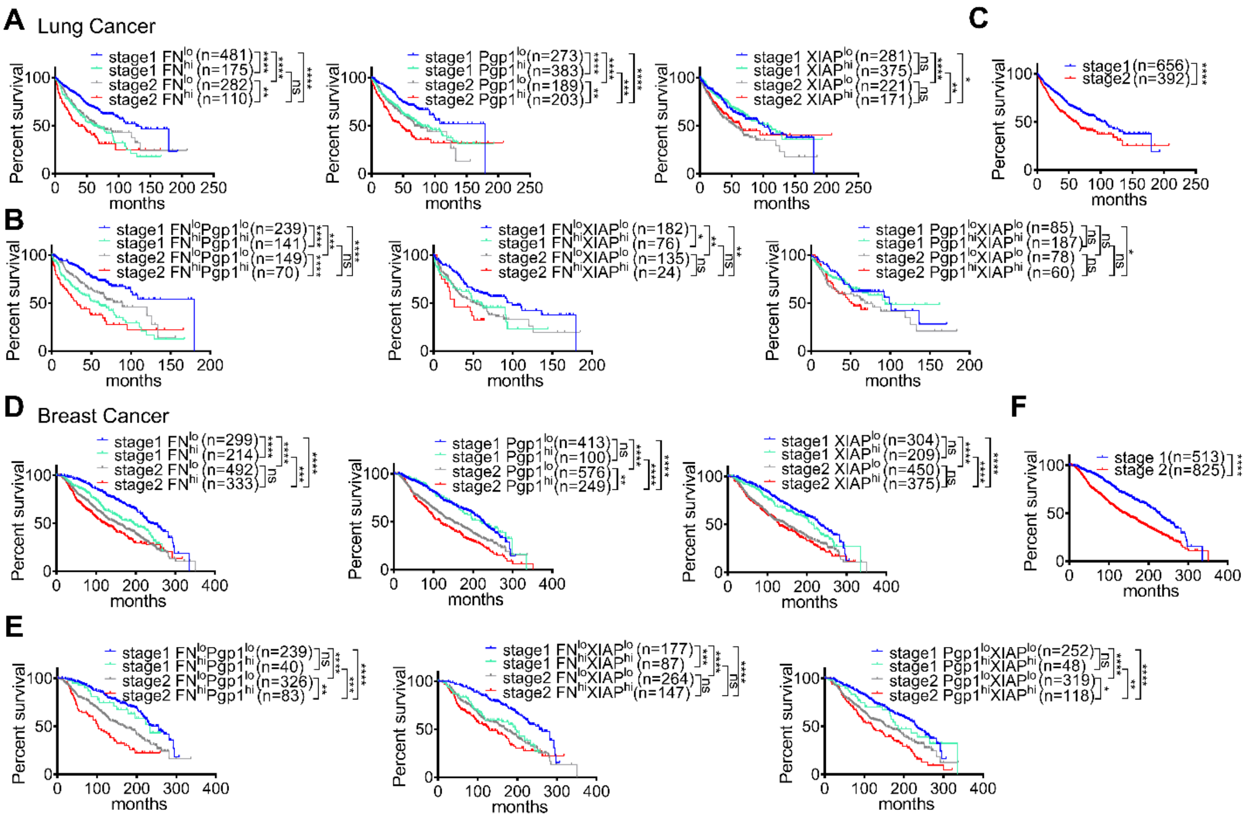

Figure. S17.

Combining expressions of *FN* and *Pgp1* enhances prognostic value in early-stage cancer patients. (A-F) Kaplan–Meier curves showing OS of lung (A-C) and breast (D-F) cancer patients with clinical stage 1 or 2.

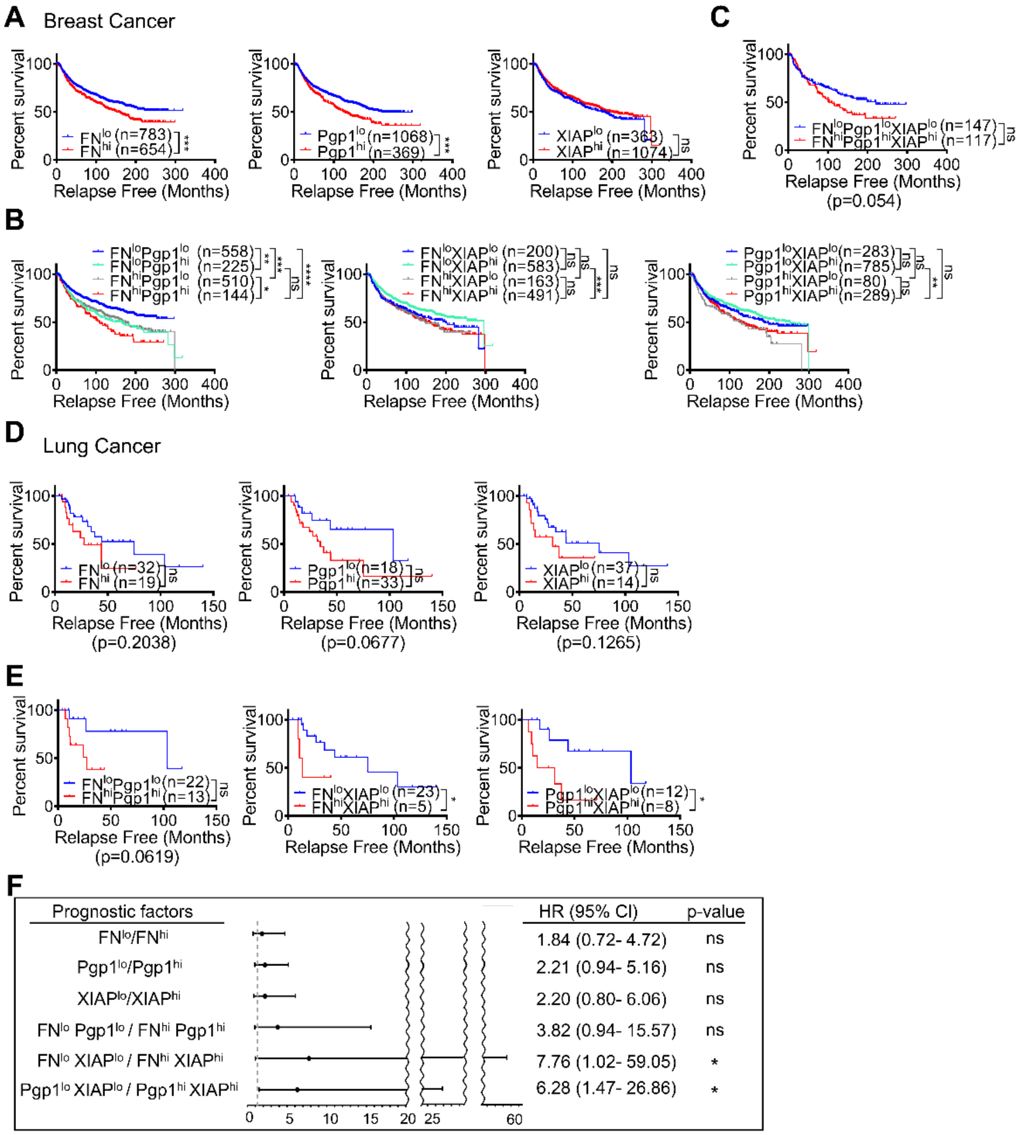

Figure. S18.

Combining *FN* and *Pgp1* expression levels predicts cancer relapse following anti-cancer therapies. (A-E) Kaplan–Meier curves depicting 20-year relapse-free survival (RFS) in breast (A-C) and lung (D-E) cancer patients treated with various therapies, stratified by expression levels of *FN*, *Pgp1*, and *XIAP*. (F) Forest plot displaying hazard ratios (HR) for RFS in lung cancer patients corresponding to (D-E).

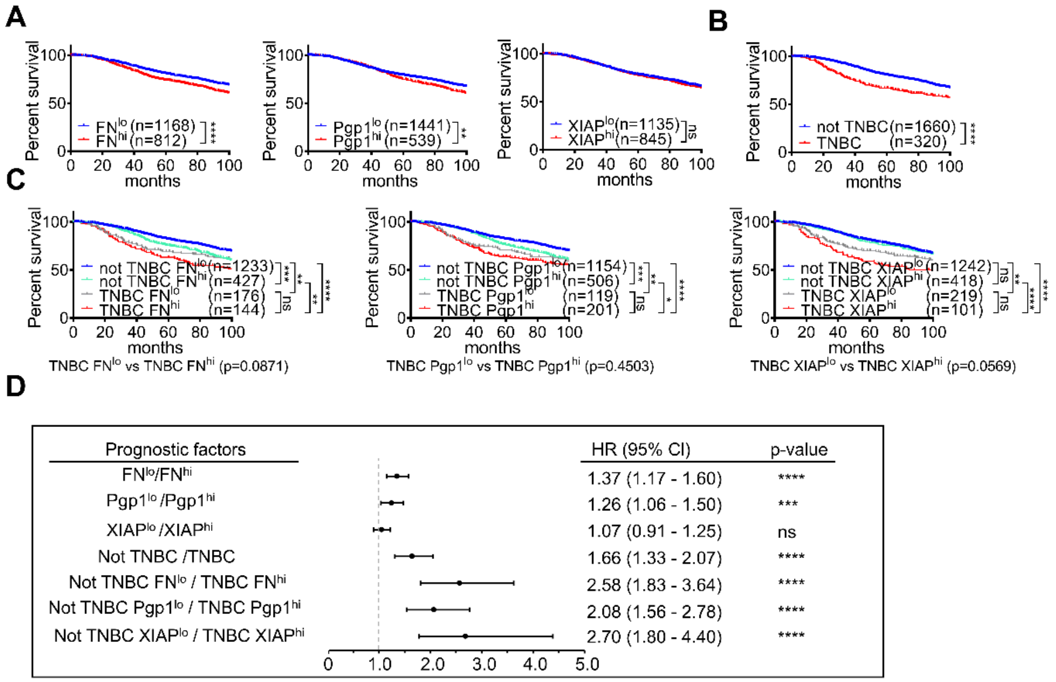

Figure. S19.

Combining expressions of *FN* and *Pgp1* enhances prognostic accuracy in patients with triple-negative breast cancer (TNBC). (A-B) Kaplan–Meier curves showing OS within 100 months for all breast cancer patients stratified by the expression levels of *FN*, *Pgp1*, or *XIAP* (A) and for patients with or without TNBC (B). (C) Kaplan–Meier curves stratified by *FN*, *Pgp1*, *XIAP* expression levels, and TNBC status, considering all four as prognostic indicators. (D) Forest plot corresponding to (A-C).

Table S1. The GC/MS analysis results of CMR extract A, including retention times and peak area percentages for the detected compounds.

**Table 1**	**extract A**			
**Peak**	**Retention Time**	**Area%**	**Formula**	**Identification**
1	5.11	2.1	unknown	
2	5.409	1.45	$C_5H_4O_2$	Furfural; Furan-2-carboxaldehyde
3	5.567	1.78	$C_7H_{16}O$	2-Hexanol, 2-methyl-; 2-Pentanol, 2,4-dimethyl-; 2-Pentanol, 2,3-dimethyl-
4	7.884	1.09	$C_8H_{22}O_3Si_2$	Disiloxane, 1,3-diethoxy-1,1,3,3-tetramethyl-
5	8.268	0.3	C_6H_6O2	2-Furancarboxaldehyde;Furfural <5-methyl->
6	8.791	0.75	unknown	
7	9.309	1.27	$C_6H_{12}O_2$	Hexanoic acid
			$C_5H_{10}O_2$	Valeric acid; Pentanoic acid
8	13.785	1.28	unknown	
9	16.323	1.73	$C_6H_6O_3$	Hydroxy methyl furfural; 2-Furancarboxaldehyde, 5-(hydroxymethyl)-
			$C_7H_{10}S$	Thiophene, 2-propyl-
10	25.246	0.34	$C_{15}H_{26}O$	1,6,10-Dodecatrien-3-ol, 3,7,11-trimethyl-; Nerolidol
11	25.446	0.44	$C_{15}H_{26}O$	1,6,10-Dodecatrien-3-ol, 3,7,11-trimethyl-; Nerolidol
12	25.877	0.72	$C_{12}H_{14}O_4$	Diethyl Phthalate
13	26.532	0.66	$C_9H_{12}O_4$	Phenol, 3,4,5-trimethoxy-
14	30.392	1.04	$C_{12}H_{36}O_6Si_6$	Cyclohexasiloxane, dodecamethyl-
15	33.6	2.42	unknown	
16	33.767	0.97	unknown	
17	33.917	1.06	$C_{14}H_{22}N_2O_2$	5,10-Diethoxy-2,3,7,8-tetrahydro-1H,6H-dipyrrolo[1,2-a;1',2'-d]pyrazine
18	34.033	0.56	unknown	
19	34.179	1.83	$C_{20}H_{30}O_4$	1,2-Benzenedicarboxylic acid, butyl octyl ester
			$C_{10}H_{10}O_3$	Benzoic acid, 2-(1-oxopropyl)-
			$C_{21}H_{28}O_4$	Phthalic acid, isobutyl non-5-yn-3-yl ester
20	34.375	5.12	$C_{16}H_{32}O_2$	Palmitic acid; Hexadecanoic acid
			$C_{15}H_{30}O_2$	Pentadecanoic acid
			$C_{14}H_{28}O_2$	Tetradecanoic acid; Myristic acid
			$C_{13}H_{26}O_2$	Tridecanoic acid

21	34.681	18.06	$C_{16} H_{32} O_2$	Palmitic acid; Hexadecanoic acid
			$C_{15}H_{30}O_2$	Pentadecanoic acid
			$C_{14}H_{28}O_2$	Tetradecanoic acid; Myristic acid
			$C_{13}H_{26}O_2$	Tridecanoic acid
22	35.165	3.66	$C_{18}H_{36}O_2$	Palmitic acid, ethyl ester; Hexadecanoic acid, ethyl ester
			$C_{16}H_{32}O_2$	Myristate <ethyl->;
			$C_{17}H_{34}O_2$	Pentadecanoic acid, ethyl ester
			$C_{20}H_{40}O_2$	Stearate <ethyl->;
23	36.511	0.59	$C_{16}H_{48}O_6Si_7$	Heptasiloxane, hexadecamethyl-
			$C_{16}H_{50}O_7Si_8$	Octasiloxane, 1,1,3,3,5,5,7,7,9,9,11,11,13,13,15,15-hexadecamethyl-
			$C_{18}H_{54}O_9Si_9$	Cyclononasiloxane, octadecamethyl-
24	37.867	1.65	$C_{12}H_{22}O$	Dodec-2(E)-enal
			$C_{18}H_{34}O$	9-Octadecenal, (Z)-
			$C_{17}H_{33}Cl$	7-Heptadecene
			$C_{18}H_{34}O$	13-Octadecenal, (Z)-
			$C_{16}H_{30}O$	cis-9-Hexadecenal
25	38.038	17.25	$C_{16}H_{28}O_2$	Oxacycloheptadec-7-en-2-one
			$C_{21}H_{38}O_2$	11,14-Eicosadienoic acid, methyl ester
			$C_{16}H_{28}O_2$	Oxacycloheptadec-8-en-2-one
			$C_{18}H_{32}O_2$	9,12-Octadecadienoic acid (Z,Z)-
			$C_{20}H_{38}$	5-Eicosyne
26	38.138	8.22	$C_{19}H_{32}O_2$	9,12,15-Octadecatrienoic acid, methyl ester, (Z,Z,Z)-
			$C_{16}H_{26}O$	cis,cis,cis-7,10,13-Hexadecatrienal
			$C_{21}H_{36}O_2$	11,14,17-Eicosatrienoic acid, methyl ester
			$C_{14}H_{24}$	1-Tetradecen-3-yne
			$C_{17}H_{30}$	3-Heptadecen-5-yne, (Z)-
27	38.316	2.42	$C_{14}H_{28}O_2$	Tetradecanoic acid
			$C_{15}H_{30}O_2$	Pentadecanoic acid
			$C_{16}H_{32}O_2$	Palmitic acid
28	38.443	10.1	$C_{20}H_{36}O_2$	Linoleic acid ethyl ester
			$C_{19}H_{34}O_2$	9,12-Octadecadienoic acid, methyl ester
			$C_{20}H_{38}O_2$	Ethanol, 2-(9,12-octadecadienyloxy)-, (Z,Z)-

29	38.552	4.81	$C_{16}H_{26}O$	cis,cis,cis-7,10,13-Hexadecatrienal
			$C_{19}H_{32}O_2$	9,12,15-Octadecatrienoic acid, methyl ester, (Z,Z,Z)-
			$C_{20}H_{34}O_2$	9,12,15-Octadecatrienoic acid, ethyl ester, (Z,Z,Z)-
			$C_{21}H_{36}O_2$	11,14,17-Eicosatrienoic acid, methyl ester
30	39.05	0.56	$C_{17}H_{34}O_2$	Pentadecanoic acid, ethyl ester
			$C_{16}H_{32}O_2$	Myristate <ethyl->;
			$C_{20}H_{40}O_2$	Stearate <ethyl->; Octadecanoic acid <ethyl-> ester
			$C_{18}H_{36}O_2$	Palmitate <ethyl->; Hexadecanoic acid <ethyl-> ester
			$C_{14}H_{28}O_2$	Laurate <ethyl->; Dodecanoic acid <ethyl-> ester
31	39.832	1.21	$C_7H_{13}NO_2$	3-Pyrrolidin-2-yl-propionic acid
			$C_{12}H_{22}N_4$	3-[1-Aziridyl]butyraldehyde azine
			$C_{10}H_{15}N_3O_3$	Pyrrolo[1,2-a]pyrazine-3-propanamide, 2,3,6,7,8,8a-hexahydro-1,4-dioxo-
			$C_8H_{14}N_2O$	5-Pyrrolidino-2-pyrrolidone
			$C_{20}H_{37}N_3O$	1H-1,2,4-Triazole, 1-octadecanoyl-
32	41.018	1.35	unknown	
33	41.533	0.99	$C_{16}H_{50}O_7Si_8$	Octasiloxane, 1,1,3,3,5,5,7,7,9,9,11,11,13,13,15,15-hexadecamethyl
			$C_{16}H_{48}O_6Si_7$	Heptasiloxane, hexadecamethyl-
			$C_{13}H_{40}O_5Si_6$	1,1,1,5,7,7,7-Heptamethyl-3,3-bis(trimethylsiloxy)tetrasiloxane
			$C_{14}H_{44}O_6Si_7$	Heptasiloxane, 1,1,3,3,5,5,7,7,9,9,11,11,13,13-tetradecamethyl-
			$C_{18}H_{54}O_9Si_9$	Cyclononasiloxane, octadecamethyl-
34	43.492	0.65	unknown	
35	43.808	0.61	$C_{16}H_{48}O_6Si_7$	Heptasiloxane, hexadecamethyl-
			$C_{18}H_{54}O_9Si_9$	Cyclononasiloxane, octadecamethyl-
			$C_{16}H_{50}O_7Si_8$	Octasiloxane, 1,1,3,3,5,5,7,7,9,9,11,11,13,13,15,15-hexadecamethyl-
36	44.906	0.93	$C_{24}H_{38}O_4$	Di-n-octyl phthalate
			$C_{24}H_{38}O_4$	1,2-Benzenedicarboxylic acid, diisooctyl ester

			$C_{16}H_{22}O_4$	1,2-Benzenedicarboxylic acid, mono(2-ethylhexyl) ester

Table S2. The GC/MS analysis results of CMR extract B, including retention times and peak area percentages for the detected compounds.

**Table 2**	**extract B**			
**Peak**	**Retention Time**	**Area%**	**Formula**	**Identification**
1	5.033	1.62	unknown	
2	5.344	1.78	$C_5H_6O_2$	Furfuryl alcohol; Furan-2-methanol; 3-Furanmethanol; 2-Furanmethanol
3	5.535	5.09	$C_5H_4O_2$	Furfural; Furan-2-carboxaldehyde
4	5.688	5.68	$C_7H_{16}O$	2-Hexanol, 2-methyl-; 2-Pentanol, 2,4-dimethyl-; 2-Pentanol, 2,3-dimethyl-
5	5.918	13.93	$C_5 H_6 O_2$	Furfuryl alcohol; Furan-2-methanol; 3-Furanmethanol; 2-Furanmethanol
6	6.301	1.47	unknown	
7	6.938	1.5	$C_6H_6O_3$	Furfuryl formate
8	7.918	2.61	$C_8H_{22}O_3Si_2$	Disiloxane, 1,3-diethoxy-1,1,3,3-tetramethyl-
9	8.768	2.35	unknown	
10	8.825	1.77	unknown	
11	9.115	2.23	unknown	
12	9.261	1.43	unknown	
13	9.548	1.28	unknown	
14	9.814	2.81	$C_4H_4O_3$	Succinic anhydride
			$C_6H_8O_3$	3,4-Dimethyldihydrofuran-2,5-dione
			$C_6H_8O_4$	1,4-Dioxane-2,5-dione, 3,6-dimethyl-, (3S-cis)-
			$C_5H_{10}O$	Oxetane, 3,3-dimethyl-
15	11.765	8.02	$C_6H_6O_3$	Furyl hydroxymethyl ketone; Methyl 2-furoate
			$C_5H_6N_2O_2$	2-Furancarboxylic acid, hydrazide
16	12.643	6.82	$C_6H_6O_3$	Levoglucosenone
			$C_7H_{10}O_2$	Cyclohexane-1,2-dione <3-methyl->;
17	15.902	1.47	unknown	
18	16.323	12.71	$C_6H_6O_3$	Hydroxy methyl furfural; 2-Furancarboxaldehyde, 5-(hydroxymethyl)-
			$C_7H_{10}S$	Thiophene, 2-propyl-
			$C_8H_{14}O$	Furan, 2,3-dihydro-4-(1-methylpropyl)-, (S)-
19	16.633	1.22	unknown	

20	19.241	1.25	unknown	
21	25.517	1.3	unknown	
22	29.85	1.64	$C_{19}H_{38}O_2$	Oxirane, [(hexadecyloxy)methyl]-
			$C_{15}H_{30}O_2$	Oxirane, [(dodecyloxy)methyl]-
			$C_{19}H_{40}O_2$	2-Propanol, 1-(hexadecyloxy)-
			$C_{20}H_{42}O_2$	Ethanol, 2-(octadecyloxy)-
			$C_{14}H_{30}O$	2-Ethyl-1-dodecanol
23	34.518	2.98	$C_{16}H_{32}O_2$	Palmitic acid; Hexadecanoic acid
			$C_{13}H_{26}O_2$	Tridecanoic acid
			$C_{14}H_{28}O_2$	Tetradecanoic acid
			$C_{15}H_{30}O_2$	Pentadecanoic acid
24	37.767	1.41	unknown	
25	37.907	3.17	$C_{16}H_{28}O_2$	Oxacycloheptadec-7-en-2-one
			$C_{14}H_{26}O$	9,12-Tetradecadien-1-ol, (Z,E)-
			$C_{18}H_{32}O$	9,17-Octadecadienal, (Z)-
26	38.466	1.5	unknown	
27	44.44	1.98	unknown	
28	44.595	5.74	$C_{18}H_{30}O_2$	5H-3,5a-Epoxynaphth[2,1-c]oxepin, dodecahydro-3,8,8,11a-tetramethyl-, [3S-(3.alpha.,5a.alpha.,7a.alpha.,11a.beta.,11b.alpha.)]-
			$C_{17}H_{26}O_3$	Acetic acid, 3-hydroxy-7-isopropenyl-1,4a-dimethyl-2,3,4,4a,5,6,7,8-octahydronaphthalen-2-yl este
			$C_{15}H_{22}O$	6-Isopropenyl-4,8a-dimethyl-4a,5,6,7,8,8a-hexahydro-1H-naphthalen-2-one
29	44.743	1.57	unknown	
30	45.617	1.67	unknown	
